# Design and Synthesis of CNS-targeted Flavones and Analogues with Neuroprotective Potential Against H_2_O_2_- and Aβ_1-42_-Induced Toxicity in SH-SY5Y Human Neuroblastoma Cells

**DOI:** 10.3390/ph12020098

**Published:** 2019-06-21

**Authors:** Ana M. de Matos, Alice Martins, Teresa Man, David Evans, Magnus Walter, Maria Conceição Oliveira, Óscar López, José G. Fernandez-Bolaños, Philipp Dätwyler, Beat Ernst, M. Paula Macedo, Marialessandra Contino, Nicola A. Colabufo, Amélia P. Rauter

**Affiliations:** 1 Center of Chemistry and Biochemistry, Faculdade de Ciências, Universidade de Lisboa, Ed. C8, Campo Grande, 1749-016 Lisboa, Portugal; amjgmatos@gmail.com (A.M.d.M); aimartins@fc.ul.pt (A.M.); 2 Centro de Química Estrutural, Faculdade de Ciências, Universidade de Lisboa, Ed. C8, Campo Grande, 1749-016 Lisboa, Portugal; 3 MEDIR: Metabolic Disorders, CEDOC Chronic Diseases, Nova Medical School, Campus Sant’Ana, Rua Câmara Pestana, 6, Lab 3.8, 1150-082 Lisboa, Portugal; paula.macedo@nms.unl.pt; 4 Department of Chemistry, Erl Wood Manor, Eli Lilly, Windlesham, Surrey GU20 6PH, UK; man_teresa@lilly.com (T.M.); evans_david_de@lilly.com (D.E.); 5 Abbvie Germany, Knollstr. 51, 67061 Ludwigshafen, Germany; magnus.walter@abbvie.com; 6 Centro de Química Estrutural, Instiuto Superior Técnico, Ulisboa, Av. Rovisco Pais, 1049-001 Lisboa, Portugal; conceicao.oliveira@tecnico.ulisboa.pt; 7 Departamento de Química Orgánica, Facultad de Química, Universidad de Sevilla, Apartado 1203, E-41071 Sevilla, Spain; osc-lopez@us.es (Ó.L.); bolanos@us.es (J.G.F.-B.); 8 Department of Pharmaceutical Sciences, University of Basel, Klingelbergstrasse 50, CH-4056 Basel, Switzerland; philipp.daetwyler@roche.com (P.D.); beat.ernst@unibas.ch (B.E.); 9 Department of Medical Sciences, IBIMED, Universidade de Aveiro, 3810-193 Aveiro, Portugal; 10 APDP-ERC, APDP-Diabetes Portugal, Rua do Salitre, Nº 118-120, 1250-203 Lisboa, Portugal; 11 Dipartimento di Farmacia-Scienze del Farmaco, Università degli Studi di Bari/Biofordrug, Via Edoardo Orabona, 4-70125 Bari, Italy; marialessandra.contino@uniba.it (M.C.); nicolaantonio.colabufo@uniba.it (N.A.C.)

**Keywords:** Alzheimer’s disease, Aβ_1-42_, cholinesterase inhibitors, flavones, chromen-4-ones, C-glucosyl flavonoids, PAMPA

## Abstract

With the lack of available drugs able to prevent the progression of Alzheimer’s disease (AD), the discovery of new neuroprotective treatments able to rescue neurons from cell injury is presently a matter of extreme importance and urgency. Here, we were inspired by the widely reported potential of natural flavonoids to build a library of novel flavones, chromen-4-ones and their *C*-glucosyl derivatives, and to explore their ability as neuroprotective agents with suitable pharmacokinetic profiles. All compounds were firstly evaluated in a parallel artificial membrane permeability assay (PAMPA) to assess their effective permeability across biological membranes, namely the blood-brain barrier (BBB). With this test, we aimed not only at assessing if our candidates would be well-distributed, but also at rationalizing the influence of the sugar moiety on the physicochemical properties. To complement our analysis, log*D*_7.4_ was determined. From all screened compounds, the *p*-morpholinyl flavones stood out for their ability to fully rescue SH-SY5Y human neuroblastoma cells against both H_2_O_2_- and Aβ_1-42_-induced cell death. Cholinesterase inhibition was also evaluated, and modest inhibitory activities were found. This work highlights the potential of *C*-glucosylflavones as neuroprotective agents, and presents the *p*-morpholinyl *C*-glucosylflavone **37**, which did not show any cytotoxicity towards HepG2 and Caco-2 cells at 100 μM, as a new lead structure for further development against AD.

## 1. Introduction

Alzheimer’s disease (AD) is a chronic neurodegenerative condition currently affecting more than 40 million people worldwide [1]. Age, genetic background, and type 2 diabetes (T2D) are well-established risk factors for the development of this pathology, which leads to severe memory impairment, language problems, extreme apathy, unpremeditated aggression, and delusional symptoms [2]. The loss of independence in the performance of the simplest tasks is a major stress factor not only for AD patients, but also for relatives and caregivers. But more importantly, with no drugs being able to stop disease progression [3,4], the hope and quality of life for people living with AD is inevitably compromised unless new effective therapies are rapidly discovered.

**Figure 1:**
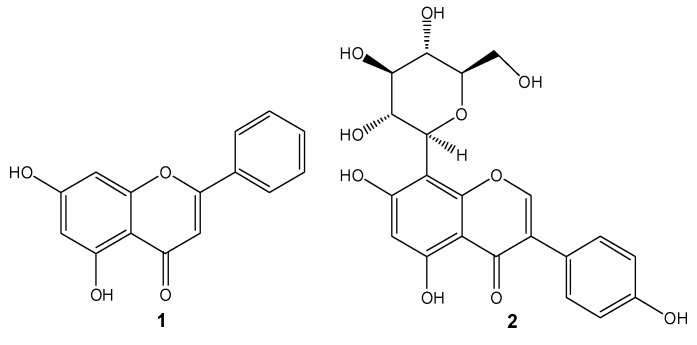
Chemical structure of chrysin (**1**) and 8-β-d-glucosylgenistein (**2**), two natural flavonoids with potential against Alzheimer’s disease (AD). Chrysin was used as the prototype structure for chemical modification in the present work.

At the molecular level, AD is a multifactorial disease [5]. Though the amyloid β (Aβ) protein is commonly placed in the center of AD aetiology, the scientific community has been conducting research to link Aβ with many other molecular players and processes known to contribute to disease development and progression, including the cellular prion protein (PrP^C^) [6], tau hyperphosphorylation [7], oxidative stress, neuroinflammation [8], and insulin resistance [9], among others. Natural products, including flavonoids and their *C*-glucosyl derivatives, have been widely studied and found to interfere with one or more of these features [10]. Examples include chrysin (**1**) and 8-β-d-glucosylgenistein (**2**) (Figure 1). Both compounds have been studied by our group and were found to inhibit the formation of small Aβ_1-42_ oligomers [11] or to interact with Aβ_1-42_ peptides [12], respectively.

In this work, we were inspired by the therapeutic potential of flavonoids against AD, having chrysin (**1**) as the lead structure [11] We focused on projecting and generating a small library of flavonoids with neuroprotective potential, while displaying a suitable physicochemical profile. For that purpose, we used the 5,7-dihydroxychromen-4-one unit as the basic building block of all flavonoid structures (aglycones and *C*-glucosyl derivatives) for two reasons: (a) it is present in many flavonoids exhibiting neuroprotective activities, including not only chrysin [13,14], but also apigenin [15], luteolin [16], and vitexin [17,18], among others, and (b) its synthetic precursor, 2,4,6-trihydroxyacetophenone, has the ideal electron-donating capacity to act as the sugar acceptor in C-glycosylation reactions, in contrast with other polyphenols such as hydroquinone or catechol, which favor the accomplishment of highly effective synthetic routes. 

In the light of our previously published data [11], we planned on executing a thorough replacement of the substituent at C-2 of the 5,7-dihydroxychromen-4-one core, with the ultimate goal of generating new compounds with potential for the establishment of interactions with Aβ_1-42_. Furthermore, by synthesizing a number of flavonoids and their respective *C*-glucosyl derivatives, we were aiming at creating a substantial pool of data that would help us to understand the influence of the sugar moiety in their activity and/or bioavailability. Indeed, having been reported as good stabilizers of non-amyloidogenic Aβ aggregates when combined with polyphenol aglycones [19], sugars may also potentiate antioxidant and antidiabetic activities of these flavonoids [20], which is also an important feature in light of the well-established relationship between AD and type 2 diabetes (T2D) [21]. By screening our compounds against H_2_O_2_- and Aβ_1-42_-induced cell death in SH-SY5Y human neuroblastoma cells, we have herein established a concise structure–activity relationship study on the neuroprotective effects of the molecular scaffold under investigation, focusing on the substituent at C-2 and the importance of the sugar moiety.

## 2. Results

*Database assembly and compound selection*. Following the same synthetic route developed for chrysin (**1**) in a previous study [11], we were now interested in the base-catalysed Claisen–Schmidt aldol condensation reaction for introducing structural diversity into the new flavone analogues. On the basis of the commercially available aldehydes, we then generated a database collection of 98 compounds (the aglycones), which were submitted to a selection process using the Central Nervous System (CNS)-MultiParameter Optimization (MPO) algorithm [22]. This mathematical tool enables the alignment of six key drug-like attributes: partition coefficient (ClogP), distribution coefficient (ClogD), acidity constant (pKa), molecular weight (MW), topological polar surface area (TPSA), and the number of hydrogen bond donors (HBD). Once estimated, these parameters are processed by the algorithm, which generates a desirability score in a scale from 1 to 6. This process allows the selection of compounds with chemical features that make them the most suitable to enter the central nervous system, while displaying favorable permeability, P-gp efflux, metabolic stability, and safety [22]. 

Using the Molecular Operating Environment (MOE) software, the required physicochemical parameters were calculated for each database compound and were subsequently processed through the CNS-MPO algorithm to generate a set of output scores. According to the creators of this tool, only molecules having a CNS-MPO desirability score above 4 are adequate for further development. Following this restriction, a diverse group of flavone analogues with different alkyl, aryl, and heteroaryl substituents at C-2 was selected (see Supplementary Materials, Table S1) and synthesized.

**Figure 2:**
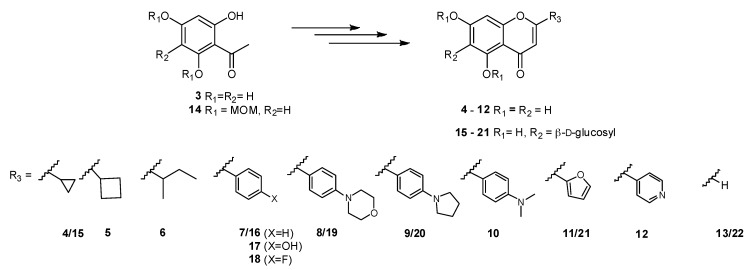
Structure of the new chromones, flavones and their *C*-glucosyl derivatives studied in this work (for the synthetic approach followed, see Supplementary Materials).

*Synthesis*. Chromones and flavones were prepared starting from MOM-diprotected acetophenone **14** (see Supplementary Materials, Scheme S1), which base-catalysed Claisen–Schmidt aldol condensation reaction with commercially available aldehydesgenerated chalcone and chalcone analogue intermediates in very good reaction yields (63–95%). Interestingly, the isomerization acyclic/cyclic product, resulting from chalcone/flavanone equilibrium, could be detected by liquid chromatography-mass spectrometry (LCMS) and the flavanone was the single product isolated, by reaction of the starting material with cyclobutylcarboxaldehyde (see Supplementary Materials). Subsequently, chalcones and flavanones were submitted to iodine-promoted oxidation in pyridine, followed by *p*-TsOH catalyzed deprotection to give the final products **4**–**12** in yields ranging from 38% to 95% (Figure 2 and Supplementary Materials). 

Chrysin (**1**) and 5,7-dihydroxychromen-4-one (**13**) were also synthesized, as described in a previous study published by our group [11], with the purpose of bioactivity comparison.

For the generation of *C*-glucosylflavones and analogues, acetophenone C-glucosylation and selective benzylation were carried out prior to the aldol condensation step, as previously described^12^ (Scheme S2, Supplementary Materials). Then, aldol condensation gave the intermediate chalcones that reacted with iodine and were debenzylated with BCl_3_ at a low temperature to afford the target glucosylchromones and glucosylflavones **15**–**21** (see Figure 2 and Supplementary Materials for compound synthesis, and structure elucidation of intermediate compounds). Notably, some of the target glucosylflavones could not be obtained, either due to the high reactivity of the intermediates, or due to the extreme hydrophilic character of the final product, as in the case of the 2-(pyridin-4-yl)chromone, which made purification virtually unfeasible even when using reverse phase column purification techniques such as HPLC.

The formation of 6-glucosyl-5,7-dihydroxychromen-4-one (**22**) required a different methodology as that described for its analogues. For this task, we applied the same protocol used for generating its aglycone. Dibenzylated acetophloroglucinol derivatized with a perbenzylglucosyl group (Scheme S2B, Supplementary Materials) reacted with sodium hydride in ethyl formate at 0 °C to give an intermediate, that was subsequently dehydrated in acid medium, under reflux, affording perbenzylglucosylchromone in 84% yield. Further deprotection with BCl_3_ in dichloromethane at a low temperature gave compound **22** in good yield (Scheme S2B in Supplementary Materials, that also include intermediates’ synthesis and structure elucidation).

*Parallel Artificial Membrane Permeability Assay (PAMPA) and log D_7.4_ determination*. Finding new molecules exhibiting adequate physicochemical properties for permeating the blood-brain barrier is often a challenging drawback in CNS drug discovery. Indeed, in order to fully evaluate the therapeutic potential of CNS-targeted compounds, such as those generated in the present study, the evaluation of their physicochemical and pharmacokinetic profiles is key. Hence, all synthesized compounds were tested in the parallel artificial membrane permeability assay (PAMPA) in order to measure and rationalize their potential to cross membrane barriers. Testosterone was used as the positive control in this assay. To complete our analysis, the partition coefficient at physiological pH (log*D*_7.4_) was also determined. Ideally, log *D* values should be located between 1 and 4 for a good compromise between solubility and membrane permeability allowing oral availability, good cell permeation, and low metabolic susceptibility [23]. Results are presented in Table 1.

**Figure 3:**
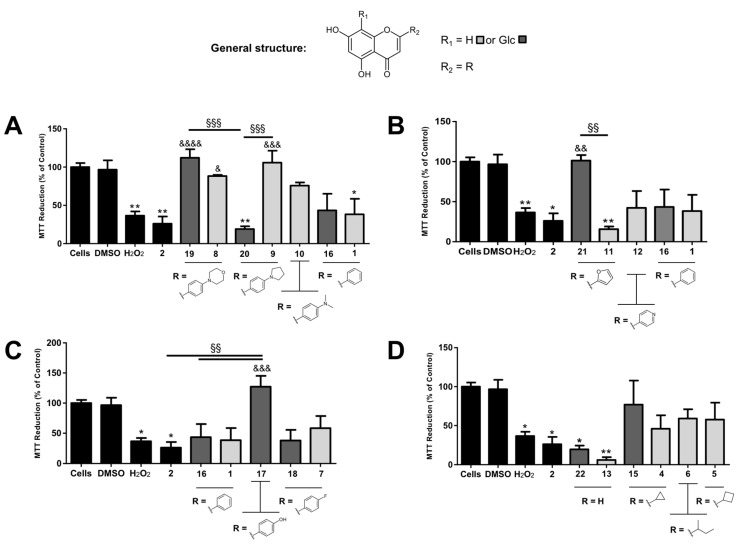
Neuroprotective effects of compound **2**, flavone derivatives and corresponding aglycones against H_2_O_2_-induced toxicity in human SH-SY5Y neuroblastoma cells via a MTT cell viability assay. (**A**) Effects caused by amine moieties in *para*-position of ring B; (**B**) effects caused by the replacement of ring B with heteroaromatic groups; (**C**) effects caused by electron withdrawing groups in *para*-position of ring B; (**D**) effects caused by the replacement of ring B with aliphatic moieties. Cells were incubated with 100 μM H_2_O_2_ for 24 h at 37 °C, in the presence (50 μM) or absence of each compound. The tests were performed in triplicate with a final concentration of 0.5% DMSO. Results are presented as means ± standard error. Statistical differences between groups were assessed by one-way ANOVA followed by a Tukey’s post-test. * *p* < 0.05, and ** *p* < 0.01 versus cell control; ^&^ *p* < 0.05, ^&&^ *p* < 0.01, ^&&&^ *p* < 0.001 and ^&&&&^ *p* < 0.0001 versus H_2_O_2_ control; ^§§^ *p* < 0.01 and ^§§§^ *p* < 0.001 versus another compound.

*Neuroprotective activity assays against H_2_O_2_-induced toxicity*. On the basis of previously described protocols [24,25,26], the flavones and analogues herein synthesized were screened for their neuroprotective effects against H_2_O_2_-induced oxidative stress and neuronal damage in SH-SY5Y human neuroblastoma cells by means of a thiazolyl blue tetrazolium bromide (MTT) reduction-based cell viability assay. This screening protocol aimed not only at comparing the activity of aglycones versus *C*-glucosyl derivatives when tested at the same concentration (50 μM), but also at distinguishing between the best candidates amongst amines, aliphatic or heteroaryl derivatives, or aromatic derivatives with electron-withdrawing substituents. In addition, all compounds were directly compared to compound **2**, a natural molecule conjectured to display neuroprotective effects [12], but with no cell-based evidence reported up to this point. Results are presented in Figure 3. All compounds were tested in the same experimental conditions without the neurotoxic agent beforehand, and none were found to significantly decrease cell viability when compared to non-treated controls (see Supplementary Materials, Table S2).

At 100 μM, H_2_O_2_ caused over 50% of cell viability loss, which is consistent with earlier published data (Figure 3) [25]. While compound **2** was not found to display neuroprotective effects against the observed H_2_O_2_-induced cell death in this cell line, at the tested concentration, amine derivatives were generally well-succeeded in restoring cell viability (Figure 3A). Compounds **8**, **9,** and **19** were in fact able to produce statistically significant differences when compared to H_2_O_2_ controls (*P* < 0.5, *P* < 0.001 and *P* < 0.0001, respectively). *C*-glucosides **17** and **21** were also able to rescue cells from oxidative damage caused by H_2_O_2_ (*P* < 0.001 and *P* < 0.01 respectively) (Figure 3B,C), while neither the remaining glycosides nor the remaining aglycones were able to lead to similar results.

**Figure 4:**
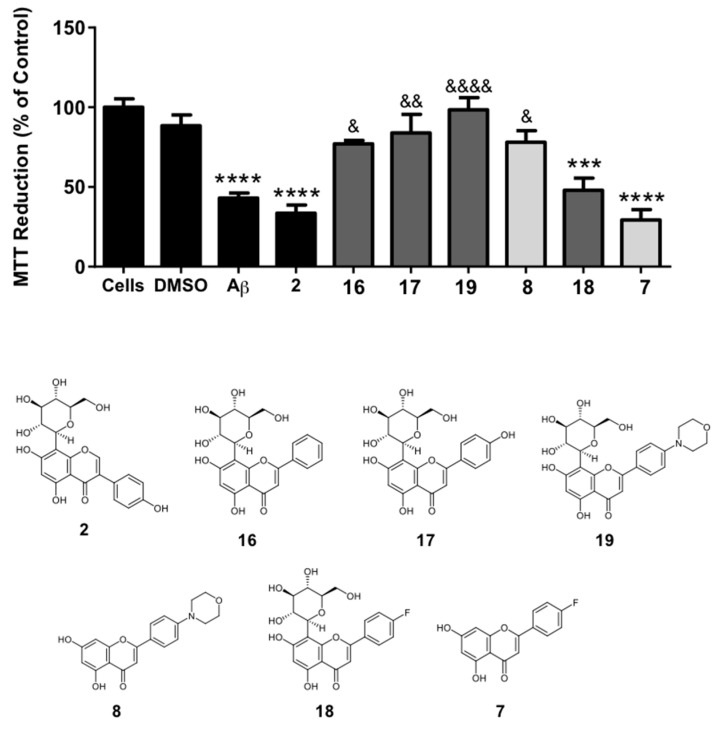
Neuroprotective effects of compound **2** and analogues against Aβ_1-42_-induced toxicity in human SH-SY5Y neuroblastoma cells via a MTT cell viability assay. Cells were incubated with 20 μM Aβ_1-42_ for 24 h at 37 °C, in the presence (50 μM) or absence of each compound. The tests were performed in triplicate with 1% DMSO (Aβ) or 1.5% DMSO (Aβ + compound − maximum DMSO percentage presented in the graph). Results are presented as means ± standard error. Statistical differences between groups were assessed by one-way ANOVA followed by a Tukey’s post-test. *** *p* < 0.001 and **** *p* < 0.0001 versus cell control; ^&^ *p* < 0.05, ^&&^ *p* < 0.01 and ^&&&&^ *p* < 0.0001 Aβ control.

*Neuroprotective activity assays against Aβ_1-42_-induced toxicity*. From all compounds tested in the above presented assay, the pair **8** and **19** presented the strongest neuroprotective potential, with convincing proof on the importance of the aglycone for the desired neuroprotective effects against H_2_O_2_-induced cell injury. Both compounds were henceforth selected to be further explored, together with compound **2**, the *C*-analogue of chrysin (**16**), vitexin (**17**), and the pair of 4’-fluoroflavones (**7** and **18**) for comparison purposes. Here, the MTT assay was used once again to assess cell viability of SH-SY5Y cells treated with 20 μM of Aβ_1-42_, in the presence of 50 μM of each compound. Similar protocols have been used to screen candidates for AD therapy [27,28]. Moreover, based on the existing evidence that *in situ* spontaneous fibrillization of Aβ_1-42_ in the incubation mixture is important in the triggering of neurotoxic effects [29], we added the Aβ_1-42_ peptide fragment dissolved in DMSO to the culture medium prior to incubation, together with each compound in study. Results are displayed in Figure 4. Even though there is published evidence that undifferentiated SH-SY5Y cells are not as sensitive to Aβ_1-42_-induced neurite degeneration and apoptosis as differentiated ones [29], with 20 μM of Aβ_1-42_ we were able to observe a significant decrease in cellular MTT reduction capacity, corresponding to roughly half of the cell viability rates observed in the non-treated control. The 4-morpholinyl derivative **19** exhibited, once more, the result with the highest significance when compared to controls. Compound **8** also displayed relevant neuroprotective effects against Aβ_1-42_, contrarily to the 4’-fluoroflavone **7**.

## 3. Discussion

As predicted by our computational calculations, all aglycones (**1**, **4**–**13**) presented an excellent membrane permeation capacity, as shown by the measured effective permeability for these molecules (Log *P*_e_ > −5.7). Yet, compounds **2** and **10** displayed log *D*_7.4_ values slightly above the desired upper limit, and thus they may be associated with the risk of being retained in cell membranes and/or fat tissues. The data for *C*-glucosyl derivatives **15**–**22** exhibited much more variability. Compounds **15** and **18** displayed no detectable permeability in PAMPA. Even though compounds **16** and **17** have a slightly improved effective permeability (Log *P*_e_ ~ −9), it is still very limited to allow passive diffusion over membranes. Notably, the 4-pyrrolidinyl derivative (**38**) presented the best effective permeability from all *C*-glucosylflavones (log *P*_e_ = −6.520 ± 0.408) and a determined log *D*_7.4_ (1.8 ± 0.2) within the ideal value range. Compounds **19**, **21,** and **22** fell in the middle (log *P*_e_ ~ −7); however, only the 4-morpholinyl derivative **19** had a desirable log *D*_7.4_ value (1.8 ± 0.2). Indeed, it seems that only certain aglycones are able to compensate for the hydrophilicity of the sugar moiety, either owing to their intrinsic lipophilicity, or to the resulting molecular conformation of the *C*-glycoside as a whole. Nevertheless, it is important to mention that, as previously described for other *C*-glucosyl flavonoids [30], the glucosyl moiety in these compounds might prompt their affinity towards GLUT-1 transporters in the blood-brain barrier (BBB), which may ultimately contribute to enhance their concentration near the therapeutic targets in the CNS.

The neuroprotective activity of many lead candidates, especially from natural origin, has been assessed against cellular damage induced by H_2_O_2_ in SH-SY5Y and other relevant neuronal cell lines over the past decades [24,25,26,31]. Indeed, this neurotoxic agent has been reported to cause mitochondrial and cell membrane damage, with the depletion of antioxidant enzymatic machinery leading to increased levels of reactive oxygen species (ROS) and associated neuroinflammatory processes [25]. Ultimately, H_2_O_2_-induced oxidative stress and inflammation trigger neuronal apoptosis through increased expression of pro-apoptotic factors such as caspase 3, and depletion of anti-apoptotic factors such as Bcl-2, which have been linked to the development and progression of AD [25,32]. As described for melittin, orientin, and bikaverin, among others, protection mechanisms offered by small molecules against H_2_O_2_-promoted cell damage and apoptosis may include the downregulation of pro-inflammatory transcription factors such as the nuclear factor-kappaB (NFκB), increased Bcl-2 mRNA and protein expression, inhibition of caspase expression and activity, and replenishment of neuronal pro-oxidative/antioxidant enzyme balance [24,25,26]. In addition, provided that Aβ oligomers have been described to enhance ROS levels and protein oxidation in neurons [33], it is important to bear in mind that other possible neuroprotective mechanisms with impacts on oxidative stress can be associated with the inhibition of Aβ oligomerization or regulation of Aβ production and/or clearance itself.

Compounds **8**, **9,** and **19** were able to produce statistically significant differences when compared to H_2_O_2_ controls. What’s more, the amine moiety was found to be critical for the neuroprotective activity, as shown by the absence of significant effects displayed by chrysin (**1**) and its *C*-glucosyl analogue **16**. Yet, it is herein important to distinguish between aglycones and *C*-glucosyl derivatives, as a consistent correlation between the presence of the sugar moiety and stimulation of cell survival could not be observed. If, on the one hand, amine aglycones **8** and **9** were able to cause significant improvements in cell viability when compared to controls, on the other hand only compound **19** produced the desired effect, with dramatic differences from that of its 4-pyrrolidinyl analogue **20**. Since both aglycones **8** and **9** are active, it is likely that the *C*-glucosyl moiety is detrimental in the second case. Conversely, the fact that the 4-morpholinyl group in compounds **8** and **19** induced protective effects regardless of the presence of the sugar moiety could mean that this substituent, perhaps through the presence of an endocyclic oxygen atom, is preferred for this type of activity. Interestingly, aglycone **10** was not significantly active, reinforcing that the presence of a nitrogen-containing ring in *para*-position of ring B should, indeed, be beneficial. All in all, this group of derivatives presented a major improvement when compared to compound **2**, and both unsubstituted flavones **1** and **16**, with compounds **8** and **19** as the most promising pair of flavone derivatives.

From the heteroaryl derivatives, only the *C*-glucosyl flavone analogue containing the 2-furanyl moiety **21** was able to produce a significant improvement in cell viability, contrarily to its aglycone **11** or the 4-pyridinyl derivative **12** (Figure 3B). Contrasting with the pair of 4-pyrrolidinyl derivatives (**9** and **20**, Figure 3A), in the case of compounds **11** and **21** it appears to be the combination of the sugar moiety with the modified aglycone that triggers the neuroprotective effect, and not the aglycone per se. Still, this combination was able to improve the activity of the *C*-glucosyl analogue of chrysin **16**, indicating that the replacement of ring B with a heteroaromatic ring in the presence of the sugar moiety may be advantageous. Ultimately, compound **21** succeeded at improving the activity of the lead compound **2**.

Regarding the compounds with electron-withdrawing groups in *para*-position of ring B (Figure 3C), it is interesting to note that vitexin (**17**) was able to fully recover cell viability, while compound **2** and *C*-glucosylchrysin (**16**) were not. These results indicate that the *C*-glucosylflavone scaffold may be more effective in terms of neuroprotective effects than the corresponding isoflavone, and that the hydroxy group in *para*-position of ring B is in fact beneficial for activity. What’s more, from both electron withdrawing groups in *para*-position of ring B (OH- in vitexin and F- in derivatives **18** and **7**) only the hydroxy group was able to produce a relevant neuroprotective effect, which may be attributable to: (a) the formation of a phenoxyl radical stabilized by resonance due to the presence of the α,β-unsaturated ketone if the neuroprotective effect results from direct chemical ROS inactivation, or (b) the formation of hydrogen bonds with a macromolecular target, e.g., an antioxidant enzyme.

Accordingly, no significant differences between cell viability in the presence of either of the two 5,7-dihydroxychromen-4-ones (**22** and **13**) and the cell viability of H_2_O_2_ controls were observed in our study (Figure 3D). Even though all compounds with aliphatic substituents (**4**, **5,** and **6** led to an increase in MTT reduction when compared to both chromen-4-ones, the increase was not significant. 

From the compounds selected to be tested against Aβ_1-42_-induced neurotoxicity, the 4-morpholinyl derivative **19** again presented the result with the highest significance when compared to the controls. Compound **8** also displayed relevant neuroprotective effects against Aβ_1-42_ (contrarily to the 4’-fluoroflavone **7**) which once more supports the hypothesis that in this particular case, compound **8** is active per se, but its effects are maintained or potentiated by the presence of the sugar moiety. Provided that the pair of compounds **8** and **19** was active in H_2_O_2_-induced toxicity assay as well, whether the observed effects against Aβ_1-42_ are due to a direct interaction with Aβ oligomers or to the inhibition of Aβ_1-42_-promoted oxidative stress (or both) remains to be clarified. Nonetheless, compound **19**, the best in the series of flavones and analogues presented in this work, also showed some inhibitory activity of acetylcholinesterase (18% at 100 μM, see Supplementary Materials, Table S3). Moreover, all compounds tested inhibited either acetylcholinesterase (AChE) and/or butyrylcholinesterase (BuChE). Chrysin was the most active butyrylcholinesterase inhibitor, while amongst the selective BuChE inhibitors, the chromen-4-one derivative **6** was indeed the most promising one. Nonetheless, compound **19** stood out as the lead molecule with potential for AD. It was tested in human epithelial colorectal adenocarcinoma (Caco-2) and liver hepatocarcinoma (HepG2) cells from concentrations ranging from 0.1 μM to 100 μM, and no significant cytotoxic effects could be observed (IC_50_ ≥ 100 μM).

## 4. Materials and Methods

*General Methods.* HPLC grade solvents and reagents were obtained from commercial suppliers and were used without further purification. Chrysin (**1**), compound **2,** and 5,7-dihydroxychromen-4-one (**13**) were synthesized according to the methodologies previously described by us.^11,12^ LCMS experiments were performed in a column XBridge C18 3.5u 2.1 × 50 mm at 1.2 mL/min and 50 °C; 10 mM ammonium bicarbonate pH 9/ACN, gradient 10 > 95% ACN in 1.5 min + 0.5 min hold. Reactions affording compounds **37** and **17** were followed by TLC, carried out on aluminum sheets (20 × 20 cm) coated with silica gel 60 F-254, 0.2 mm thick (Merck, Darmstadt, Germany) with detection by charring with 10% H_2_SO_4_ in ethanol. Flash column chromatography was performed using CombiFlash^®^ Rf200 (Teledyne Isco, Lincoln, CA, USA). Preparative HPLC was performed in a Gilson apparatus using either Phenomenex Gemini NX, C18, 5 μm 30 × 100 mm or Phenomenex Gemini NX, C18, 10 μm 50 × 150 mm columns. NMR spectra for compound characterization were recorded on a Bruker AV III HD Nanobay spectrometer running at 400.13 MHz equipped with a room temperature 5 mm BBO Smartprobe. Chemical shifts are expressed in δ (ppm) and the proton coupling constants *J* in Hertz (Hz). NMR data were assigned using appropriate COSY, DEPT, HMQC, and HMBC spectra (representative examples are provided in the Supporting information appendix). Optical rotations were measured with a Perkin–Elmer 343 polarimeter. Melting points were measured using a Stuart SMP30 melting point apparatus. High-resolution mass spectra of final compounds were acquired on a Bruker Daltonics HR QqTOF Impact II mass spectrometer (Billerica, MA, USA). The nebulizer gas (N_2_) pressure was set to 1.4 bar, and the drying gas (N_2_) flow rate was set to 4.0 L/minute at a temperature of 200 °C. The capillary voltage was set to 4500 V and the charging voltage was set to 2000 V. Tested compounds have ≥ 95% purity as determined by LCMS. Synthesis of intermediate compounds **23**–**31** and **35**–**42** is reported in Supplementary Materials, together with their LCMS data, physical and NMR data.

*General procedure for the synthesis of non-glycosylated chalcones*/*flavones.* Each compound **23**–**30** was dissolved in dry pyridine (0.248 mmol in 7.33 mL). Then, catalytic amounts of I_2_ (0.087 mmol, 0.35 eq.) were added and the mixture was stirred under reflux for 24 h–72 h. All reactions were followed by LCMS. Once the starting material was fully consumed, the mixture was allowed to reach room temperature and the pyridine was co-evaporated with toluene under reduced pressure. The residue was resuspended in dichloromethane, washed first with a saturated solution of sodium thiosulfate, and then with brine. The flavone was extracted with dichloromethane (3 × 30 mL), dried over MgSO_4_, and the solution filtered and concentrated under vacuum. The residue was then resuspended in ethanol (15 mL) and *p*-TsOH (12% in AcOH, 0.1 mL) was added. The reaction was stirred under reflux for 2–24 h. After having reached completion by LCMS, the solvent was evaporated under vacuum and the residue purified using the most adequate purification method(s) to afford compounds **4**–**12.**

*2-Cyclopropyl-5,7-dihydroxy-4*H*-chromen-4-one* (**4**). Purified by preparative HPLC. Reaction yield over two steps: 53%; LCMS: RT  =  0.52 min, *m*/*z*  =  219.0 [M + H]^+^ (high pH method); white solid; m.p. = 201.2–202.9 °C. ^1^H NMR (MeOD) δ (ppm) 6.26 (d, 1H, *J*_meta_ = 2.1 Hz, H-8), 6.17 (d, 1H, *J*_meta_ = 2.1 Hz, H-6), 6.10 (s, 1H, H-3), 2.02–1.96 (m, 1H, H-1′), 1.18–1.09 (m, 4H, H-2′, H-3′). ^13^C NMR [MeOD] δ (ppm) 181.5 (C-4), 173.5 (C-2), 166.0 (C-7), 163.4 (C-5), 159.6 (C-8a), 106.5 (C-3), 100.2 (C-6), 95.0 (C-8), 15.4 (C-1′), 9.5 (C-2′, C-3′). HRMS-ESI (*m*/*z*): [M + H]^+^ calcd for C_12_H_11_O_4_ 219.0652, found 219.0642.

*2-Cyclobutyl-5,7-dihydroxy-4*H*-chromen-4-one* (**5**). Purified by preparative HPLC. Reaction yield over two steps: 85%; LCMS: RT  =  0.72 min, *m*/*z*  =  233.0 [M + H]^+^ (high pH method); white solid; m.p. = 200.3–201.5 °C. ^1^H NMR (MeOD) δ (ppm) 6.34 (d, 1H, *J*_meta_ = 2.1 Hz, H-8), 6.18 (d, 1H, *J*_meta_ = 2.1 Hz, H-6), 6.03 (s, 1H, H-3), 3.53 (td, 1H, *J* = 8.0 Hz, H-1′), 2.37–2.31 (m, 4H, H-2′, H-4′), 2.17–2.06 (m, 1H, H-3′a), 1.99–1.91 (m, 1H, H-3′). ^13^C NMR [(CD_3_)OD] δ (ppm) 183.4 (C-4), 174.1 (C-2), 166.0 (C-7), 163.3 (C-5), 159.9 (C-8a), 106.5 (C-3), 100.0 (C-6), 94.9 (C-8), 39.5 (C-1′), 27.5 (C-2′, C-4′), 19.0 (C-3′). HRMS-ESI (*m*/*z*): [M + H]^+^ calcd for C_13_H_13_NO_4_ 233.0808, found 233.0804.

*5,7-Dihydroxy-2-(1-methylpropyl)-4*H*-chromen-4-one* (**6**). Purified by preparative HPLC. Reaction yield over three steps: 67%; LCMS: RT  = 1.10 min, *m*/*z* =  235.0 [M + H]^+^ (low pH method); brown solid; m.p. = 192.5–193.8 °C. ^1^H NMR (MeOD) δ (ppm) 6.33 (d, 1H, *J*_meta_ = 2.2 Hz, H-8), 6.20 (d, 1H, *J*_meta_ = 2.2 Hz, H-6), 6.05 (s, 1H, H-3), 2.65 (sextet, 1H, *J*_1′-1″~1′-2′_ = 7.4 Hz, H-1′), 1.80–1.59 (m, 2H, H-2′), 1.30 (d, 3H, *J*_1″-1′_ = 7.4 Hz, H-1″), 0.95 (t, 3H, *J*_3′-2′_ = 7.4 Hz, H-3′). ^13^C NMR [MeOD] δ (ppm) 184.1 (C-4), 175.8 (C-2), 166.0 (C-7), 163.3 (C-5), 159.9 (C-8a), 107.6 (C-3), 105.4 (C-4a), 100.0 (C-6), 94.8 (C-8), 41.7 (C-1′), 28.6 (C-2′), 18.2 (C-1″), 11.9 (C-3′). HRMS-ESI (*m*/*z*): [M + H]^+^ calcd for C_13_H_15_O_4_ 235.0965, found 235.0962.

*4′-Fluoro-5,7-dihydroxyflavone* (**7**). Purified by preparative HPLC. Reaction yield over two steps: 38%; LCMS: RT = 1.10 min, *m*/*z* = 273.0 [M + H]^+^ (low pH method); white solid; m.p. = 264.8–265.5 °C. ^1^H NMR [MeOD] δ (ppm) 8.05 (dd, 2H, *J*_ortho_ = 8.9 Hz, *J*_2′-F=6′-F_ = 5.2 Hz, H-2′, H-6′), 7.31 (t, 2H, *J*_ortho_~*J*_3′-F=5′-F_ = 8.7 Hz, H-3′, H-5′), 6.73 (s, 1H, H-3), 6.50 (d, 1H, *J*_meta_ = 2.2 Hz, 1H, H-8), 6.24 (d, 1H, *J*_meta_ = 2.2 Hz, H-6). ^13^C NMR [MeOD] δ (ppm) 183.8 (C-4), 166.3 (C-7), 164.7 (C-5), 163.3 (d, *J*_C-F_ = 259.3 Hz, C-4′), 159.5 (C-8a), 130.1 (d, *J*_C-F_ = 9.0 Hz, C-2′, C-6′), 127.6 (C-1′), 117.3 (d, *J*_C-F_ = 22.5 Hz, C-3′, C-5′), 106.0 (C-3, C-4a), 100.3 (C-6), 95.2 (C-8). HRMS-ESI (*m*/*z*): [M + H]^+^ calcd for C_15_H_10_FO_4_ 273.0558, found 273.0554.

*5,7-Dihydroxy-4′-(morpholin-4-yl)flavone* (**8**). Purified by preparative HPLC. Reaction yield over two steps: 68%; LCMS: RT = 1.07 min, *m*/*z* = 340.0 [M + H]^+^ (low pH method); orange solid; m.p. = 234.0–235.6 °C. ^1^H NMR (MeOD) δ (ppm) 7.87 (d, 2H, *J*_ortho_ = 9.0 Hz, H-2′ and H-6′), 7.06 (d, 2H, H-3′ and H-5′), 6.58 (s, 1H, H-3), 6.45 (d, 1H, *J_meta_* = 2.2 Hz, H-8), 6.21 (d, 1H, *J_meta_* = 2.1 Hz, H-6), 3.86–3.83 (m, 4H, NCH_2_C*H*_2_O), 3.34 (NC*H*_2_CH_2_O, overlapped with metanol-*d*_4_ peak). ^13^C NMR (MeOD) δ (ppm) 183.8 (C-4), 165.9 (C-2), 163.7 (C-7 and C-5), 159.4.0 (C-8a), 155.3 (C-4′), 128.9 (C-2′, C-6′), 121.8 (C-1′), 115.4 (C-3′, C-5′), 105.3 (C-4a), 103.3 (C-3), 100.1 (C-6), 95.0 (C-8), 67.7 (NCH_2_*C*H_2_O), 48.1 (N*C*H_2_CH_2_O). HRMS-ESI (*m*/*z*): [M + H]^+^ calcd for C_19_H_18_NO_5_ 340.1179, found 340.1175.

*5,7-Dihydroxy-4′-(pyrrolidin-1-yl)flavone* (**9**). Purified by preparative HPLC. Reaction yield over two steps: 95%; LCMS: RT = 1.08 min, *m*/*z* = 324.0 [M + H]^+^ (high pH method); orange solid; m.p. = 282.4–283.6 °C. ^1^H NMR [(CD_3_)_2_CO] δ (ppm) 7.89 (d, 2H, *J*_ortho_ = 9.0 Hz, H-2′ and H-6′), 6.70 (s, 1H, H-3), 6.65 (d, 2H, H-3′ and H-5′), 6.46 (d, 1H, *J_meta_* = 2.2 Hz, H-8), 6.16 (d, 1H, *J_meta_* = 2.2 Hz, H-6), 3.35–3.33 (m, NC*H*_2_CH_2_), 2.00–1.97 (m, 4H, NCH_2_C*H*_2_). ^13^C NMR [(CD_3_)_2_CO] δ (ppm) 181.5 (C-4), 164.6 (C-2), 163.9 (C-7), 161.5 (C-5), 157.2 (C-8a), 150.1 (C-4′), 128.0 (C-2′, C-6′), 116.0 (C-1′), 111.7 (C-3′, C-5′), 103.6 (C-4a), 100.9 (C-3), 98.7 (C-6), 93.9 (C-8), 47.4 (N*C*H_2_CH_2_), 25.0 (NCH_2_*C*H_2_). HRMS-ESI (*m*/*z*): [M + H]^+^ calcd for C_19_H_18_NO_4_ 324.1230, found 324.1225.

*4′-Dimethylamino-5,7-dihydroxyflavone* (**10**). Purified by preparative HPLC. Reaction yield over two steps: 54%; LCMS: RT = 1.12 min, *m*/*z* = 298.0 [M + H]^+^ (low pH method); orange solid; m.p. = 291.3–292.7 °C. ^1^H NMR [(CD_3_)_2_SO] δ (ppm) 13.11 (s, 1H, OH-5), 7.88 (d, 2H, *J*_ortho_ = 9.0 Hz, H-2′ and H-6′), 6.80 (d, 2H, H-3′ and H-5′), 6.71 (s, 1H, H-3), 6.46 (d, 1H, *J_meta_* = 2.1 Hz, H-8), 6.17 (d, 1H, *J_meta_* = 2.1 Hz, H-6), 3.03 [s, 6H, N(C*H*_3_)_2_]. ^13^C NMR [(CD_3_)_2_SO] δ (ppm) 181.5 (C-4), 164.3 (C-2), 163.8 (C-7), 161.4 (C-5), 157.2 (C-8a), 152.6 (C-4′), 127.8 (C-2′, C-6′), 116.5 (C-1′), 111.6 (C-3′, C-5′), 103.6 (C-4a), 101.2 (C-3), 98.6 (C-6), 93.8 (C-8), 39.6 [N(*C*H_3_)_2_]. HRMS-ESI (*m*/*z*): [M + H]^+^ calcd for C_17_H_16_NO_4_ 298.1074, found 298.101.

*2-(Furan-2-yl)-5,7-dihydroxy-4*H*-chromen-4-one* (**11**). Purified by preparative HPLC. Reaction yield over two steps: 59%; LCMS: RT. = 0.99 min, *m*/*z* = 298.0 [M + H]^+^ (low pH method); white solid; m.p. = 239.3–240.8 °C. ^1^H NMR [(CD_3_)_2_CO] δ (ppm) 12.87 (s, 1H, OH-5), 7.92 (d, 1H, *J*_5′-4′_ = 1.6 Hz, H-5′), 7.34 (d, 1H, *J*_4′-3′_ = 3.5 Hz, H-3′), 6.77 (dd, 1H, *J*_5′-4′_ = 3.5 Hz, *J*_3′-4′_ = 1.8 Hz, H-4′), 6.49–6.48 (m, 2H, H-3, H-8), 6.27 (d, 1H, *J_meta_* = 1.8 Hz, H-6). ^13^C NMR [(CD_3_)_2_CO] δ (ppm) 182.7 (C-4), 164.9 (C-2), 156.3 (C-7)*, 163.1 (C-5), 158.0 (C-8a)*, 147.8 (C-5′), 146.9 (C-2′), 114.8 (C-3′), 113.9 (C-4′), 104.0 (C-4a), 103.9 (C-3), 100.1 (C-6), 95.0 (C-8). HRMS-ESI (*m*/*z*): [M + H]^+^ calcd for C_13_H_19_O_5_ 245.0444, found 245.0438. *Permutable signals.

*5,7-Dihydroxy-2-(pyridin-4-yl)-4*H*-chromen-4-one* (**12**). Purified by preparative HPLC. Reaction yield over three steps: 87%; LCMS: RT = 0.43 min, *m*/*z* = 256.0 [M + H]^+^ (low pH method);: orange oil. ^1^H NMR (MeOD) δ (ppm) 8.79 (d, 2H, H-3′, H-5′), 8.37 (d, 2H, *J*_ortho_ = 6.6 Hz, H-2′, H-6′), 6.74 (s, 1H, H-3), 6.30 (d, 1H, *J*_meta_ = 1.8 Hz, H-8), 6.10 (d, 1H, *J*_meta_ = 1.8 Hz, H-6). ^13^C NMR (MeOD) δ (ppm) 180.7 (C-4), 171.1 (C-2), 169.8 (C-7), 161.0 (C-5), 155.6 (C-8a), 151.6 (C-1′), 143.3 (C-3′, C-5′), 128.1 (C-2′, C-6′), 103.4 (C-3), 103.2 (C-4a), 99.6 (C-6), 92.7 (C-8). HRMS-ESI (*m*/*z*): [M + H]^+^ calcd for C_14_H_10_NO_4_ 256.0604, found 256.0600.

*General procedure for the synthesis of C-glucosylflavones.* Each *C*-glucosylchalcone **35**–**41** was dissolved in dry pyridine (0.172 mmol in 5.11 mL). Then, catalytic amounts of I_2_ (0.060 mmol, 0.35 eq.) were added and the mixture was stirred under reflux for 48–72 h. All reactions were followed by LCMS. Once the starting material was fully consumed, the mixture was allowed to reach room temperature and the pyridine was co-evaporated with toluene under reduced pressure. The residue was resuspended in dichloromethane, washed first with a saturated solution of sodium thiosulfate, and then with brine. The flavone was extracted with dichloromethane (3 × 30 mL), dried over MgSO_4_, and the solution filtered and concentrated under vacuum. The residue was then resuspended in extra dry dichloromethane (7.10 mL) and stirred at −78 °C under N_2_ saturated atmosphere. A 1 M solution of BBr_3_ in dichloromethane (1.72 mL, 1.72 mmol, 10 eq.) was added in a dropwise manner over 5 min, and the reaction stirred for 2–4 h. After having reached completion by LCMS, the reaction was quenched with a 1:1 mixture of dichloromethane/methanol (ca. 15 mL) and the reaction was stirred for approximately 20 min at room temperature. The solvent was evaporated under vacuum and the residue purified using the most adequate purification method(s) to afford compounds **15**–**21**.

*2-Cyclopropyl-8-(β-d-glucopyranosyl)-5,7-dihydroxy-4-chromen-4-one* (**15**). Purified by preparative HPLC. Reaction yield over two steps: 37%; LCMS: RT = 0.51 min, *m*/*z* = 381.0 [M + H]^+^ (low pH method); yellowish oil. ^1^H NMR (MeOD) δ (ppm) 6.23 (s, 1H, H-6), 6.20 (s, 1H, H-3), 4.86 (H-1″, overlapped with the methanol-*d*_6_ water peak), 3.92–3.87 (m, 2H, H-2″, H-6″a), 3.75–3.64 (m, 1H, H-6″b), 3.47–3.42 (m, 3H, H-3″, H-4″, H-5″), 2.06–2.00 (m, 1H, H-1′), 1.46–1.43 (m, 1H, H-2′a)*, 1.28–1.22 (m, 1H, H-3′a)*, 1.18–1.09 (m, 2H, H-2′b, H-3′b). ^13^C NMR (MeOD) δ (ppm) 183.6 (C-4), 173.3 (C-2), 164.4 (C-7), 162.7 (C-5), 160.4 (C-8a), 106.8 (C-8), 105.5 (C-3), 104.9 (C-4a), 99.3 (C-6), 82.8 (C-5″), 80.1 (C-3″), 74.9 (C-1″), 73.3 (C-2″), 72.6 (C-4″), 63.4 (C-6″), 15.7 (C-1′), 10.0, 9.6 (C-2′, C-3′). *Permutable signals. HRMS-ESI (*m*/*z*): [M + H]^+^ calcd for C_18_H_21_O_9_ 381.1180, found 381.1176.

*8-(β-d-Glucopyranosyl)-5,7-dihydroxyflavone* (**16**). Purified by preparative HPLC, followed by Isolute SCX-2 column chromatography (Biotage). Reaction yield over two steps: 88%; LCMS: RT = 0.49 min, *m*/*z* = 414.80 [M  − H]^−^ (high pH method); yellow solid; m.p. = 188.1–189.2 °C. ^1^H NMR (MeOD) δ (ppm) 8.13, 8.03 (d, 2H, *J*_ortho_ = 7.1 Hz, H-2′ and H-6′)*, 7.58–7.54 (m, 3H, H-3′, H-4′ and H-5′), 6.75 (s, 1H, H-3), 6.30 (s, 1H, H-6), 5.00 (d, 1H, *J*_1″-2″_ = 9.9 Hz, H-1″)*, 4.11 (t, 1H, *J*_2″-1″~2″-3″_ = 9.3 Hz, H-2″), 3.97 (br d, 1H, *J*_6″a-6″b_ = 12.1 Hz, H-6″a)*, 3.81 (dd, 1H, *J*_6″b-6″a_ = 12.1 Hz, *J*_6″b-5″_ = 5.3 Hz, H-6″a), 3.68 (t, 1H, *J*_4″-3″~4″-5″_ = 9.2 Hz, H-4″), 3.55–3.48 (m, 2H, H-3″ and H-5″). ^13^C NMR (MeOD) δ (ppm) 184.2 (C-4), 166.0 (C-2), 164.8 (C-7), 162.8 (C-5), 158.2 (C-8a), 133.1 (C-3′ and C-5′), 132.8 (C-1′), 130.2 (C-4′), 128.1, 127.8 (C-2′ and C-6′)*, 105.8 (C-3), 105.1 (C-8), 104.6 (C-4a), 99.6 (C-6), 82.9 (C-5″), 80.2 (C-3″), 75.3 (C-1″), 72.8 (C-2″), 72.3, 71.5 (C-4″)*, 63.1, 62.7 (C-6″)*. *Two peaks were observed due to the presence of rotamers. HRMS-ESI (*m*/*z*): [M + H]^+^ calcd for C_21_H_21_O_9_ 417.1180, found 417.1174.

*8-(β-d-Glucopyranosyl)-5,7,4′-trihydroxyflavone* (**17**). Purified by column chromatography (DCM/MeOH 1:0 to 5:1). Isolated yield over two steps: 7%; R_f_ = 0.43 (EtOAc/EtOH 6:1). ^1^H NMR (MeOD) δ (ppm) 7.93 (d, 2H, *J*_ortho_ = 8.6 Hz, H-2′, H-6′), 6.91 (d, 2H, *J*_ortho_ = 8.8 Hz, H-3′, H-5′), 6.49 (s, 1H, H-3), 6.14 (s, 1H, H-6), 5.05 (d, 1H, *J*_1″-2″_ = 9.7 Hz, H-1″), 4.17–4.10 (m, 1H, H-2″), 3.94 (d, 1H, *J*_6″a-6″b_ = 12.0 Hz, H-6″a), 3.79 (dd, 1H, *J*_6″b-6″a_ = 12.1 Hz, *J*_6″b-5″_ = 5.5 Hz, H-6″b), 3.65 (t, 1H, *J*_4″-3″_ = *J*_4″-5″_ = 9.2 Hz, H-4″), 3.56–3.48 (m, 2H, H-3″, H-5″). ^13^C NMR (MeOD) δ (ppm) 182.1 (C-4), 168.2 (C-4′), 164.4 (C-7), 163.3 (C-2), 161.2 (C-5), 160.8 (C-8a), 129.8 (C-2′, C-6′), 124.6 (C-1′), 117.3 (C-3′, C-5′), 106.2 (C-4a), 104.5 (C-8), 102.8 (C-3), 99.3 (C-6), 82.8 (C-2″), 80.7 (C-5″), 78.5 (C-3″), 74.4 (C-1″), 72.5 (C-4″), 61.4 (C-6″). HRMS-ESI (*m*/*z*): [M + H]^+^ calcd for C_21_H_21_O_10_ 433.1129, found 433.1120.

*4′-Fluoro-8-(β-d-glucopyranosyl)-5,7-dihydroxyflavone* (**18**). Purified by preparative HPLC. Reaction yield over two steps: 45%; LCMS: RT= 0.71 min, *m*/*z* = 433.00 [M - H]^-^ (low pH method); colorless oil. ^1^H NMR (MeOD) δ (ppm) 8.12 (dd, 2H, *J*_ortho_ = 8.20 Hz, *J*_H-F_ = 5.9 Hz, H-2′, H-6′), 7.27 (t, 2H, *J*_ortho~H-F_ = 8.7 Hz, H-3′, H-5′), 6.52 (s, 1H, H-3), 6.02 (s, 1H, H-6), 5.04 (d, 1H, *J*_1″-2″_ = 9.3 Hz, H-1″), 4.11 (t, 1H, *J*_2″-1″_ = *J*_2″-3″_ = 9.2 Hz, H-2″), 3.89 (dd, 1H, *J*_6″a-6″b_ = 12.3 Hz, *J*_6″a-5″_ = 1.8 Hz, H-6″a), 3.80 (dd, 1H, *J*_6″b-6″a_ = 12.1 Hz, *J*_6″b-5″_ = 4.8 Hz, H-6″b), 3.65 (t, 1H, *J*_4″-3″_ = *J*_4″-5″_ = 9.3 Hz, H-4″), 3.51 (t, 1H, *J*_3″-2″_ = *J*_3″-4″_ = 9.0 Hz, H-3″), 3.48–3.44 (m, 1H, H-5″). ^13^C NMR (MeOD) δ (ppm) 182.5 (C-4), 167.5 (C-2), 166.0 (d, *J*_C-F_ = 251.2 Hz, C-4′), 163.3 (C-7), 161.9 (C-5), 159.1 (C-8a), 130.3 (d, *J*_C-F_ = 8.8 Hz, C-2′, C-6′), 129.9 (d, *J*_C-F_ = 3.2 Hz, C-1′), 117.0 (d, *J*_C-F_ = 22.5 Hz, C-3′, C-5′), 105.6 (C-4a), 104.8 (C-3), 104.4 (C-6), 104.1 (C-8), 82.6 (C-5″), 80.9 (C-3″), 76.0 (C-1″), 73.8 (C-2″), 72.1 (C-4″), 62.8 (C-6″). HRMS-ESI (*m*/*z*): [M + H]^+^ calcd for C_21_H_20_FO_9_ 435.1086, found 435.1083.

*8-(β-d-Glucopyranosyl)-5,7-dihydroxy-4′-(morpholin-4-yl)flavone* (**19**). Purified by preparative HPLC. Reaction yield over two steps: 74%; LCMS: RT = 0.57 min, *m*/*z* = 500.0 [M −  H]^−^ (high pH method); orange solid; m.p. = 210.5–211.4 °C; ${\left[\alpha \right]}_{D}^{20}$ = + 10 (*c* 0.5 MeOH); ^1^H NMR (MeOD) δ (ppm) 7.94, 7.84 (d, 2H, *J*_ortho_ = 8.3 Hz, H-2′ and H-6′)*, 7.01 (d, 2H, *J*_ortho_ = 8.6 Hz, H-3′ and H-5′), 6.52 (s, 1H, H-3), 6.26 (s, 1H, H-6), 5.05, 4.99 (d, 1H, *J*_1″-2″_ = 9.9 Hz, H-1″)*, 4.14 (t, 1H, *J*_2″-1″_ = *J*_2″-3″_ = 9.5 Hz, H-2″), 3.98–3.79 (m, 6H, H-6″a, H-6″b and NCH_2_C*H*_2_O), 3.70 (t, 1H, *J*_4″-3″_ = *J*_4″-5″_ = 9.6 Hz, H-4″), 3.57–3.53 (m, 1H, H-3″), 3.49–3.46 (m, 1H, H-5″), 3.32–3.20 (NC*H*_2_CH_2_O, superimposed with the MeOD peak). ^13^C NMR (MeOD) δ (ppm) 184.0 (C-4), 166.5 (C-2), 164.4 (C-7), 162.6 (C-5), 158.0 (C-8a), 155.1 (C-4′), 129.5, 129.1 (C-2′ and C-6′)*, 122.0 (C-1′), 115.3 (C-3′ and C-5′), 105.8 (C-4a), 105.2 (C-8), 103.0 (C-3), 99.4 (C-6), 82.8 (C-5″), 80.3 (C-3″), 75.3 (C-1″), 72.9 (C-2″), 72.3 (C-4″), 67.7 (NCH_2_*C*H_2_O), 63.1 (C-6″), 40.4 (N*C*H_2_CH_2_O). *Peaks were observed due to the presence of rotamers. HRMS-ESI (*m*/*z*): [M + H]^+^ calcd for C_25_H_28_NO_10_ 502.1708, found 502.1695.

*8-(β-d-Glucopyranosyl)-5,7-dihydroxy-4′-(pyrrolidin-1-yl)flavone* (**20**). Purified by preparative HPLC. Reaction yield over two steps: 80%; LCMS: RT = 0.86 min, *m*/*z* = 486.00 [M  +  H]^+^ (low pH method); orange oil. ^1^H NMR (MeOD) δ (ppm) 7.93 (d, 2H, *J*_ortho_ = 8.2 Hz, H-2′ and H-6′)*, 6.67 (d, 2H, *J*_ortho_ = 8.3 Hz, H-3′ and H-5′), 6.50 (s, 1H, H-3), 6.25 (s, 1H, H-6), 4.99 (d, 1H, *J*_1″-2″_ = 9.9 Hz, H-1″)*, 4.16 (t, 1H, *J*_2″-1″_ =*J*_2″-3″_ = 9.4 Hz, H-2″), 3.98 (d, 1H, *J*_6-a″- 6-b″_ = 11.8 Hz, H-6″a)*, 3.80 (dd, 1H, *J*_6-b″-6-a″_ = 11.9 Hz, *J*_6-b″,5″_ = 5.8 Hz, H-6″b), 3.70 (t, 1H, *J*_4″-3″_ = *J*_4″-5″_ = 9.2 Hz, H-4″), 3.56–3.46 (m, 2H, H-3″, H-5″), 3.38–3.35 (m, 4H, NC*H*_2_CH_2_), 2.07 (s, 4H, NCH_2_C*H*_2_). ^13^C NMR (MeOD) δ (ppm) 181.6 (C-4), 167.6 (C-2), 165.5 (C-7), 162.6 (C-5), 160.4 (C-8a), 156.4 (C-4′), 129.7 (C-2′ and C-6′), 121.7 (C-1′), 112.8 (C-3′ and C-5′), 105.0 (C-4a), 104.5 (C-8), 101.5 (C-3), 97.9 (C-6), 82.9 (C-5″), 80.3 (C-3″), 75.3 (C-1″), 72.9 (C-2″), 72.4 (C-4″), 63.2 (C-6″), 49.1 (N*C*H_2_CH_2_, overlapped with the MeOD peak), 26.4 (NCH_2_*C*H_2_). *Peaks were observed due to the presence of rotamers. HRMS-ESI (*m*/*z*): [M + H]^+^ calcd for C_25_H_28_NO_9_ 486.1759, found 486.1743.

*2-(Furan-2-yl)-8-(β-d-glucopyranosyl)-5,7-dihydroxy-4*H*-chromen-4-one* (**21**). Purified by preparative HPLC. Reaction yield over two steps: 56%; LCMS: RT = 0.62 min, *m*/*z* = 404.80 [M − H]^−^ (low pH method); yellowish oil. ^1^H NMR (MeOD) δ (ppm) 7.82 (d, 1H, *J*_2′-3′_ = 3.5 Hz, H-2′), 7.33 (br s, 1H, H-4′), 6.72 (dd, 1H, *J*_3′-4′_ = 1.8 Hz, H-3′), 6.53 (s, 1H, H-3), 6.28 (s, 1H, H-6), 4.96 (d, 1H, *J*_1″-2″_ = 9.4 Hz, H-1″), 4.16 (t, 1H, *J*_2″-1″_ = *J*_2″-3″_ = 9.4 Hz, H-2″), 3.89 (d, 1H, *J*_6-a″,6-b″_ = 11.6 Hz, H-6″a), 3.73 (dd, 1H, *J*_6-b″_ = *J*_5″_ = 5.1 Hz, H-6″b), 3.64 (t, 1H, *J*_4″-3″_ = *J*_4″-5″_ = 9.4 Hz, H-4″), 3.53 (t, 1H, *J*_4″-3″~4″-5″_ = 9.2 Hz, H-3″), 3.46–3.41 (m, 1H, H-5″). ^13^C NMR (MeOD) δ 183.7 (C-4), 167.2 (C-2), 164.9 (C-7), 162.9 (C-5), 157.3 (C-8a), 148.0 (C-2′), 147.3 (C-1′), 115.6 (C-4′), 113.9 (C-3′), 107.2 (C-4a), 106.0 (C-8), 103.5 (C-3), 99.6 (C-6), 82.6 (C-5″), 80.1 (C-3″), 75.0 (C-1″), 72.9 (C-2″), 72.3 (C-4″), 63.0 (C-6″). HRMS-ESI (*m*/*z*): [M + H]^+^ calcd for C_19_H_19_NO_10_ 407.0973, found 407.0965.

*8-(β-d-Glucopyranosyl)-5,7-dihydroxy-4*H*-chromen-4-one* (**22**). Compound **42** (0.195 g, 0.221 mmol, 1 eq.) was dissolved in anhydrous DCM (5 mL). The mixture was stirred at −78 °C, and BCl_3_ (1M solution in DCM, 2.21 mL, 2.21 mmol, 10 eq.) was added in a dropwise manner over 5 min. The reaction was complete after 1 h, as detected by LCMS, and was quenched with a 1:1 mixture of DCM/MeOH (40 mL). The mixture was stirred for approximately 20 min at room temperature. The solvent was evaporated under vacuum and the residue purified by column chromatography (DCM-MeOH 1:0 to 4:1). Compound **22** was obtained as a white solid; m.p. = 192.5–193.0 °C; ${\left[\alpha \right]}_{D}^{20}$ = + 16 º (*c* 0.5 MeOH). Reaction yield: 45%; LCMS: RT = 0.41 min, *m*/*z* = 338.80 [M - H]^-^ (low pH method). ^1^H NMR (MeOD) δ (ppm) 8.04 (d, 1H, *J*_cis_ = 5.8 Hz, H-2), 6.28 (s, 1H, H-6), 6.24 (d, 1H, *J*_cis_ = 5.9 Hz, H-3), 4.91 (d, 1H, *J*_1″-2″_ = 9.9 Hz, H-1″), 4.07 (t, 1H, *J*_2″-1″~2″-3″_ = 9.3 Hz, H-2″), 3.88 (dd, 1H, *J*_6-a″~6-b″_ = 12.1 Hz, *J*_6-a″~5″_ = 2.1 Hz, H-6″a), 3.71 (dd, 1H, *J*_6-b″~6-a″_ = 12.0 Hz, *J*_6-b″~5″_ = 5.4 Hz, H-6″b), 3.50–3.41 (m, 3H, H-3″, H-4″, H-5″). ^13^C NMR (MeOD) δ 183.7 (C-4), 165.0 (C-7), 163.0 (C-5), 158.0 (C-8a and C-2), 111.5 (C-3), 106.8 (C-4a), 104.9 (C-8), 100.5 (C-6), 82.6 (C-5″), 80.1 (C-3″), 75.4 (C-1″), 72.8 (C-2″), 71.8 (C-4″), 62.9 (C-6″). HRMS-ESI (*m*/*z*): [M + H]^+^ calcd for C_15_H_17_NO_9_ 341.0867, found 341.0865.

*LogD_7.4_ determination.* The in-silico prediction tool ALOGPS [4] was used to estimate the octanol-water partition coefficients (log *P*) of the compounds. Depending on these values, the compounds were classified either as hydrophilic (log *P* below zero), moderately lipophilic (log *P* between zero and one), or lipophilic (log *P* above one) compounds. For each category, two different ratios (volume of octan-1-ol to volume of buffer) were defined as experimental parameters (Table 2).

Equal amounts of phosphate buffer (0.1 M, pH 7.4) and octan-1-ol were mixed and shaken vigorously for 5 min to saturate the phases. The mixture was left until separation of the two phases, and the buffer was retrieved. Stock solutions of the test compounds were diluted with buffer to a concentration of 1 μM. For each compound, three determinations per octan-1-ol:buffer ratio were performed in different wells of a 96-well plate. The respective volumes of buffer-containing analyte (1 μM) were pipetted to the wells and covered by saturated octan-1-ol according to the chosen volume ratio. The plate was sealed with aluminum foil, shaken (1350 rpm, 25 °C, 2 h) on a Heidolph Titramax 1,000 plate-shaker (Heidolph Instruments GmbH & Co. KG, Schwabach, Germany) and centrifuged (2,000 rpm, 25 °C, 5 min, 5804 R Eppendorf centrifuge, Hamburg, Germany). The aqueous phase was transferred to a 96-well plate for analysis by liquid chromatography-mass spectrometry (LCMS, see below). Log *P* coefficients were calculated from the octan-1-ol:buffer ratio (o:b), the initial concentration of the analyte in buffer (1 μM), and the concentration of the analyte in buffer (*cB*) according to the following equation:

(1) $$\log P=\log\left(\frac{1\;\mu M-c_{B}}{c_{B}}\times \frac{1}{o:b}\right)$$

Results are presented as the mean ± SD of three independent experiments. If the mean of two independent experiments obtained for a given compound did not differ by more than 0.1 units, the results were accepted.

*Parallel artificial membrane permeability assay (PAMPA).* Effective permeability (log *P*_e_) was determined in a 96-well format with PAMPA [34]. For each compound, measurements were performed at pH 7.4 in quadruplicates. Four wells of a deep well-plate were filled with 650 μL of PRISMA HT universal buffer, adjusted to pH 7.4 by adding the requested amount of NaOH (0.5 M). Samples (150 μL) were withdrawn from each well to determine the blank spectra by UV/Vis-spectroscopy (190 to 500 nm, SpectraMax 190, Molecular Devices, Silicon Valley, CA, USA). Then the analyte, dissolved in DMSO (10 mM), was added to the remaining buffer to yield 50 μM solutions. To exclude precipitation, the optical density (OD) was measured at 650 nm, and solutions exceeding OD 0.01 were filtrated. Afterwards, samples (150 μL) were withdrawn to determine the reference spectra. Further 200 μL were transferred to each well of the donor plate of the PAMPA sandwich (pIon, P/N 110 163). The filter membranes at the bottom of the acceptor plate were infused with 5 μL of GIT-0 Lipid Solution and 200 μL of Acceptor Sink Buffer were filled into each acceptor well. The sandwich was assembled, placed in the GutBox^TM^, and left undisturbed for 16 h. Then, it was disassembled and samples (150 μL) were transferred from each donor and acceptor well to UV-plates for determination of the UV/Vis spectra. Effective permeability (log *P*e) was calculated from the compound flux deduced from the spectra, the filter area, and the initial sample concentration in the donor well with the aid of the PAMPA Explorer Software (pIon, version 3.5).

*LC-MS measurements.* Analyses were performed using a 1100/1200 Series HPLC System coupled to a 6410 Triple Quadrupole mass detector (Agilent Technologies, Inc., Santa Clara, CA, USA) equipped with electrospray ionization. The system was controlled with the Agilent MassHunter Workstation Data Acquisition software (version B.01.04). The column used was an AtlantisR T3 C18 column (2.1 × 50 mm) with a 3 μm-particle size (Waters Corp., Milford, MA, USA). The mobile phase consisted of eluent A: 10 mM ammonium acetate, pH 5.0 in 95:5, H_2_O:MeCN; and eluent B: MeCN containing 0.1% formic acid. The flow rate was maintained at 0.6 mL/min. The gradient was ramped from 95% A/5% B to 5% A/95% B over 1 min, and then held at 5% A/95% B for 0.1 min. The system was then brought back to 95% A/5% B, resulting in a total duration of 4 min. MS parameters such as fragmentor voltage, collision energy, and polarity were optimized individually for each drug, and the molecular ion was followed for each compound in the multiple reaction monitoring mode. The concentrations of the analytes were quantified by the Agilent Mass Hunter Quantitative Analysis software (version B.01.04).

*Neuroprotective assays in human neuroblastoma (SH-SY5Y) cells.* SH-SY5Y cells were grown in Dulbecco’s Modified Eagle Medium (DMEM, Gibco, Life Technologies) supplemented with 10% fetal bovine serum (FBS, Biochrom GmbH) and 1% Penicillin-Streptomycin (Gibco, Life Technologies) in a humidified incubator at 37 °C, 5% CO_2_. For the neuroprotective activity assay, undifferentiated SGSY-5Y cells were plated onto 96-well flat-bottomed microtiter plates at a density of 1 × 10^4^ cells/well in DMEM supplemented with 2% FBS and preincubated for 24 h at 37 °C, 5% CO_2_. Compounds (stored as 10 mM solutions in DMSO at −20 °C) were then added to achieve a final concentration of 50 μM and, after 30 min, cells were incubated in the presence or absence of 20 μM Aβ protein fragment 1-42 (Sigma-Aldrich, dissolved in DMSO and stored in 2 mM aliquots at −20 °C) or 100 μM of H_2_O_2_ (Sigma-Aldrich, dissolved to 10 mM in 0.9% NaCl aqueous solution immediately prior to the assay) overnight at 37 °C, 5% CO_2_. The final DMSO percentage was 0.5% for compounds in the presence of H_2_O_2_, 1% for cells incubated only with Aβ, or 1.5% for cells incubated with compounds and Aβ, thus, controls for each DMSO percentage were also run. The following morning, 20 μL of a 5 mg/mL solution of 3-(4,5-dimethylthiazol-2-yl)-2,5-diphenyltetrazolium bromide (MTT, Sigma-Aldrich) in PBS (Gibco, Life Technologies) was added to each well and the plates were further incubated for 4 h at 37 °C, followed by the addition of DMSO (200 μL) to each well in order to dissolve the resulting formazan crystals. After 2 h incubating at 37 °C, the optical density (OD) at 540 nm (with a 620 nm reference filter) was measured in an Amersham Biosciences Biotrak II Plate Reader. The percentage of MTT reduction was determined according to Equation (1). All experiments were performed in triplicate and results are presented as means ± standard error. Differences between experimental conditions were compared for statistical significance by one-way ANOVA followed by a Tukey’s post-test, an analysis carried out using GraphPad Prism Software (LA Jolla, CA, USA). Differences were considered significant when *P* < 0.05. In order to exclude direct MTT reduction, compounds were also tested in (a) the absence of cells and (b) in the absence of both cells and culture medium, using the same experimental conditions described above.

(2) $$MTT\;Reduction\;\left(\%\;of\;Control\right)=\left[\frac{OD_{sample}-OD_{medium}}{OD_{cell\;control}-OD_{medium}}\right]\;\times \;100$$

*Cell viability assay in human epithelial colorectal adenocarcinoma (Caco-2) and human liver hepatocellular (HepG2) cells.* Caco-2 and HepG2 Cells were cultured in DMEM-Dulbecco’s modified Eagle’s medium (Sigma-Aldrich) supplemented with 10% (*v*/*v*) inactivated fetal bovine serum (PAA Laboratories GmbH), 2 mM l-glutamine (Sigma-Aldrich), 100 U/mL penicillin and 100 μg/mL streptomycin (Sigma-Aldrich), in a humidified incubator at 37 °C with a 5% CO_2_ atmosphere. Cells (3 × 10^4^ cells/well) were seeded into 96-well plates and incubated at 37 °C in 5% CO_2_ atmosphere. After 24 h, compounds were added at different concentrations (0.1–100 μM) and incubated in the same conditions. DMSO controls were performed to evaluate a possible solvent cytotoxicity, while pure DMSO was used as a cytotoxic drug. After the established incubation time with compounds, MTT (5 mg/mL) in PBS (Sigma-Aldrich) was added (10 μL) to each well. After 3 h incubation at 37 °C, in 5% CO_2,_ the supernatant was removed. The formazan crystals were solubilized using ethanol/DMSO (1:1) (100 μL), and the absorbance values were determined at 570 nm on the microplate reader Victor3 from PerkinElmer Life Sciences.

## 5. Conclusions

In the present work, we have generated a library of chemically diverse flavone analogues and their *C*-glucosyl derivatives, and explored the structural requirements for neuroprotective activity and therapeutic potential against AD. 

With imposed physicochemical properties upon their design as CNS-targeted agents, all synthesized aglycones were found to have good effective permeability and adequate log*D*_7.4_ values, indicating that, as expected, they have adequate pharmacokinetic and safety profiles for administration, with the ability to cross cell membrane barriers, including gastrointestinal epithelia and the BBB. Even though not all *C*-glucosyl derivatives were able to achieve the same outcomes due to the presence of the polar sugar moiety, some of them presented a promising compromise between effective permeability and lipophilicity. From these compounds, the *p*-morpholinyl derivative **37** stood out for having fully rescued SH-SY5Y human neuroblastoma cells from H_2_O_2_- and Aβ_1-42_-induced toxicity in a screening MTT assay. Importantly, it was the only compound in which the aglycone (**8**) was also active, pointing towards aglycone specificity for the desired activity. The ability of the sugar moiety to maintain or even potentiate the activity in this compound (**8**/**19**) definitely comes across as a benefit, since according to previously published indications, the sugar may enhance the antioxidant properties of the compound as a whole, while helping to maintain Aβ_1-42_ peptides in their disaggregated state [19,20]. Furthermore, our results support the use of *C*-glucosylflavones for neuroprotective applications in detriment of the corresponding isoflavones by providing a direct comparison between two scaffolds from natural origin—compound **2** and vitexin **17**—in more than one cell-based assay. Compound **19** also exhibits a modest acetylcholinesterase inhibitory activity, thus coming across as a new multitarget lead compound for AD. This promising and non-toxic morpholinyl flavone derivative ultimately offers a relevant improvement when compared to the natural products chrysin (**1**) and **2**, which served as the inspiration for our work owing to their reported neuroprotective potential. 

## Figures and Tables

**Table 1:** Effective permeability (log *P*_e_) and partition coefficient at pH 7.4 (log *D*_7.4_) of the synthesized flavones and analogues.

Compound Nr.	Log *P*_e_^a^	Log *D*_7.4_^b^
**1**	−4.65 ± 0.09	3.6 ± 0.4
**4**	−4.66 ± 0.09	2.9 ± 0.1
**5**	−4.51 ± 0.06	>2.5
**6**	−4.48 ± 0.04	>2.5
**7**	−4.37 ± 0.12	>2.5
**8**	−4.56 ± 0.04	>2.5
**9**	−5.31 ± 0.12	n.d.^c^
**10**	−4.70 ± 0.14	3.4 ± 0.2
**11**	−4.93 ± 0.20	>2.5
**12**	−4.64 ± 0.02	n.d. ^c^
**13**	−4.76 ± 0.02	2.4 ± 0.1
**15**	Below detection limit	−0.6 ± 0.2
**16**	−8.94 ± 1.83	0.8 ± 0.3
**17**	−8.70 ± 1.50	0.1 ± 0.1
**18**	Below detection limit	−0.2 ± 0.1
**19**	−7.08 ± 0.91	1.2 ± 0.1
**20**	−6.52 ± 0.41	1.8 ± 0.2
**21**	−6.94 ± 0.50	−0.2 ± 0.1
**22**	−6.76 ± 0.11	−2.0 ± 0.2
Testosterone	−4.42 ± 0.09	-

^a^: effective permeability; ^b^: partition coefficient at pH 7.4; ^c^: not determined.

**Table 2:** Compound classification based on estimated log *P* values.

Compound Category	log *P*	Ratios (Octan-1-ol:Buffer)
hydrophilic	<0	30:140, 40:130
moderately lipophilic	0–1	70:110, 110:70
lipophilic	>1	3:180, 4:180

## Supplementary Materials

The following are available online at https://www.mdpi.com/1424-8247/12/2/98/s1, Table S1: Structure, physicochemical properties and CNS-MPO score of the flavone analogues selected for synthesis. All physicochemical properties were calculated using MOE software; Table S2: Cytotoxic activity of each compound assessed in SH-SY5Y neuroblastoma cells via an MTT cell viability assay; Experimental procedures for cholinesterase inhibition assays; Table S3: Acetylcholinesterase (AChE) and butyrylcholinesterase (BuChE) inhibitory efficacy of chrysin (**1**), 8-glucosylgenistein (**2**) and some of the synthesized flavone analogues at 100 μM; Synthetic approaches for chromones, flavones and the corresponding C-glucosyl derivatives; Scheme S1: Synthesis of selected flavones and analogues (A) via chalcone formation and (B) via flavanone formation; Scheme S2: Synthesis of (A) *C*-glucosyl flavones and analogues and (B) the 6-glucosyl-5,7-dihydroxychromen-4-one (**41**); Preparation procedures, physical and LCMS data, and NMR spectra of intermediate compounds.
