# Supplementary material for: Design and Synthesis of CNS-targeted Flavones and Analogues with Neuroprotective Potential Against H2O2- and Aβ1-42-Induced Toxicity in SH-SY5Y Human Neuroblastoma Cells
Source: Pharmaceuticals (Basel). 2019 Jun 21;12(2):98. doi: 10.3390/ph12020098 (PMC6630837; doi:10.3390/ph12020098)
Supplement: Supplementary file 1 [file pharmaceuticals-12-00098-s001.pdf]

Supplementary Materials

Table S1. Structure, physicochemical properties and CNS-MPO score of the flavone analogues selected for synthesis.[a]

Nr.	Structure	ClogP	ClogD	pKa	MW (g/mol)	TPSA (Å^2)	HBD	CNS-MPO Score
**1**	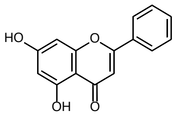	3.13	3.19	6.88	254.24	66.76	2	**4.84**
**4**	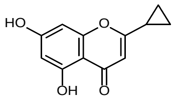	1.54	1.60	6.89	218.20	66.76	2	**5.50**
**5**	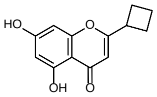	2.04	2.11	6.89	232.23	66.76	2	**5.45**
**6**	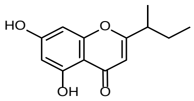	2.56	2.63	6.89	234.26	66.76	2	**5.19**
**7**	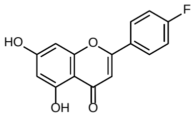	3.21	3.27	6.87	272.23	66.76	2	**4.76**
**8**	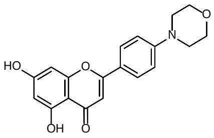	2.76	2.81	6.93	339.34	79.23	2	**5.10**
**9**	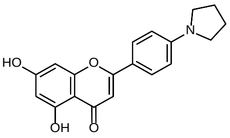	3.23	3.29	6.93	323.34	70.00	2	**4.74**
**10**	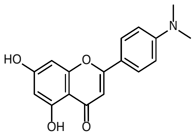	3.25	3.30	6.93	297.31	70.00	2	**4.73**
**11**	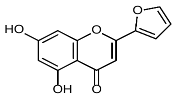	2.62	2.68	6.88	244.20	79.90	2	**5.16**
**12**	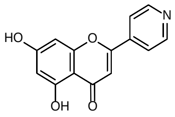	2.42	2.47	6.87	255.22	79.65	2	**5.30**
**13**	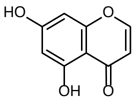	1.32	1.40	6.88	178.14	66.76	2	**5.50**

[a] All physicochemical properties were calculated using MOE software.

Table S2. Cytotoxic activity of each compound assessed in SH-SY5Y neuroblastoma cells via an MTT cell viability assay.[a]

Compound Nr.	MTT reduction (%control)	*p*-value *vs.* untreated cell control (unpaired t-test)
**1**	96.2 ± 26.9%	0.7335
**2**	142.4 ± 6.3%	0.2862
**4**	140.7 ± 12.0%	0.3394
**5**	184.6 ± 30.2%	0.1239
**6**	109.9 ± 18.7%	0.8116
**7**	109.4 ± 11.4%	0.6924
**8**	57.6 ± 25.5%	0.2810
**9**	163.6% ± 72.6%	0.2084
**10**	191.5 ± 22.9%	0.1265
**11**	129.7 ± 9.06%	0.4290
**12**	233.7 ± 15.7%	0.0423
**13**	71.2 ± 48.1%	0.5841
**15**	120.3 ± 24.3%	0.3206
**16**	142.0 ± 38.5%	0.4492
**17**	85.0 ± 38.5%	0.4492
**18**	136.7 ±12.9%	0.3821
**19**	138.5 ± 3.6%	0.2579
**20**	219.9 ± 16.9%	0.0593
**21**	131.5 ± 11.0%	0.3308
**22**	149.2 ± 21.6%	0.0683

[a] Cells were incubated for 24 h at 37 ºC, in the presence (50 µM) or absence of each compound. The tests were performed in triplicate with a final concentration of 0.5% DMSO. Differences were considered to be statistically significant when $p < 0.05$ *vs.* untreated cell controls, assessed by an unpaired t-test.

Experimental procedures for cholinesterase inhibition assays. For cholinesterase inhibition tests (acetylcholinesterase (AChE, *electrophorus electricus*) and butyrylcholinesterase (BuChE, equine serum), the Ellman's cholorimetric assay[1] was followed, with minor modifications. DMSO was kept within 1.25% cuvette concentration. The chromogenic agent DTNB [5,5'-dithiobis(2-nitrobenzoic acid)] was fixed at 0.975 mM concentration; 0.1 M phosphate buffer (pH 8.0) was employed, T = 25 ºC, and the reaction was monitored for 125 s. For determining the percentage of inhibition, the substrate concentration (acetylthiocholine iodide for AChE; *S*-butyrylthiocholine iodide for BuChE) was fixed at 29 µM for AChE and at 18.2 µM concentration for BuChE.

Lineweaver-Burk plot (or double reciprocal plot, 1/V *vs.* 1/[S]) was used for estimating both, the mode of inhibition and the inhibition constants (K_i's) for both, glycosidases and cholinesterases, using the following equations:

Competitive inhibition (inhibitor only bonds the free enzyme):

$$K_{ia} = \frac{[I]}{\frac{K_{M\,app}}{K_M} - 1}$$

Mixed inhibition (inhibitor binds both, the free and the complexed enzyme):

$$K_{M\,app} = K_M \frac{1 + \frac{[I]}{K_{ia}}}{1 + \frac{[I]}{K_{ib}}}$$

$$V_{\max app} = \frac{V_{max}}{1 + \frac{[I]}{K_{ib}}}$$

Uncompetitive (the inhibitors only binds the complexed enzyme):

$$K_{M\ app} = \frac{K_M}{1 + \frac{[I]}{K_{ib}}}$$

$$V_{\max app} = \frac{V_{max}}{1 + \frac{[I]}{K_{ib}}}$$

Non-competitive (inhibitor binds both the free enzyme and the complexed enzyme with equal affinity):

$$K_{M\,app} = K_M$$

$$V_{\max app} = \frac{V_{max}}{1 + \frac{[I]}{K_i}}$$

Table S3. Acetylcholinesterase (AChE) and butyrylcholinesterase (BuChE) inhibitory efficacy of chrysin (**1**), 8-glucosylgenistein (**2**) and some of the synthesized flavone analogues at 100 µM.

Compound No.	AChE	BuChE
**1**	18%	**64%***
**2**	26%	n.i.[a]
**4**	24%	30%
**6**	n.i.[a]	**46%****
**7**	n.i.[a]	23%
**9**	27%	32%
**16**	20%	n.i.[a]
**19**	18%	n.i.[a]
**22**	n.i.[a]	19%
**42**	10%	15%

*Non-competitive inhibition, $K_{ia} = K_{ib} = 32 \pm 4$µM; **Mixed inhibition, $K_{ia} = 44 \pm 17$ µM, $K_{ib} = 36 \pm 8$ µM; [a]No inhibition

Synthetic approaches for chromones, flavones and the corresponding C-glucosyl derivatives.

Chromones and flavones were prepared starting from MOM-diprotected acetophenone 14 (**Scheme S1**),[2] which base-catalysed Claisen-Schmidt aldol condensation reaction with the commercially available aldehydes generated chalcone type intermediates **23-30** in very good reaction yields (**Scheme S1A**). Interestingly, the isomerization acyclic/cyclic product, analogue to a chalcone/flavanone equilibrium, was detected by LCMS for compounds **24** and **30**, and the chroman-4-one **31** was the single product isolated, by reaction of **3** with cyclobutylcarboxaldehyde (**Scheme S1B)**. Subsequently, chalcones and flavanones were submitted to iodine-promoted oxidation in pyridine, followed by *p*-TsOH catalyzed deprotection to give compounds **4, 5,** and **6-12** in moderate to excellent reaction yields (**Schemes 1A** and 1**B**).

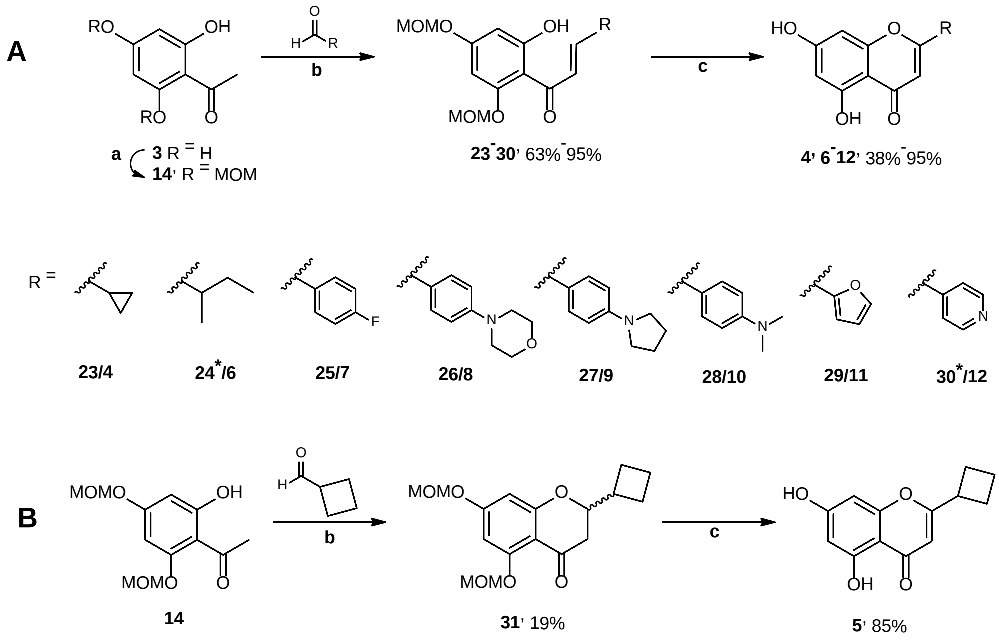

Scheme S1. Synthesis of selected flavones and analogues (**A**) via chalcone formation and (**B**) via flavanone formation. Reagents and conditions: a) Acetophloroglucinol, acetone, MOMCl, reflux, 2 h;[2] b) 1,4-dioxane, aq. NaOH 50% (w/v), reflux, 2-24 h; c) (1) pyridine, I_2, reflux, 24-48 h; (2) *p*-TsOH, EtOH, reflux, 3-24 h. Reaction yields were determined by LCMS. *Compounds not isolated.

C-glucosylchromones and flavones were synthesized starting by acetophenone C-glucosylation and selective benzylation prior to the aldol condensation step[3] (Scheme S2), which afforded the intermediate chalcones. Iodine-promoted cyclization and debenzylation with BCl_3 at low temperature gave the target glucosylchromones and glucosylflavones **15-21**. Notably, some glucosylflavones could not be obtained, either due to the high reactivity of the intermediates, or to an extreme hydrophilic character of the final product, as in the case of the 2-(pyridin-4-yl)chromone, which made purification virtually unfeasible even when using reverse phase column purification techniques such as HPLC.

The formation of 6-glucosyl-5,7-dihydroxychromen-4-one (**22**) required a different methodology as that described for its analogues. For this task, we applied the same protocol used for generating its aglycone: compound **34** reacted with sodium hydride in ethyl formate at 0 ºC to give an intermediate that was subsequently dehydrated in acid medium, under reflux, affording compound **43** in 84% yield. Further deprotection with BCl_3 in dichloromethane at low temperature gave compound **22** in good yield (**Scheme S2B).**

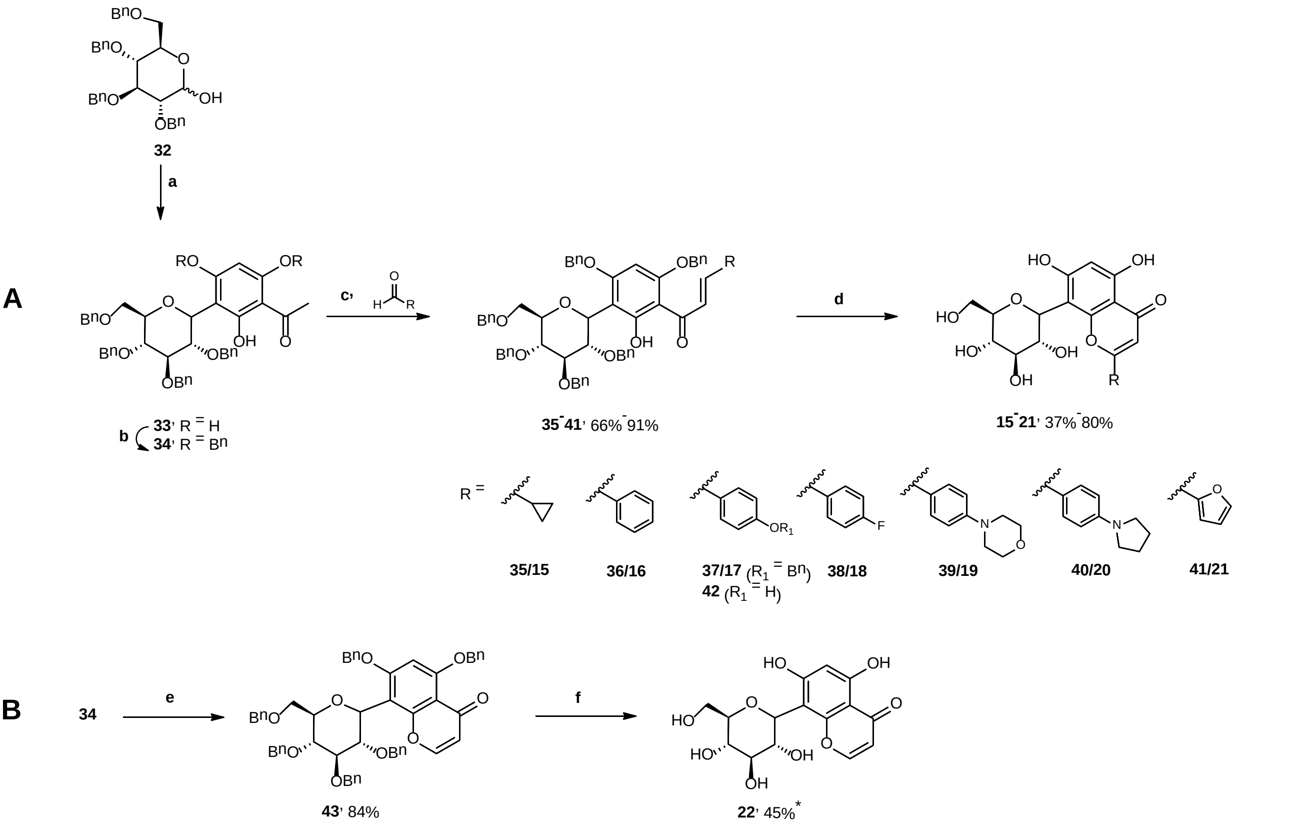

Scheme S2. Synthesis of (**A**) *C*-glucosyl flavones and analogues and (**B**) the 6-glucosyl-5,7-dihydroxychromen-4-one (**41**). Reagents and conditions: a) TMSOTf, drierite, ACN, DCM, compound 3, -78 °C → rt 6 h;[2] b) BnBr, DMF, 0 °C, then r.t., 2.5 h;[3] c)1,4-dioxane, aq. NaOH 50% (w/v), reflux, 18-24 h; d) (1) pyridine, I_2, reflux 24-48 h; (2) BCl_3, DCM, -78 °C 2-3 h; e) ethyl formate, NaH, 0 °C, 1 h, (2) MeOH, (3) conc. HCl, reflux, 18 h.; f) BCl_3, DCM, -78 °C 1 h. *Reaction yields determined by LCMS.

Preparation procedures, physical and LCMS data, and NMR spectra of intermediate compounds *(solvent used to run NMR spectra is indicated)*

For NMR characterization of chalcones, protons and carbons in ring A (aromatic ring attached to the carbonyl group) are assigned as H′, C′; in ring B (aromatic ring attached to the propenone double bond) as H″, C″; and those belonging to the glucosyl moiety as H‴, C‴, while propenone atoms are labeled from 1-3, to facilitate the description of compound chemical shifts.

General procedure for the synthesis of non-glycosylated MOM-protected chalcones and flavanones. Compound **14** (synthesized by the methodology previously described by our group)[2] was dissolved in 1,4-dioxane (0.796 mmol in 2.3 mL) and the appropriate aldehyde (1.592 mmol, 2.0 eq.) was added. The mixture was stirred until fully homogenized. Then, an aqueous solution of NaOH 50% (w/v, 2.3 mL) was slowly added and the mixture was stirred under reflux for 2 h – 24 h. All reactions were followed by LCMS; once the starting material was fully consumed, the reaction was quenched using HCl 2M, washed with brine and extracted with EtOAc (3 × 10 mL). The organic layers were combined, dried over $MgSO_4$, filtered and concentrated under vacuum. The residue was purified using the most adequate purification method(s) to afford compounds **23-30** and **19-20**.

(2*E*)-1-[2,4-Bis(methoxymethoxy)-6-hydroxyphenyl]-3-(cyclopropyl)prop-2-en-1-one (**23**). Purified by preparative HPLC. Reaction yield: 63%; LCMS: RT = 1.19 min, *m*/*z* = 309.00 $[M+H]^+$ (high pH method); yellow oil. ^1H NMR $[(CD_3)_2CO]$ δ (ppm) 13.88 (s, 1H, OH-6′), 7.49, 7.45 (part AX of olefinic ABX system, 1H, $J_{A\text{-}B}$ = 14.9 Hz, $J_{2\text{-}1''}$ = 4.1 Hz, H-2), 6.65-6.58 (part BX of olefinic ABX system, 1H, $J_{B\text{-}A}$ = 14.8 Hz, $J_{3\text{-}1''}$ = 10.4 Hz, H-3), 6.31 (br s, 1H, H-3′), 6.22 (d, 1H, J_{meta} = 2.2 Hz, H-5′), 5.37 (s, 2H, OCH_2O), 5.25 (s, 2H, OCH_2O), 3.54 (s, 3H, OCH_3), 3.45 (s, 3H, OCH_3), 1.75 (ddt, 1H, $J_{1''\text{-}3}$ = 12.4 Hz, $J_{1''\text{-}2''}$ = $J_{1''\text{-}3''}$ = 8.4 Hz, $J_{1''\text{-}2}$ = 4.1 Hz, H-1′′), 1.03-0.99 (m, 2H, H-2′′a and H-3′′a), 0.76-0.74 (m, 2H, H-2′′b and H-3′′b). ^{13}C NMR $[(CD_3)_2CO]$ δ (ppm) 193.3 (C-1), 167.7 (C-6′), 164.4 (C-2′), 161.1 (C-4′), 154.7 (C-3), 128.4 (C-2),

107.5 (C-1′), 98.0 (C-5′), 96.1 (OCH_2O), 95.8 (C-3′), 95.1 (OCH_2O), 57.2 (OCH_3), 56.7 (OCH_3), 16.1 (C-1′′), 9.7 (C-2′′ and C-3′′).

LCMS

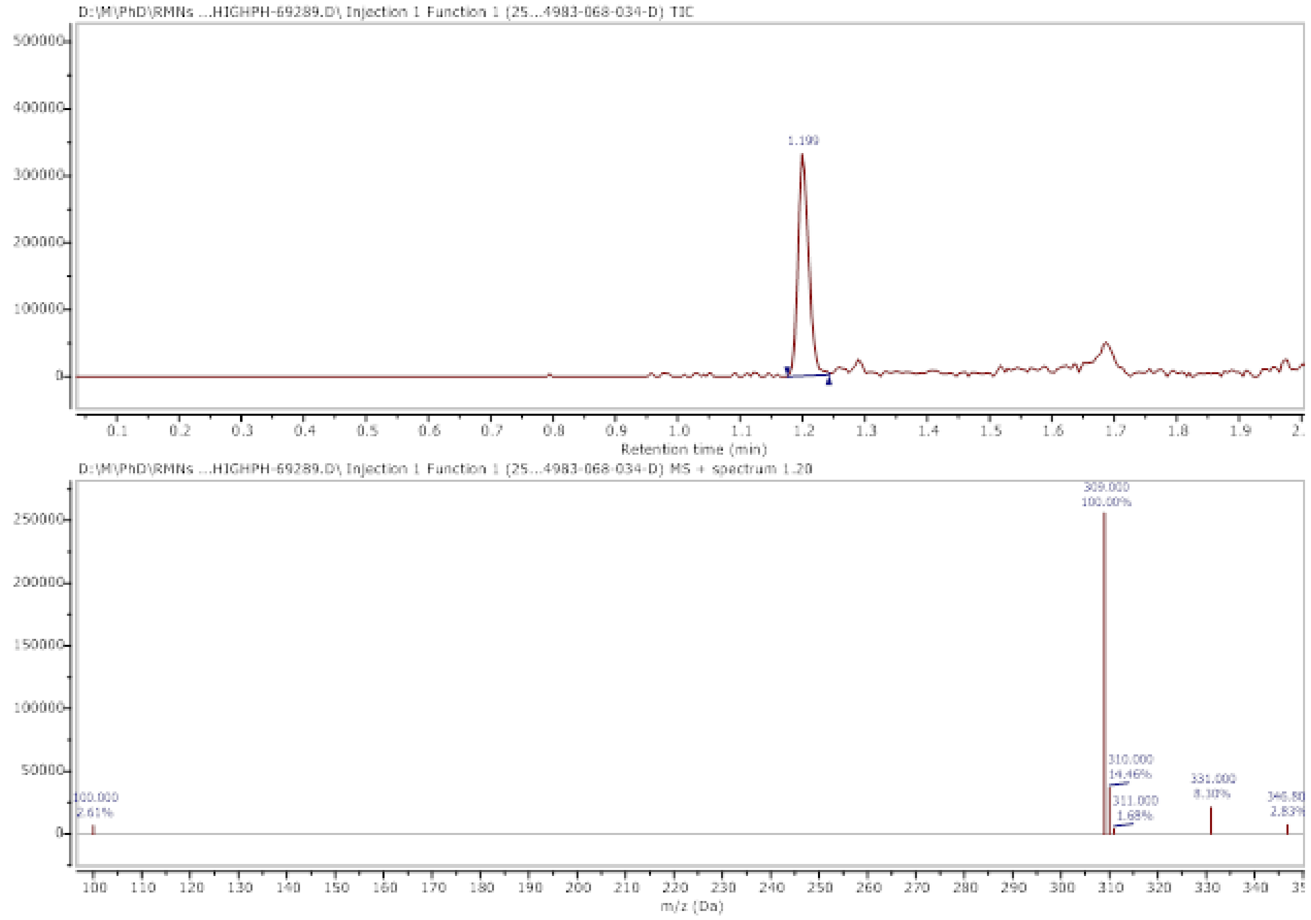

^{1}H NMR – Acetone-d_6

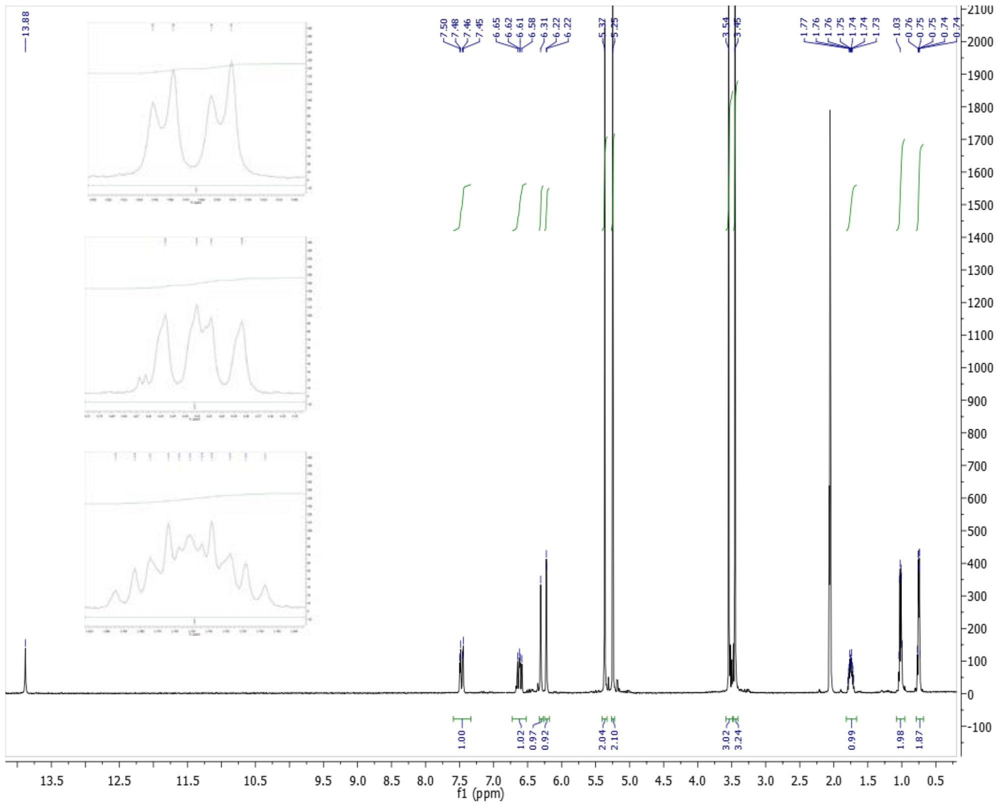

^{13}C NMR – Acetone-d_6

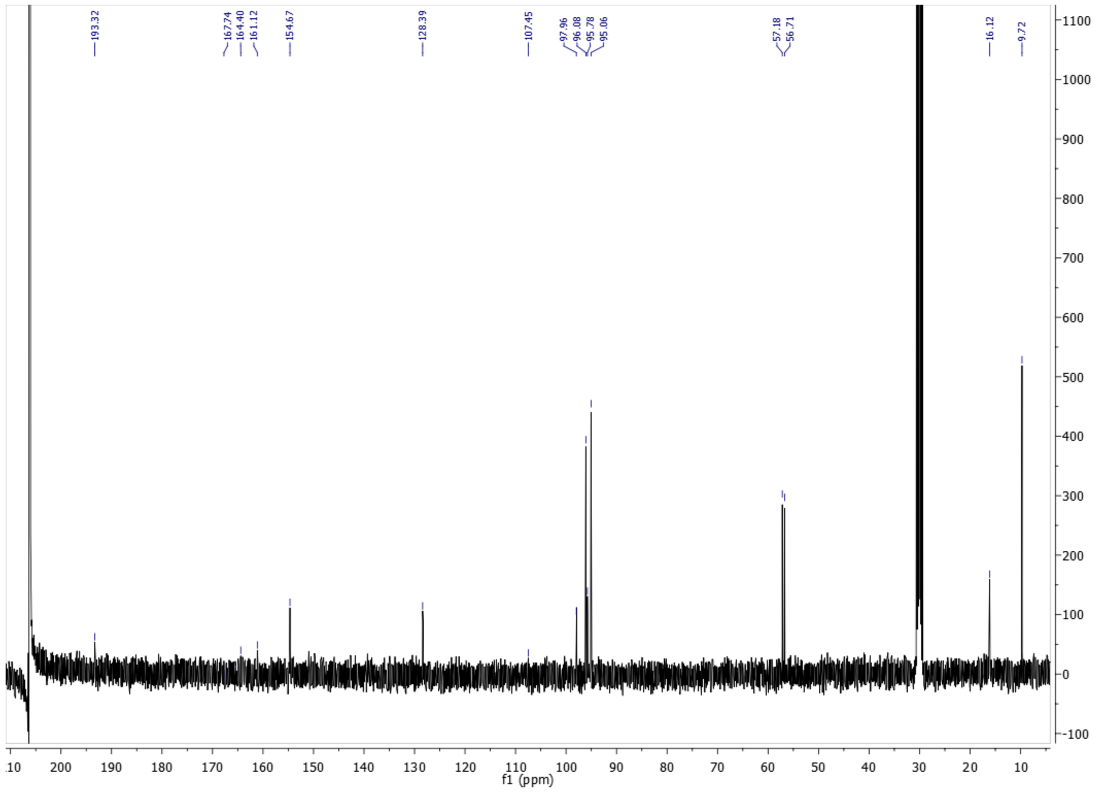

(2*E*)-1-[2,4-Bis(methoxymethoxy)-6-hydroxyphenyl]-3-(1-methylpropyl)prop-2-en-1-one (**24**). This compound was the major product of the aldol condensation reaction that afforded it, as confirmed by LCMS. However, during quenching with acid or during the work-up it was converted into an equilibrium between itself [LCMS: RT = 1.38 min, m/z = 325.0 $[M + H]^+$ and m/z = 347.0 $[M + Na]^+$ (high pH method), 38%] and the corresponding flavanone [LCMS: r.t. = 1.19 min, m/z = 325.3 $[M + H]^+$ (high pH method), 40%]. The mixture was used in the subsequent reaction without further characterization or purification.

LCMS (Mixture used in the subsequent reaction without further purification)

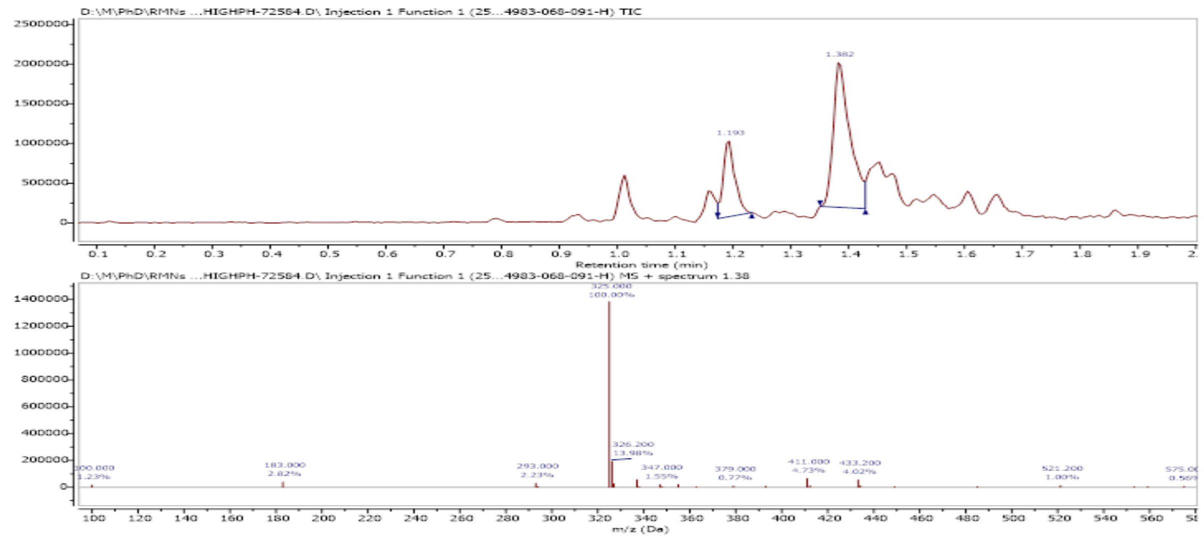

(2*E*)-1-[2,4-Bis(methoxymethoxy)-6-hydroxyphenyl]-3-(4-fluorophenyl)prop-2-en-1-one (**25**). Purified by preparative HPLC. Reaction yield: 64%; LCMS: RT = 1.33 min, *m*/*z* = 363.0 $[M + H]^+$ (high pH method); yellow oil. ^1H NMR $[(CD_3)_2CO]$ δ (ppm) 13.76 (s, 1H, OH-6′), 8.03, 7.99 (part A of olefinic AB system, 1H, $J_{A\text{-}B}$ = 15.6 Hz, H-2), 7.85-7.76 (m, part B of olefinic AB system, 3H, H-3, H-2′′, H-6′′), 7.25 (t, 2H $J_{ortho\text{-}3''\text{-}F\text{-}5''\text{-}F}$ = 8.9 Hz, H-3′′, H-5′′), 6.35 (d, 1H, J_{meta} = 2.5 Hz, H-3′), 6.27 (d, 1H, J_{meta} = 1.8 Hz, H-5′), 5.44 (s, 2H, OC*H*2O), 5.28 (s, 2H, OC*H*2O), 3.56 (s, 3H, OC*H*3), 3.47 (s, 3H, OC*H*3). ^{13}C NMR $[(CD_3)_2CO]$ δ (ppm) 193.8 (C-1), 168.3 (C-6′), 164.9 (d, $J_{C\text{-}F}$ = 249.5 Hz, C-4′′), 164.9 (C-2′), 161.3 (C-4′), 142.1 (C-3), 133.1 (C-1′′), 131.7 (d, $J_{C\text{-}F}$ = 8.2 Hz, C-2′′, C-6′′), 117.0 (d, $J_{C\text{-}F}$ = 22.2 Hz, C-3′′, C-5′′), 107.9 (C-1′), 98.0 (C-5′), 96.4 (OCH_2O), 95.9 (C-3′), 95.1 (OCH_2O).

LCMS

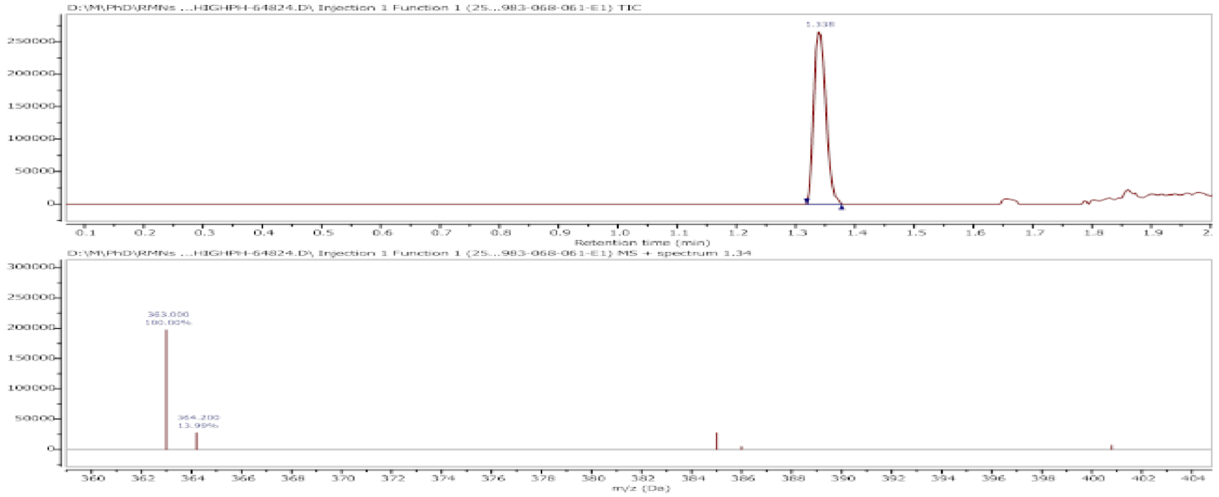

^1H NMR – Acetone-d_6

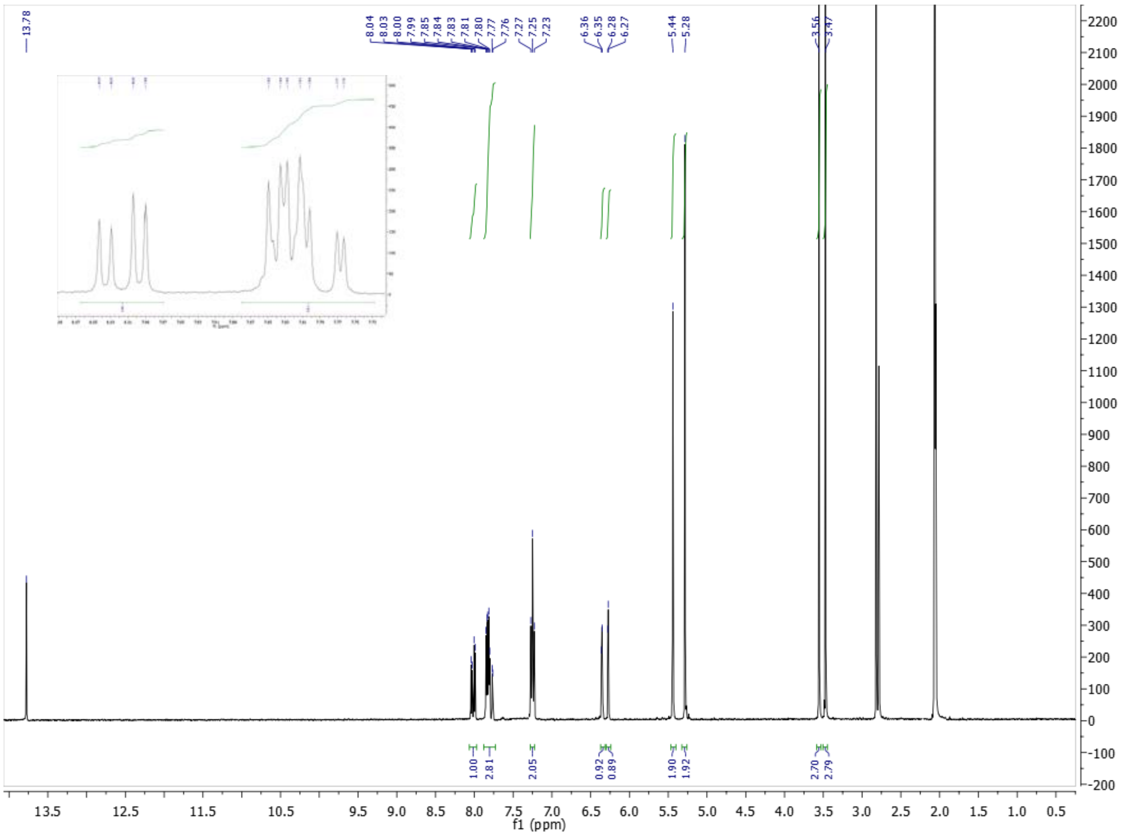

^{13}C NMR – Acetone-d_6

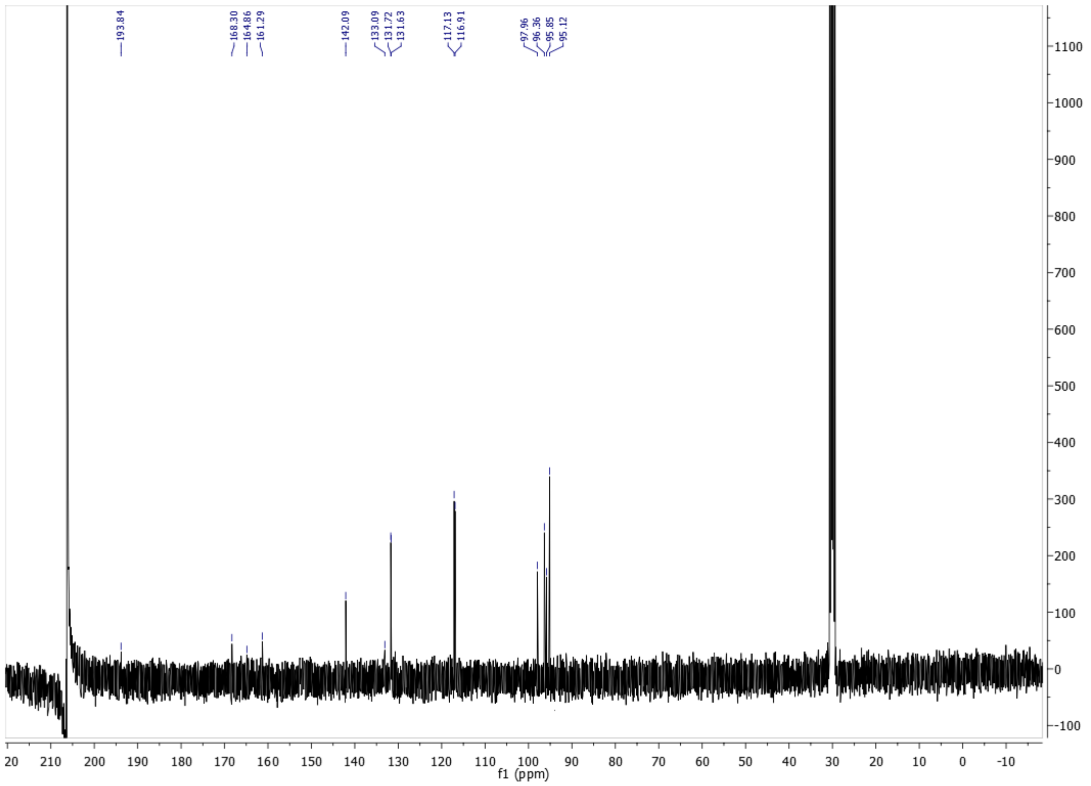

HMBC

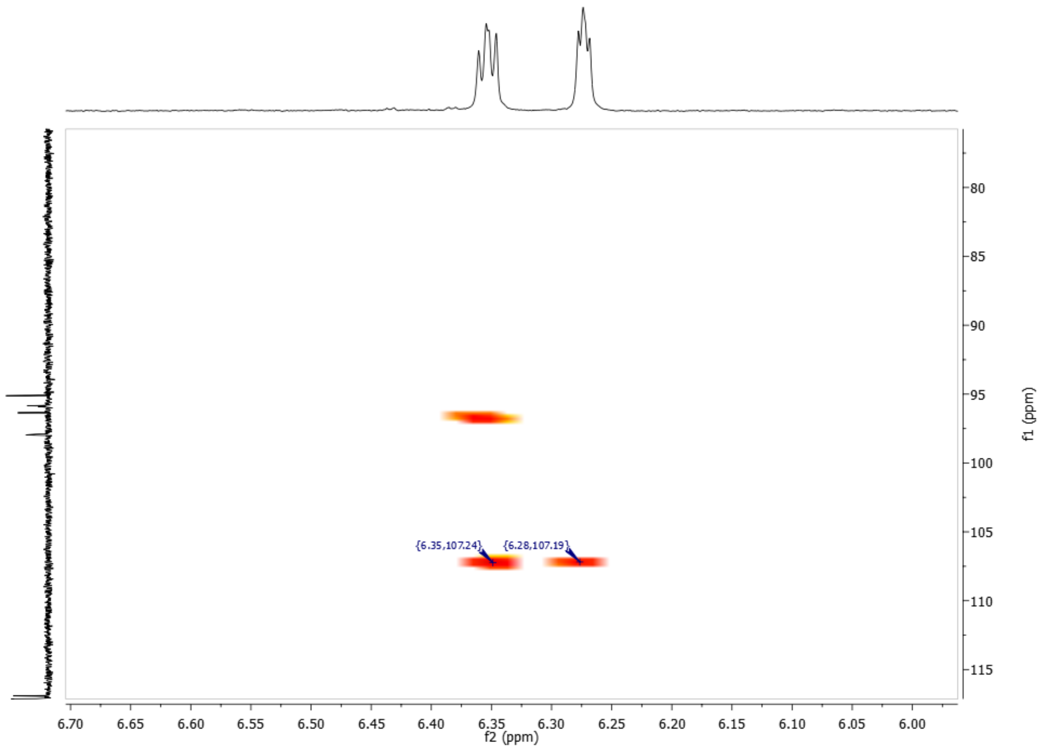

(2*E*)-1-[2,4-Bis(methoxymethoxy)-6-hydroxyphenyl]-3-[4-(morpholin-4-yl)phenyl]prop-2-en-1-one (**26**). Purified by preparative HPLC. Reaction yield: 89%; LCMS: RT = 1.24 min, *m*/*z* = 430.0 $[M + H]^+$ (high pH method); orange solid; m.p. = 134.4 – 135.2 °C. ^1H NMR $[(CD_3)_2CO]$ δ (ppm) 14.08 (s, 1H, OH-6′), 7.94, 7.90 (part AX of olefinic ABX system, 1H, $J_{A\text{-}B}$ = 15.4 Hz, , $J_{2\text{-}2''}$ = 4.0 Hz, H-2), 7.80, 7.76 (part BX of olefinic AB system, 1H, $J_{B\text{-}A}$ = 15.4 Hz, $J_{3\text{-}2''}$ = 4.0 Hz, H-2), H-3), 7.64 (d, 2H, J_{ortho} = 8.8 Hz, H-2′′, H-6′′), 7.02 (d, 2H, H-3′′, H-5′′), 6.33 (d, 1H, J_{meta} = 2.4 Hz, H-3′), 6.25 (d, 1H, J_{meta} = 2.0 Hz, H-5′), 5.42 (s, 2H, OC*H*2O), 5.26 (s, 2H, OC*H*2O), 3.81-3.78 (m, 4H, NC*H*2CH2O), 3.56 (s, 3H, OC*H*3), 3.46 (s, 3H, OC*H*3), 3.31-3.29 (m, 4H, NCH2C*H*2O). ^{13}C NMR $[(CD_3)_2CO]$ δ (ppm) 191.7 (C-1), 168.3 (C-6′), 164.3 (C-2′), 161.1 (C-4′), 154.2 (C-4′′), 144.5 (C-3), 131.2 (C-2′′, C-6′′), 126.8 (C-1′′), 124.4 (C-2), 115.6 (C-3′′, C-5′′), 108.2 (C-1′), 98.0 (C-5′), 96.4 (OCH2O), 95.8 (C-3′), 95.1 (OCH2O), 67.4 (NCH2CH2O), 57.3 (OCH3), 56.7 (OCH3), 48.8 (NCH2CH2O).

LCMS

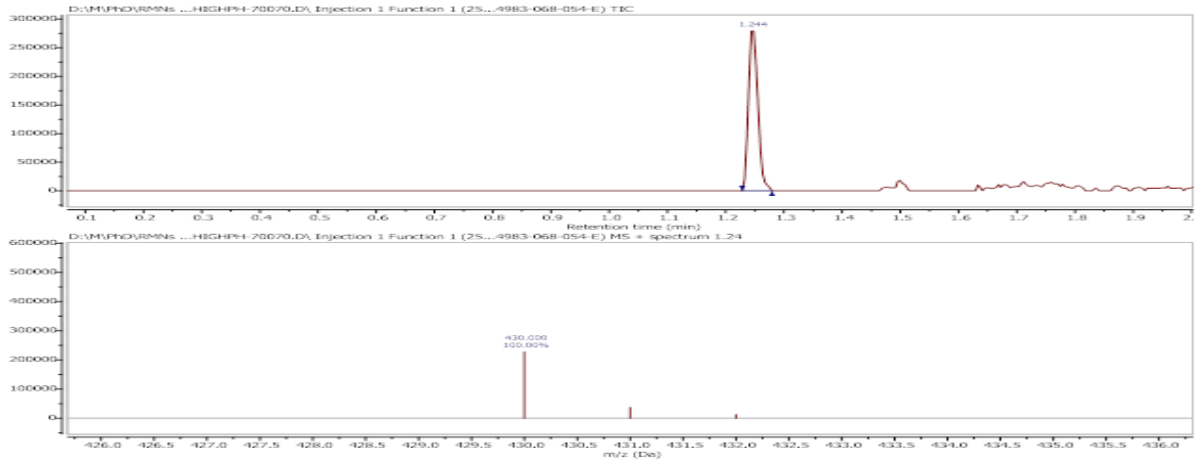

^1H NMR – Acetone-d_6

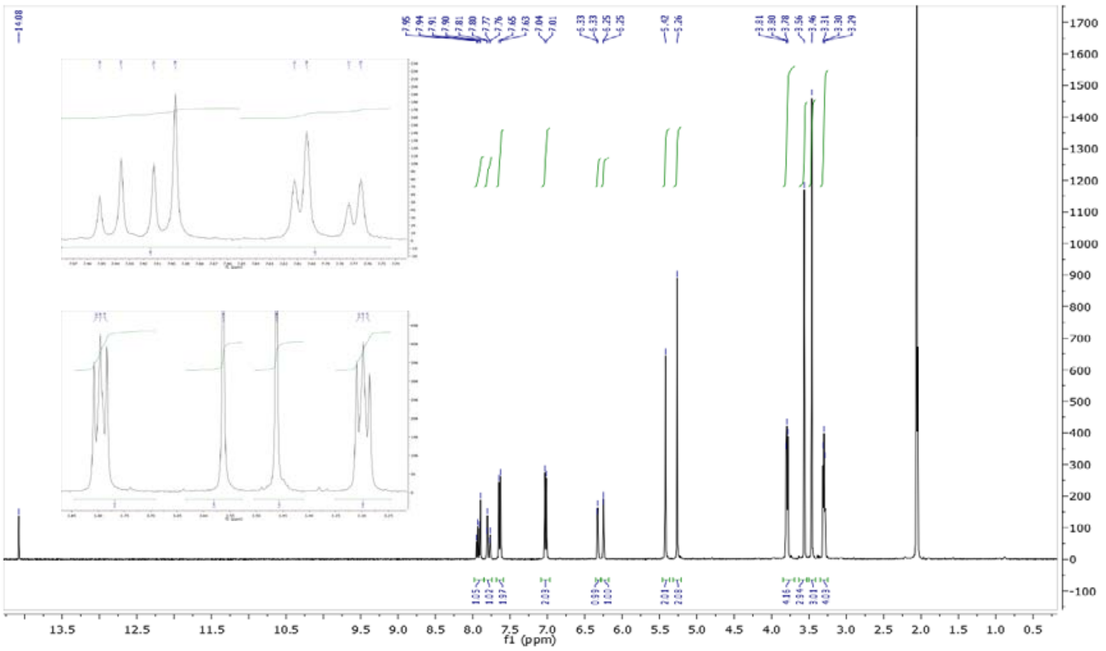

^{13}C NMR – Acetone-d_6

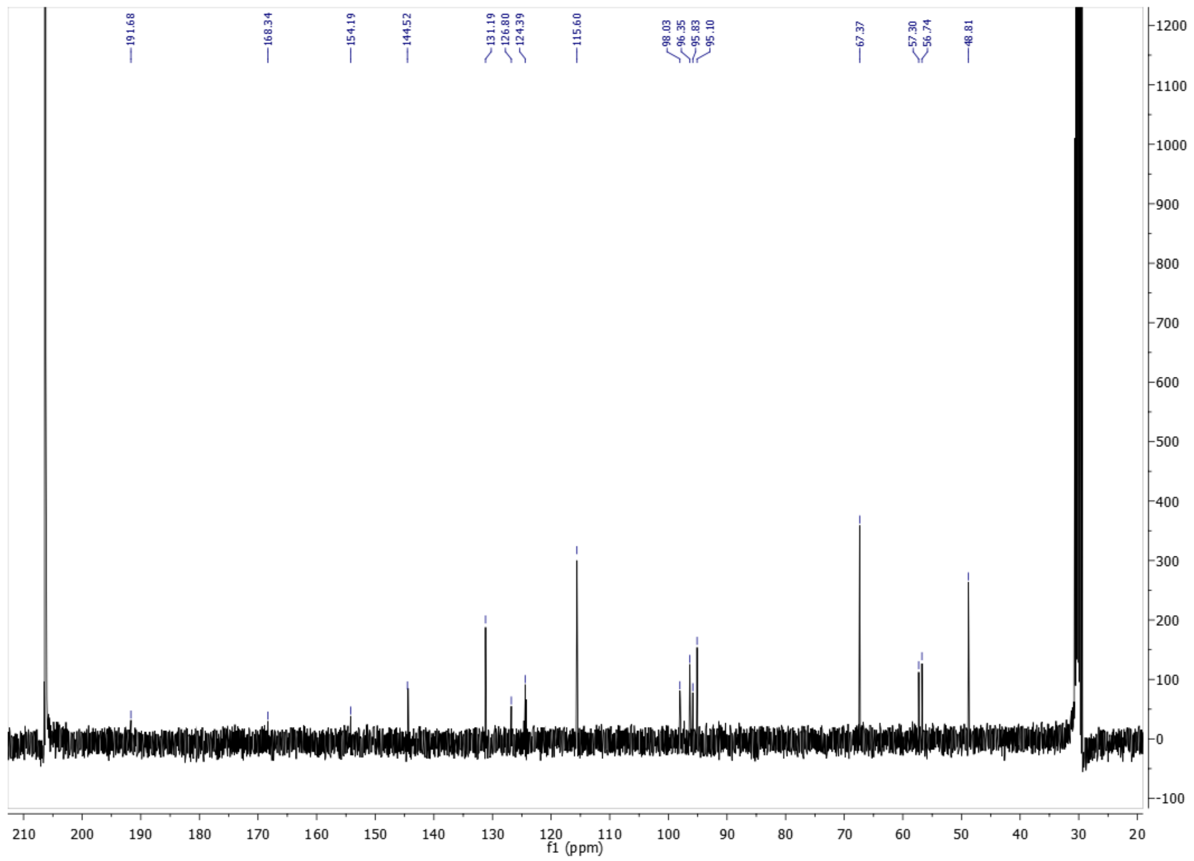

HMBC

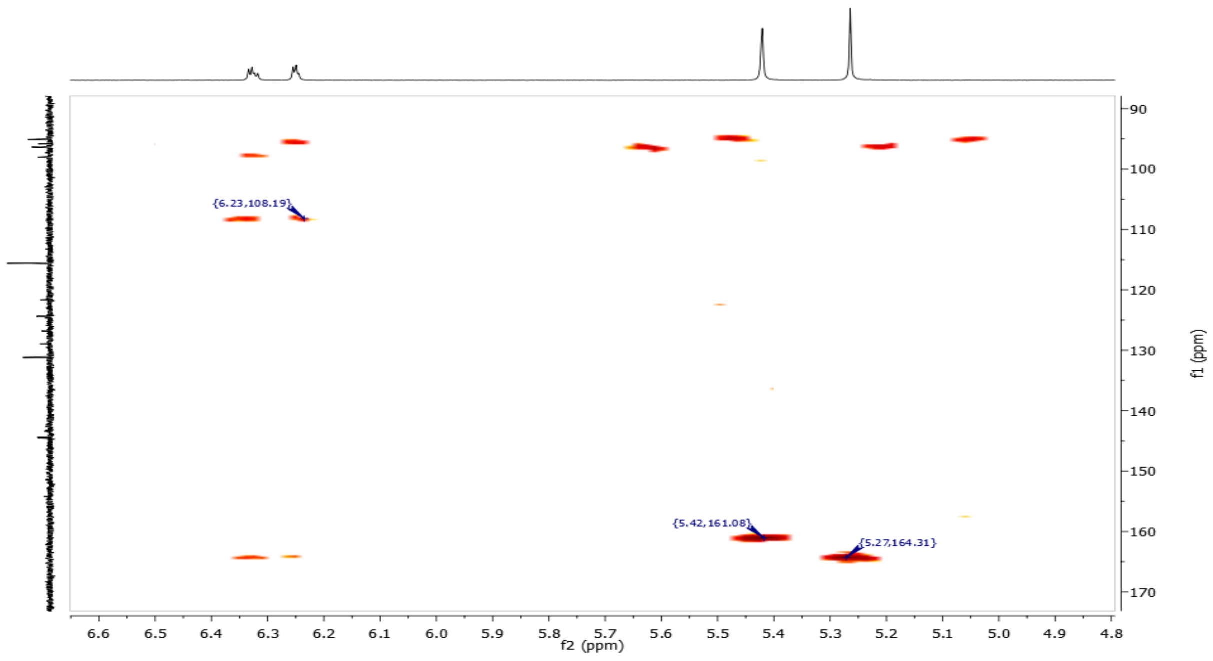

(2*E*)-1-[2,4-Bis(methoxymethoxy)-6-hydroxyphenyl]-3-[4-(pyrrolidin-1-yl)phenyl]prop-2-en-1-one (**27**). Purified by column chromatography (*iso*-hexane/THF 1:0 → 1:1). Reaction yield: 93%; LCMS: RT = 1.46 min, m/z = 414.2 $[M + H]^+$ (high pH method); red solid; m.p. = 137.7 – 138.4 °C. ^{1}H NMR [$(CD_3)_2CO$] δ (ppm) 14.28 (s, 1H, OH-6′), 7.89-7.79 (olefinic AB system, 2H, $J_{A\text{-}B}$ = 15.4 Hz, H-2, H-3), 7.58 (d, 2H, J_{ortho} = 8.7 Hz, H-2′′, H-6′′), 6.62 (d, 2H, H-3′′, H-5′′), 6.30 (d, 1H, J_{meta} = 2.4 Hz, H-3′), 6.25 (d, 1H, J_{meta} = 2.2 Hz, H-5′), 5.41 (s, 2H, OCH_2O), 5.25 (s, 2H, OCH_2O), 3.56 (s, 3H, OCH_3), 3.45 (s, 3H, OCH_3), 3.40-3.36 (m, 4H, NCH_2CH_2), 2.08-2.04 (NCH_2CH_2, overlapped with acetone-d_6 peak). ^{13}C NMR [$(CD_3)_2CO$] δ (ppm) 193.4 (C-1), 168.3 (C-6′), 164.0 (C-2′), 161.1 (C-4′), 151.2 (C-4′′), 145.8 (C-3), 131.7 (C-2′′, C-6′′), 123.4 (C-1′′), 121.7 (C-2), 112.9 (C-3′′, C-5′′), 108.9 (C-1′), 98.0 (C-5′), 96.3. (OCH_2O), 95.7 (C-3′), 95.0 (OCH_2O), 57.2 (OCH_3), 56.7 (OCH_3), 48.4 (NCH_2CH_2), 26.2 (NCH_2CH_2).

LCMS

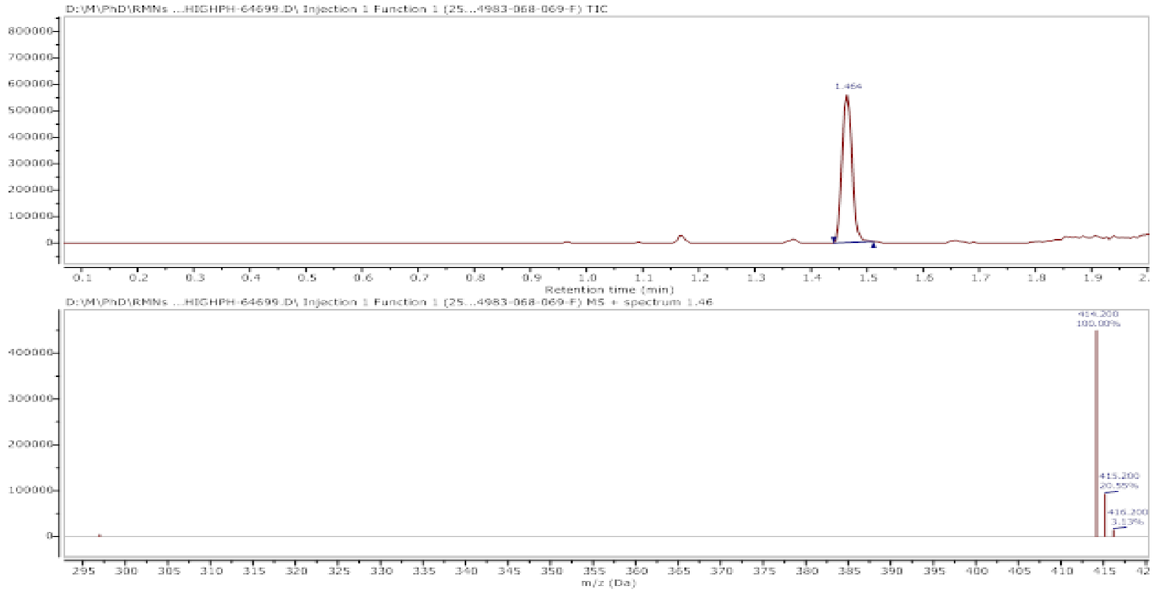

^{1}H NMR – Acetone-d_6

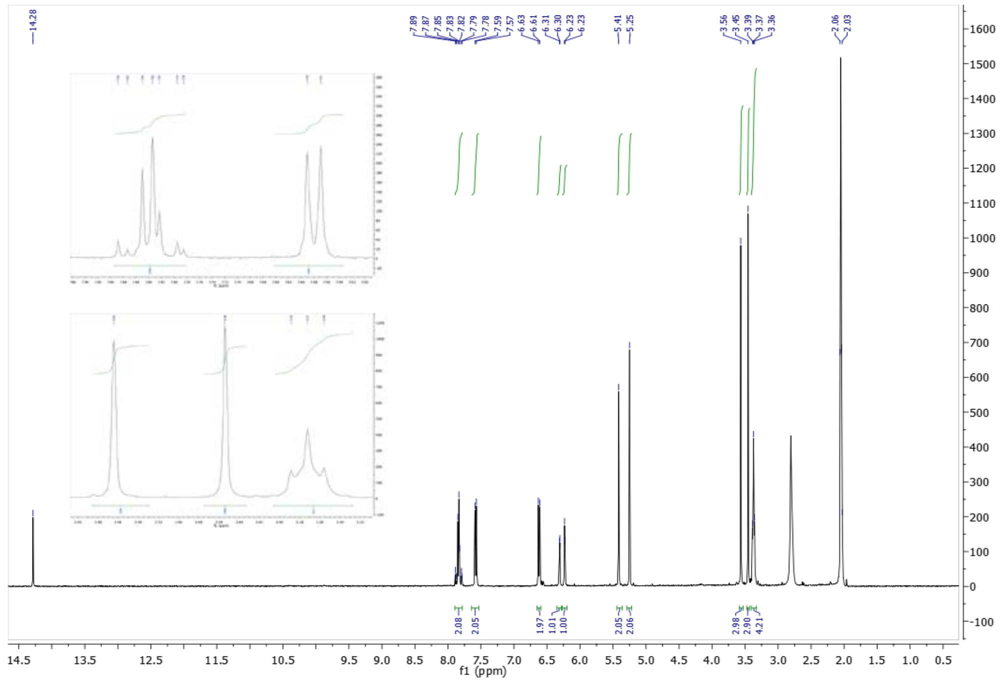

^{13}C NMR – Acetone-d_6

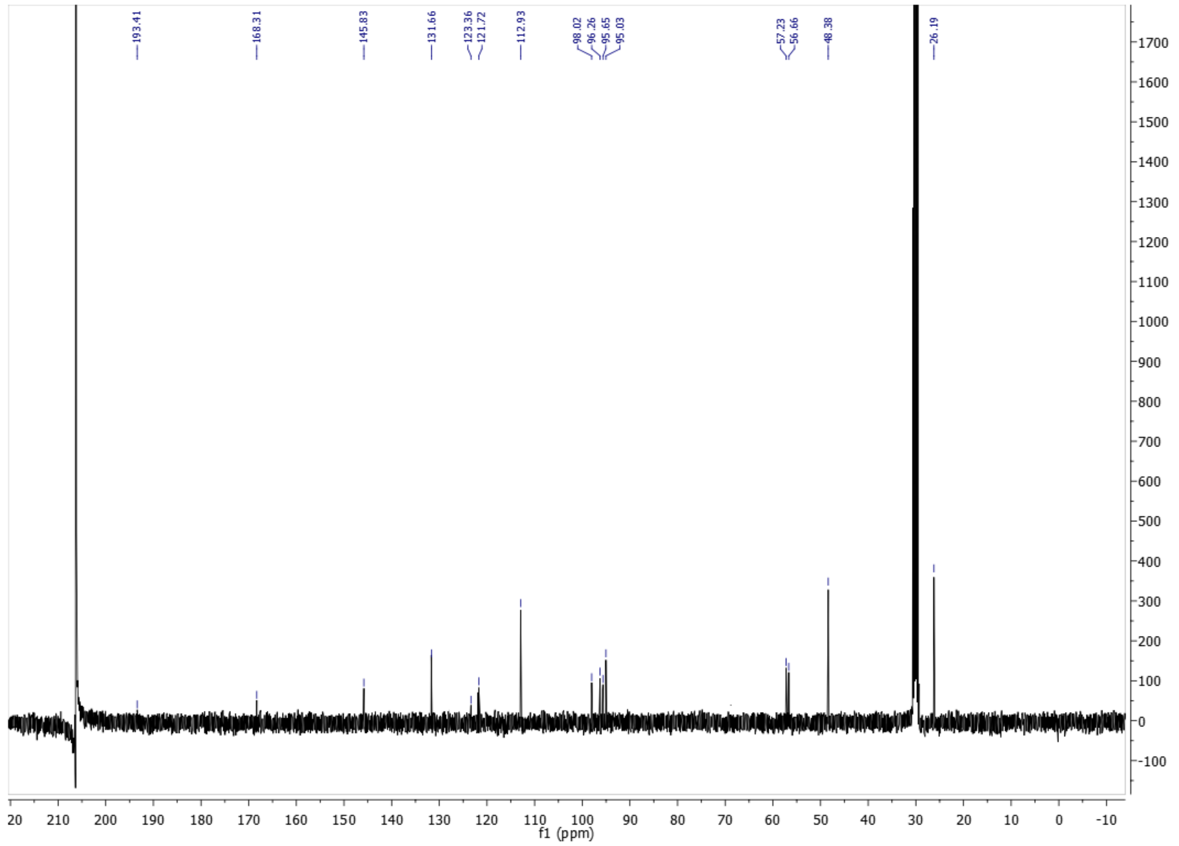

HMBC

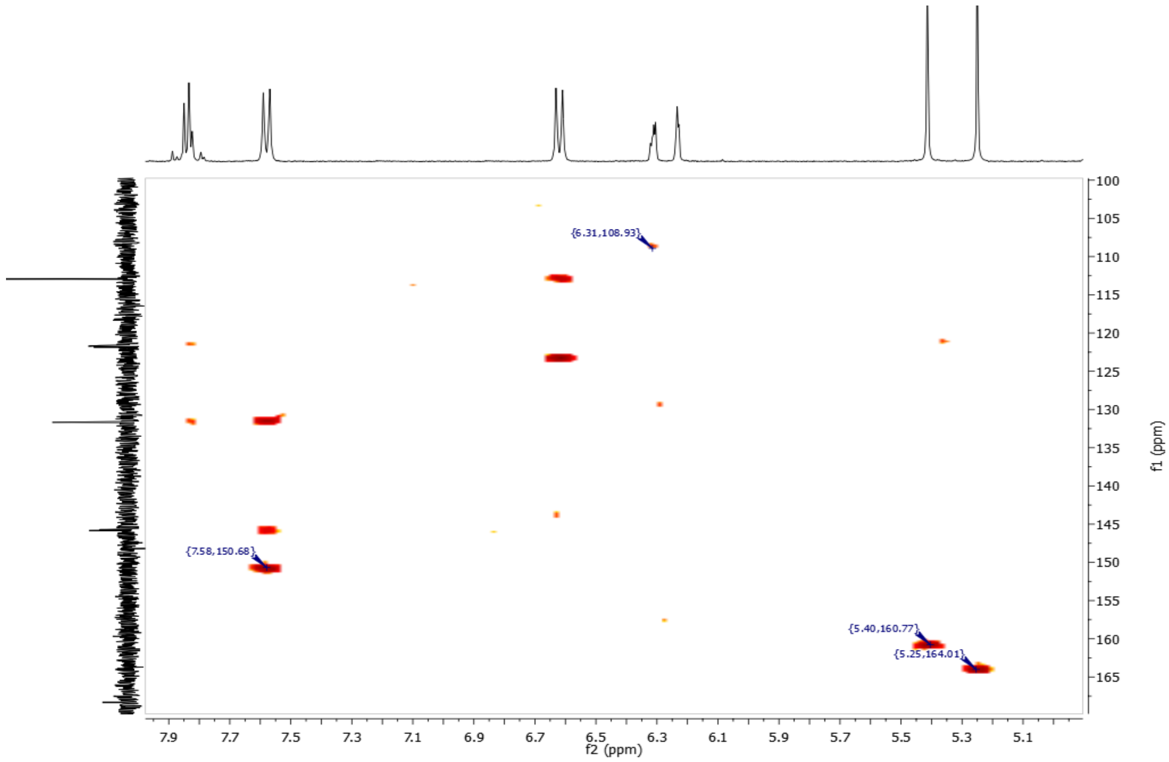

(2*E*)-1-[2,4-Bis(methoxymethoxy)-6-hydroxyphenyl]-3-[(4-dimethylamino)phenyl]prop-2-en-1-one (**28**). Purified by column chromatography (*iso*-hexane/THF 1:0 → 7:3). Reaction yield: 79%; LCMS: RT = 1.34 min, m/z = 388.0 $[M + H]^+$ (high pH method); red solid; m.p. = 114.3 – 115.4 °C. ^{1}H NMR $[(CD_3)_2CO]$ δ (ppm) 14.22 (s, 1H, OH-6′), 7.90, 7.86, 7.82, 7.78 (olefinic AB system, 2H, $J_{A\text{-}B}$ = 15.4 Hz, H-2, H-3), 7.59 (d, 2H, J_{ortho} = 8.9 Hz, H-2′′, H-6′′), 6.78 (d, 2H, H-3′′, H-5′′), 6.31 (d, 1H, J_{meta} = 2.3 Hz, H-3′), 6.23 (d, 1H, J_{meta} = 2.3 Hz, H-5′), 5.41 (s, 2H, OC*H*$_2$O), 5.25 (s, 2H, OC*H*$_2$O), 3.56 (s, 3H, OC*H*$_3$), 3.45 (s, 3H, OC*H*$_3$), 3.05 [s, 6H, N(C*H*$_3$)$_2$]. ^{13}C NMR $[(CD_3)_2CO]$ δ (ppm) 193.5 (C-1), 168.3 (C-6′), 164.2 (C-2′), 161.0 (C-4′), 153.4 (C-4′′), 145.4 (C-3), 131.5 (C-2′′, C-6′′), 123.9 (C-1′′), 122.5 (C-2), 113.0 (C-3′′, C-5′′), 108.3 (C-1′), 98.0 (C-5′), 96.3 (OCH_2O), 95.7 (C-3′), 95.0 (OCH_2O), 57.3 (OCH_3), 56.7 (OCH_3), 40.3 [$N(CH_3)_2$].

LCMS

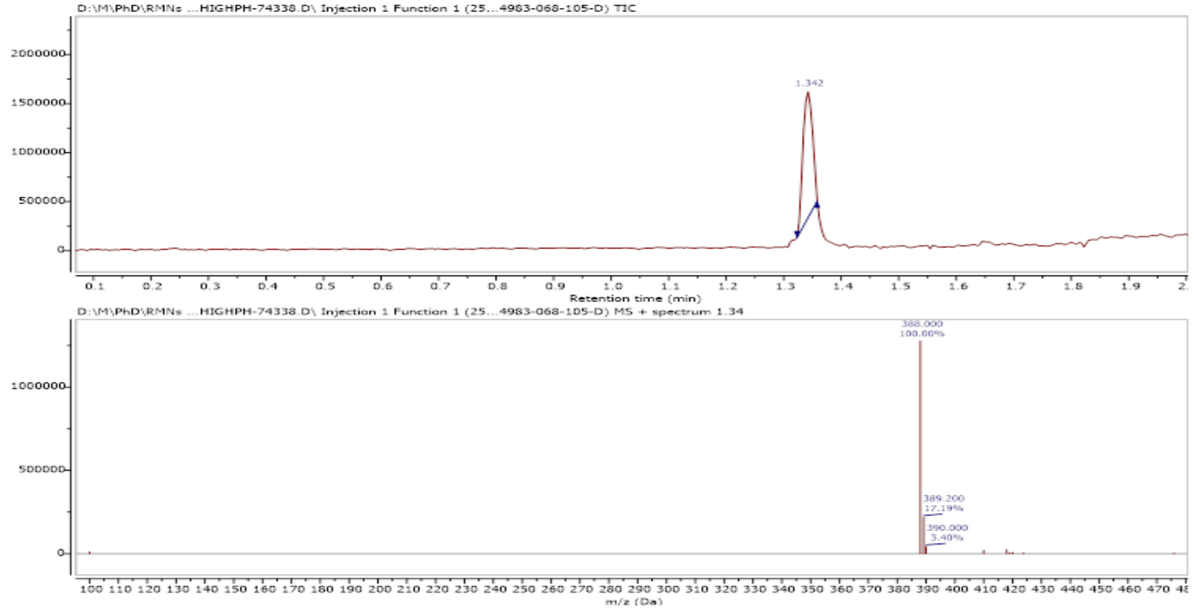

^{1}H NMR – Acetone-d_6

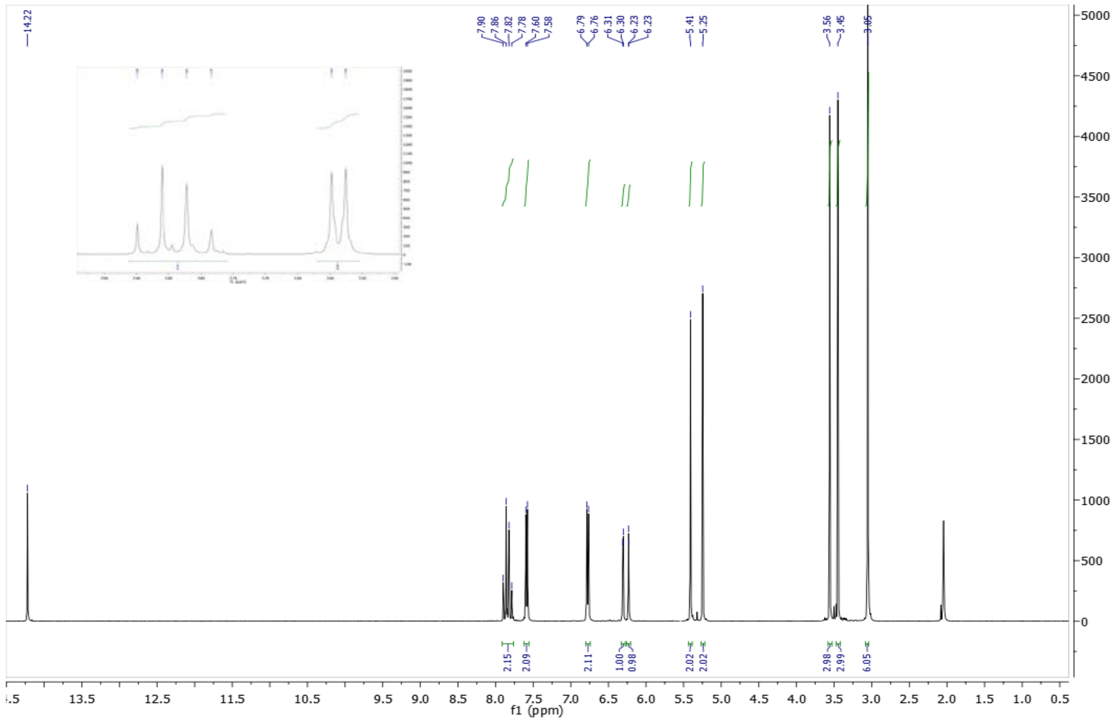

^{13}C NMR – Acetone-d_6

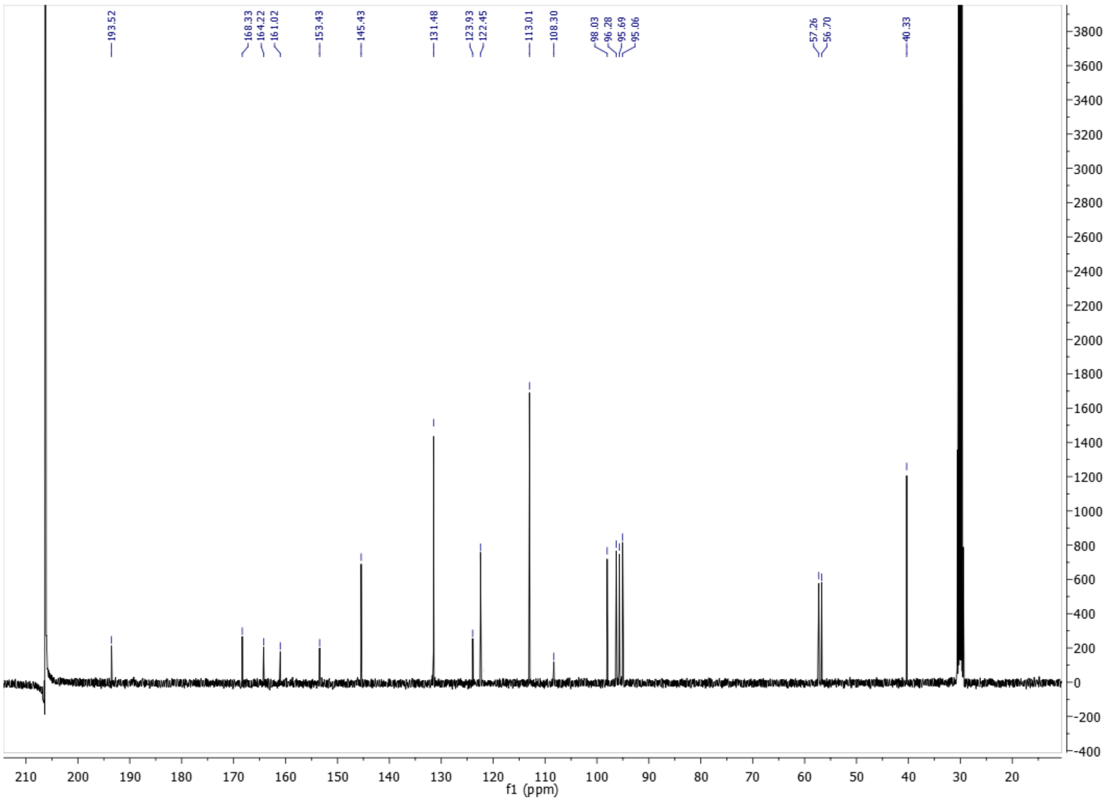

(2*E*)-1-[2,4-Bis(methoxymethoxy)-6-hydroxyphenyl]-3-(furan-2-yl)prop-2-en-1-one (**29**). Purified by preparative HPLC. Reaction yield: 93%; LCMS: RT = 1.21 min, *m*/*z* = 335.0 $[M + H]^+$ (high pH method); yellow solid; m.p. = 72.8 – 73.4 °C. ^{1}H NMR ($CDCl_3$) δ (ppm) 13.90 (s, 1H, OH-6′), 7.84, 7.80 (part A of olefinic AB system, 1H, $J_{A\text{-}B}$ = 15.5 Hz, H-3), 7.61, 7.57 (part B of olefinic AB system, 1H, $J_{B\text{-}A}$ = 15.5 Hz, H-2), 7.51 (d, 1H, $J_{4''\text{-}3''}$ = 1.4 Hz, H-4′′), 6.68 (d, 1H, $J_{2''\text{-}3''}$ = 3.4 Hz, H-2′′), 6.51 (dd, 1H, $J_{3''\text{-}2''}$ = 3.4 Hz, $J_{3''\text{-}15''}$ = 1.7 Hz, H-3′′), 6.31 (d, 1H, J_{meta} = 2.3 Hz, H-3′), 6.25 (d, 1H, J_{meta} = 2.4 Hz, H-5′), 5.29 (s, 2H, OC*H*$_2$O), 5.19 (s, 2H, OC*H*$_2$O), 3.57 (s, 3H, OC*H*$_3$), 3.48 (s, 3H, OC*H*$_3$). ^{13}C NMR ($CDCl_3$) δ (ppm) 192.5 (C-1), 167.5 (C-6′), 163.6 (C-2′), 160.0 (C-4′), 152.4 (C-1′′), 144.9 (C-4′′), 129.3 (C-2), 125.0 (C-3), 115.8 (C-2′′), 112.8 (C-3′′), 107.7 (C-1′), 97.6 (C-5′), 95.1 (OCH_2O), 94.8 (C-3′), 94.2 (OCH_2O), 57.0 (OCH_3), 56.6 (OCH_3).

LCMS

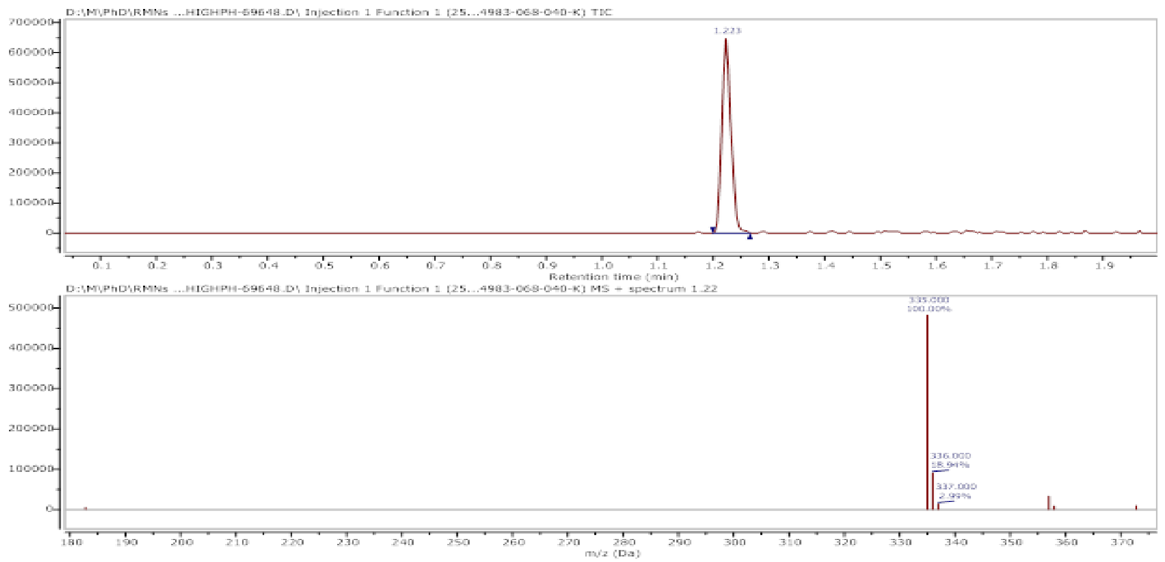

^{1}H NMR – Chloroform-*d*

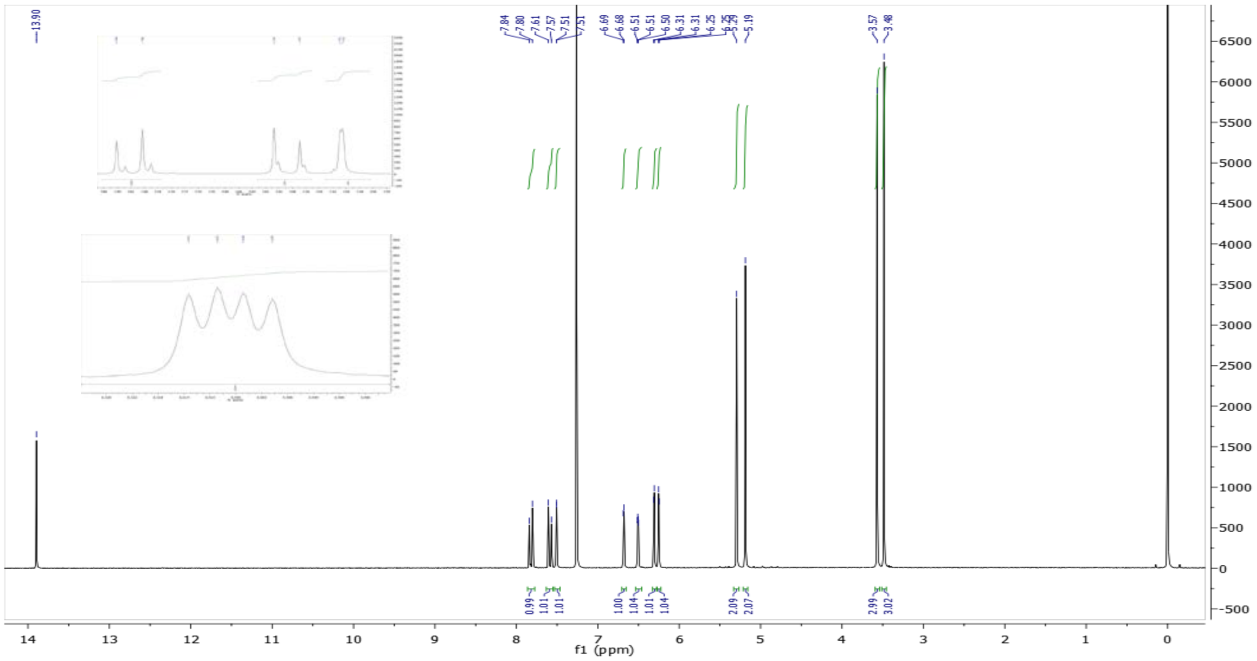

^{13}C NMR – Chloroform-*d*

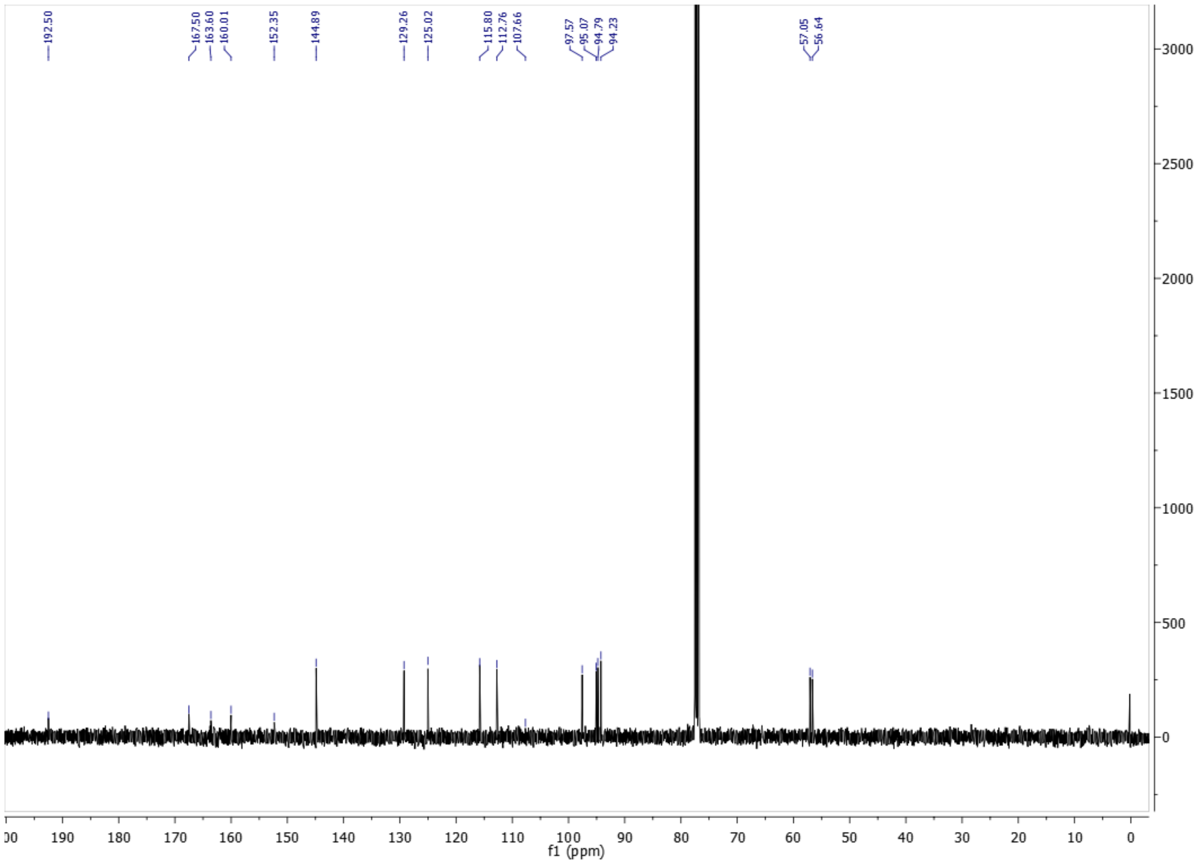

(2*E*)-1-[2,4-Bis(methoxymethoxy)-6-hydroxyphenyl]-3-(pyridin-4-yl)prop-2-en-1-one (**30**). This compound was the major product of the aldol condensation reaction that afforded it, as confirmed by LCMS. However, during/after purification by column chromatography (*iso*-hexane/THF), it was converted into an equilibrium between itself [LCMS: r.t. = 1.05 min, *m*/*z* = 346.0 $[M + H]^+$ (high pH method), 32%] and the corresponding flavanone [LCMS: r.t. = 0.82 min, *m*/*z* = 346.0 $[M + H]^+$ (high pH method), 59%]. The mixture was used in the subsequent reaction without further characterization or purification.

LCMS

Mixture used in the subsequent reaction without further purification.

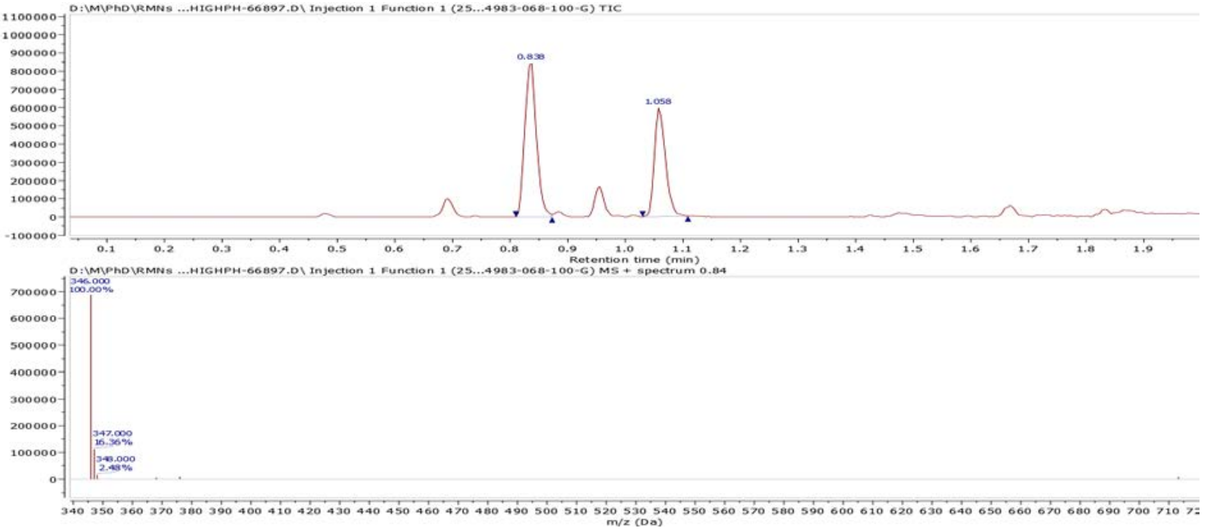

5,7-Bis(methoxymethoxy)-2-cyclobutylchroman-4-one (**31**). Purified by preparative HPLC. Reaction yield: 19%; LCMS: RT. = 1.13 min, *m*/*z* = 232.0 $[M + H]^+$ (high pH method); yellow oil. ^{1}H NMR ($CDCl_3$) δ (ppm) 6.38 (d, 1H, J_{meta} = 2.3 Hz, H-8), 6.33 (d, 1H, J_{meta} = 2.4 Hz, H-6), 5.25 – 5.17 (m, 4H, OC*H*2O), 4.34-4.23 (m, 1H, H-2), 3.52 (s, 3H, OC*H*3), 3.48 (s, 3H, OC*H*3), 2.62 (m, 1H, H-1), 2.51-2.48 (m, 2H, H-3a and H-3b), 2.13-1.85 (m, 6H, H-2′a and H-2′b, H-3′a and H-3′b, H-4′a and H-4′b). ^{13}C NMR ($CDCl_3$) δ (ppm) 190.1 (C-4), 164.9 (C-8a), 163.2 (C-7), 159.6 (C-5), 107.6 (C-4a), 97.9 (C-8), 97.3 (C-6), 95.2 (OCH_2O), 94.2 (OCH_2O), 80.5 (C-2), 56.6 (OCH_3), 41.7 (C-3), 39.0 (C-1′), 24.2, 23.4 (C-2′, C-4′), 18.3 (C-3′).

LCMS

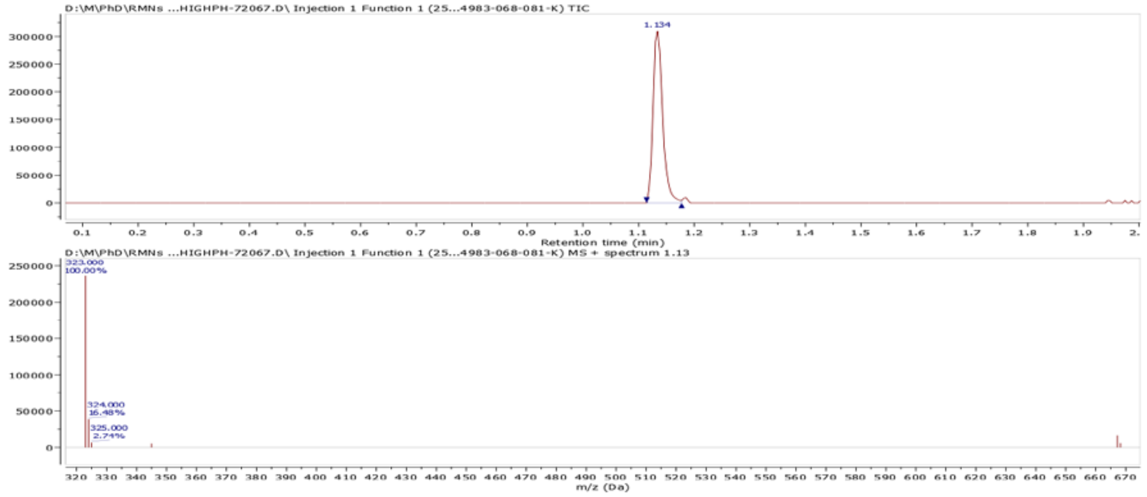

^{1}H NMR – Chloroform-*d*

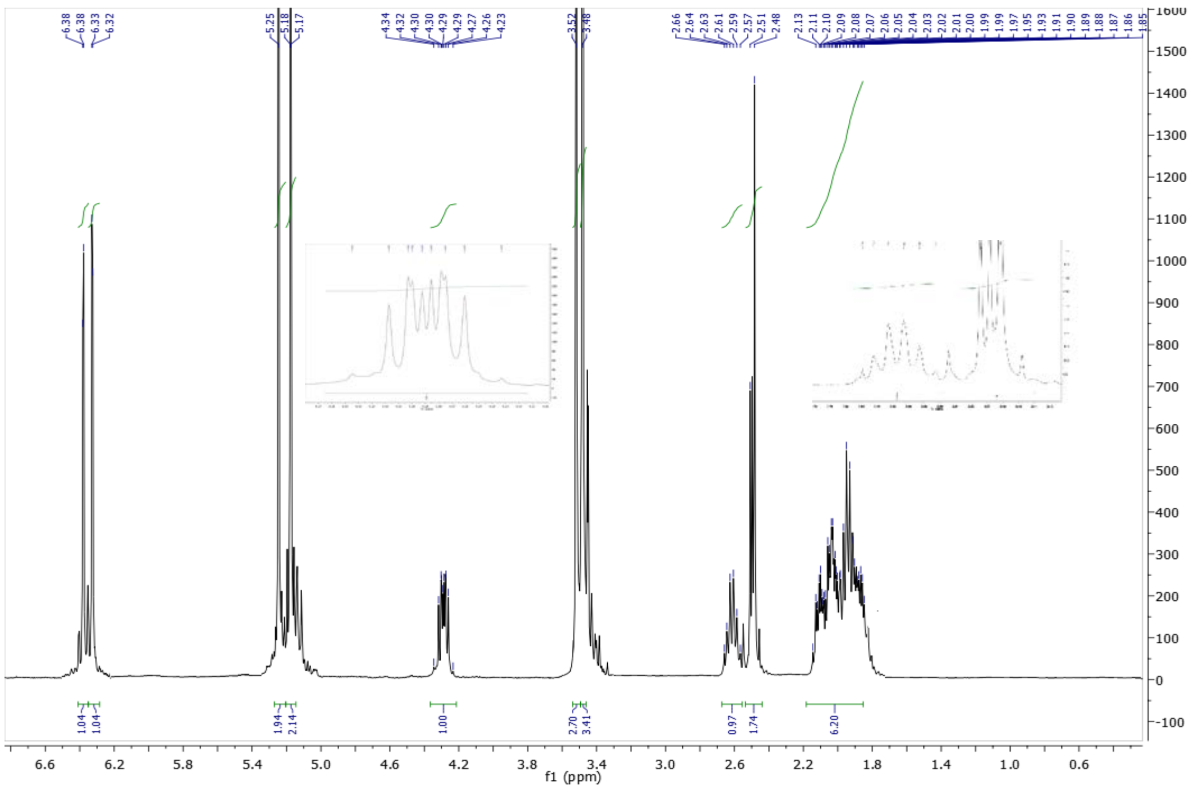

^{13}C NMR – Chloroform-*d*

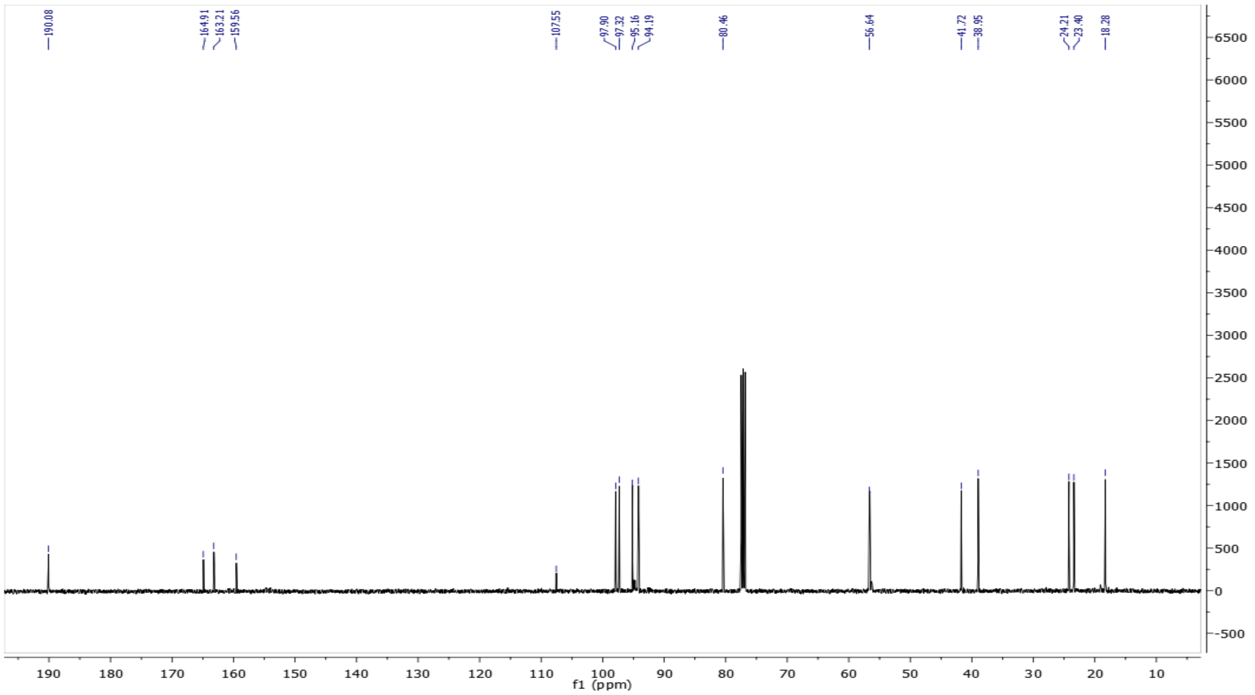

General procedure for the synthesis of benzyl-protected C-glucosyl chalcones. Compound **34** (synthesized according to a previously described methodology)[1] was dissolved in 1,4-dioxane (0.667 mmol in 8 mL) and the appropriate aldehyde (0.734 mmol, 1.1 eq.) was added. The mixture was stirred until fully homogenized. Then, an aqueous solution of NaOH 50% (w/v, 8 mL) was slowly added and the mixture was stirred under reflux for 18 h – 24 h. All reactions were followed by LCMS. Once the starting material was fully consumed, the mixture was allowed to reach room temperature. The reaction was quenched with HCl 2M, washed with brine and extracted with EtOAc (3 x 15 mL). The organic layers were combined, dried over $MgSO_4$, filtered and concentrated under vacuum. The residue was purified using the most adequate purification method(s) to afford compounds **35-41**.

(2*E*)-1-[4,6-dibenzyloxy-2-hydroxy-3-(2,3,4,6-tetra-O-benzyl-β-D-glucopyranosyl)phenyl]-3-cyclopropylprop-2-en-1-one (35). Purified by column chromatography (*iso*-Hexane/acetone 1:0 → 7:3). Isolated yield: 91%; LCMS: RT = 1.82 min (high pH method); yellow oil. ^{1}H NMR ($CDCl_3$) δ (ppm) 14.53, 14.15 (s, 1H, OH-2′)*, 7.46-7.10 (m, 29H, benzyl aromatics, part A of olefinic AB system, H-2), 6.99, 6.94 (d, 2H, J_{ortho} = 7.3 Hz, benzyl aromatics)*, 6.56, 6.49 (1H, part B of olefinic AB system, J_{trans} = 14.9 Hz, $J_{3\text{-}1''}$ = 10.2 Hz, H-3)*, 6.01, 5.95 (s, 1H, H-5′)*, 5.11 – 4.81 (m, 8H, Ph-C*H*$_2$, H-1′′′), 4.72 – 4.47 (m, 5H, Ph-C*H*$_2$; part A of AB system, H-4′′′), 4.31 – 4.21 (m, 2H, Ph-C*H*$_2$; part B of AB system, H-2′′′), 3.81 – 3.57 (m, 4H, H-3′′′, H-5′′′, H-6′′′a, H-6′′′b), 1.42-1.39 (m, 1H, H-1′′), 0.92-0.88 (m, 2H, H-2′′a and H-3′′a), 0.63-0.60 (m, 2H, H-2′′b and H-3′′b). ^{13}C NMR ($CDCl_3$) δ (ppm) 193.0, 192.8 (C-1)*, 165.7 (C-2′), 164.1 (C-4′), 162.0, 161.7 (C-6′)*, 153.0, 152.8 (C-3)*, 139.1, 138.7, 138.6, 136.5, 136.4, 135.8 (benzyl C_q-aromatics)*, 128.9 – 127.9 (benzyl *C*H-aromatics, C-2), 107.6, 107.3 (C-3′)*, 106.0 (C-1′), 89.8, 89.7 (C-5′)*, 88.0, 87.9 (C-5′′′)*, 79.9 (C-2′′′), 79.4, 79.3 (C-4′′′)*, 78.6, 78.5 (C-3′′′)*, 75.7, 75.6, 75.2, 75.1, 74.4 (CH_2-Ph)*, 73.6, 73.4 (C-1′′′)*, 73.0, 72.6, 71.3, 70.9, 70.3(CH_2-Ph)*, 69.5, 69.4 (C-6′′′)*, 15.3, 15.2 (C-1′′)*, 9.3 (C-2′′, C-3′′). *Two peaks were observed due to the presence of rotamers.

LCMS

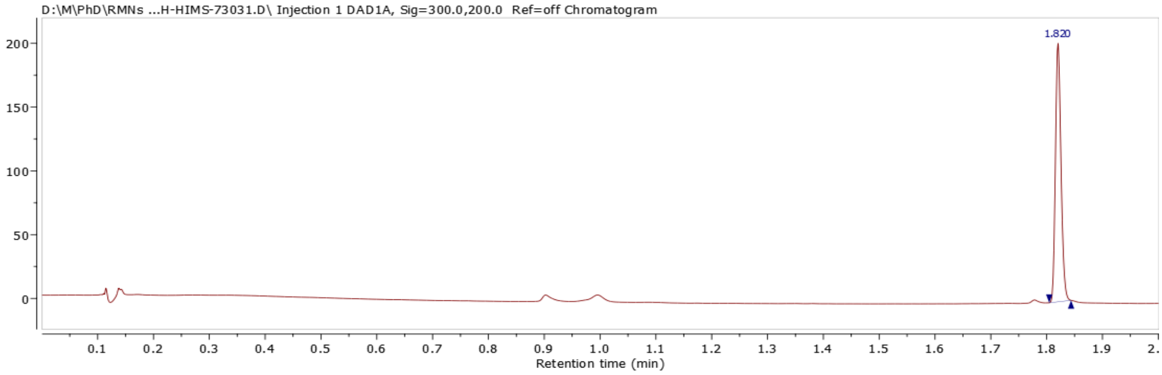

MS Detection range: 200-800 Da; MW of compound 26 = 923.10 Da

^{1}H NMR - Chloroform-*d*

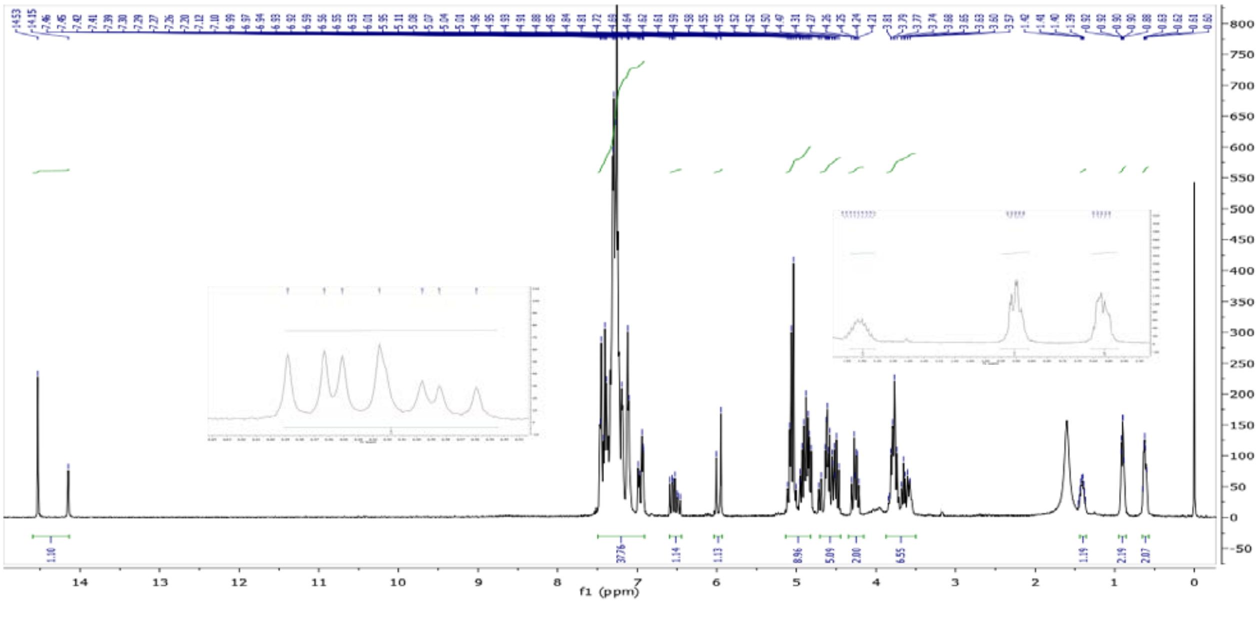

^{13}C NMR - Chloroform-*d*

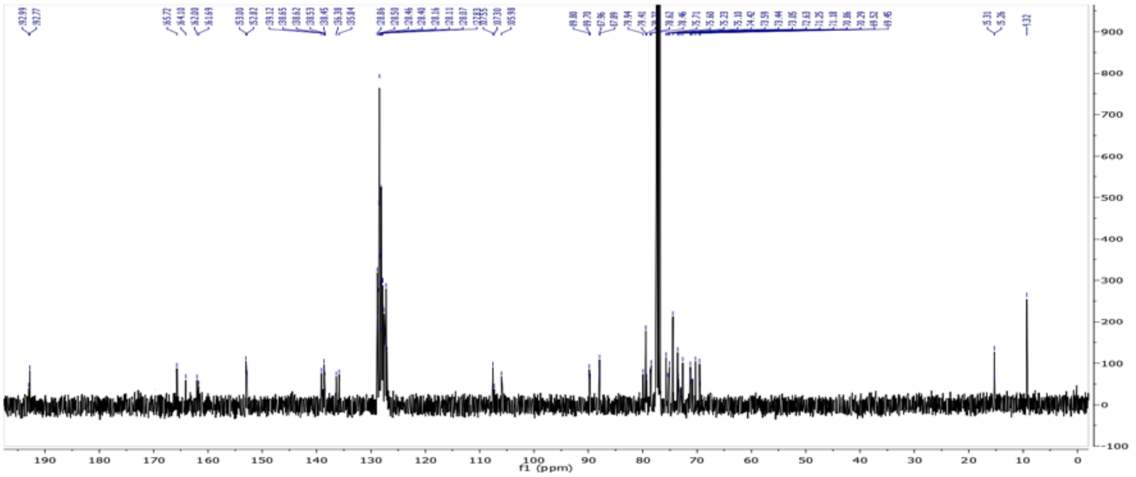

(2E)-1-[4,6-Dibenzyloxy-2-hydroxy-3-(2,3,4,6-tetra-*O*-benzyl-β-D-glucopyranosyl)phenyl]-3-phenylprop-2-en-1-one (36). Purified by column chromatography (cyclohexane/THF 1:0 → 17:3). Isolated yield: 72%; LCMS: RT = 1.95 min (high pH method); yellow oil. ^{1}H NMR ($CDCl_3$) δ (ppm) 14.82, 14.50 (s, 1H, OH-2′)*, 7.87 – 7.70 (olefinic AB system, 2H, J_{trans} = 15.4 Hz, H-2 and H-3)*, 7.50 – 6.95 (m, 35H, benzyl aromatics, H-2″, H-3″, H-4″, H-5″, H-6″), 6.07, 6.02 (s, 1H, H-5′)*, 5.15 – 4.83 (m, 8H, Ph-C*H*$_2$, H-1‴), 4.65 – 4.48 (m, 5H, Ph-C*H*$_2$; part A of AB system, H-4‴), 4.36 – 4.24 (m, 2H, Ph-C*H*$_2$; part B of AB system, H-2‴), 3.83 – 3.73 (m, 4H, H-3‴, H-5‴, H-6‴a and H-6‴b). ^{13}C NMR ($CDCl_3$) δ (ppm) 193.1, 192.9 (C-1)*, 166.9, 166.1 (C-2′)*, 164.4, 164.0 (C-4′)*, 162.3, 162.0 (C-6′)*, 143.0, 142.7 (C-3)*, 138.6(7), 138.6(6), 138.6, 138.5, 136.3, 135.4(4), 135.3(7) (benzyl C$_q$-aromatics)*, 128.8, 128.5, 128.4(8), 128.2, 128.1, 127.7 (benzyl *C*H-aromatics, C-1″, C-2″, C-3″, C-4″, C-5″, C-6″ and C-2), 107.7, 107.5 (C-3′)*, 106.6, 106.4 (C-1′)*, 89.4, 89.3 (C-5′)*, 88.0 (C-5‴), 79.9(9), 79.5 (C-2‴)*, 79.4(5), 79.2 (C-4‴)*, 78.7, 78.5 (C-3‴)*, 75.8, 75.6, 75.4, 75.1, 74.5, 74.4, 73.6, 73.5 (*C*H$_2$-Ph)*, 73.0, 72.6 (C-1‴)*, 71.5, 71.4(5), 70.9, 70.4 (*C*H$_2$-Ph)*, 69.6, 69.5 (C-6‴)*. *Two peaks were observed due to the presence of rotamers.

LCMS

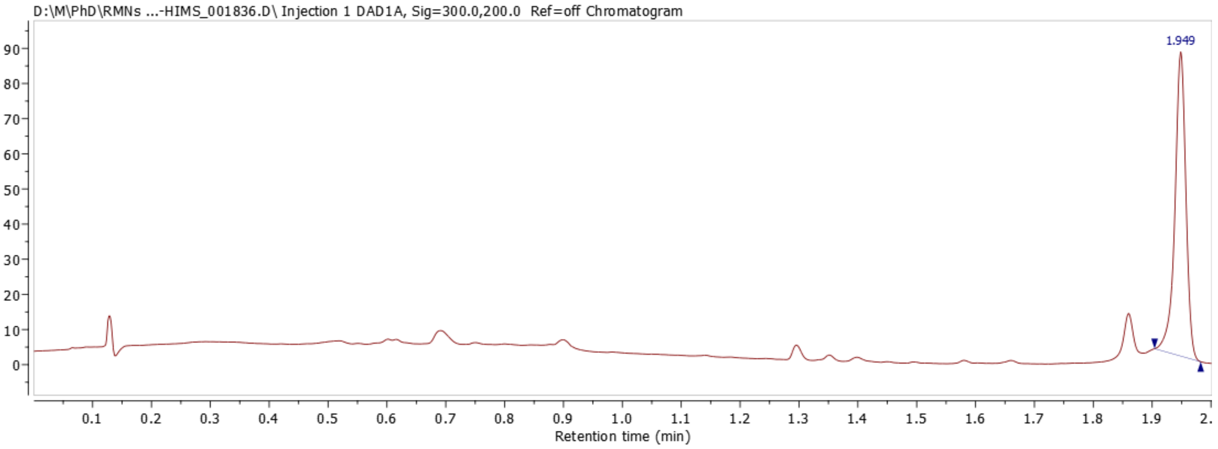

MS Detection range: 200-800 Da; MW of compound 27 = 959.13 Da

^{1}H NMR – Chloroform-*d*

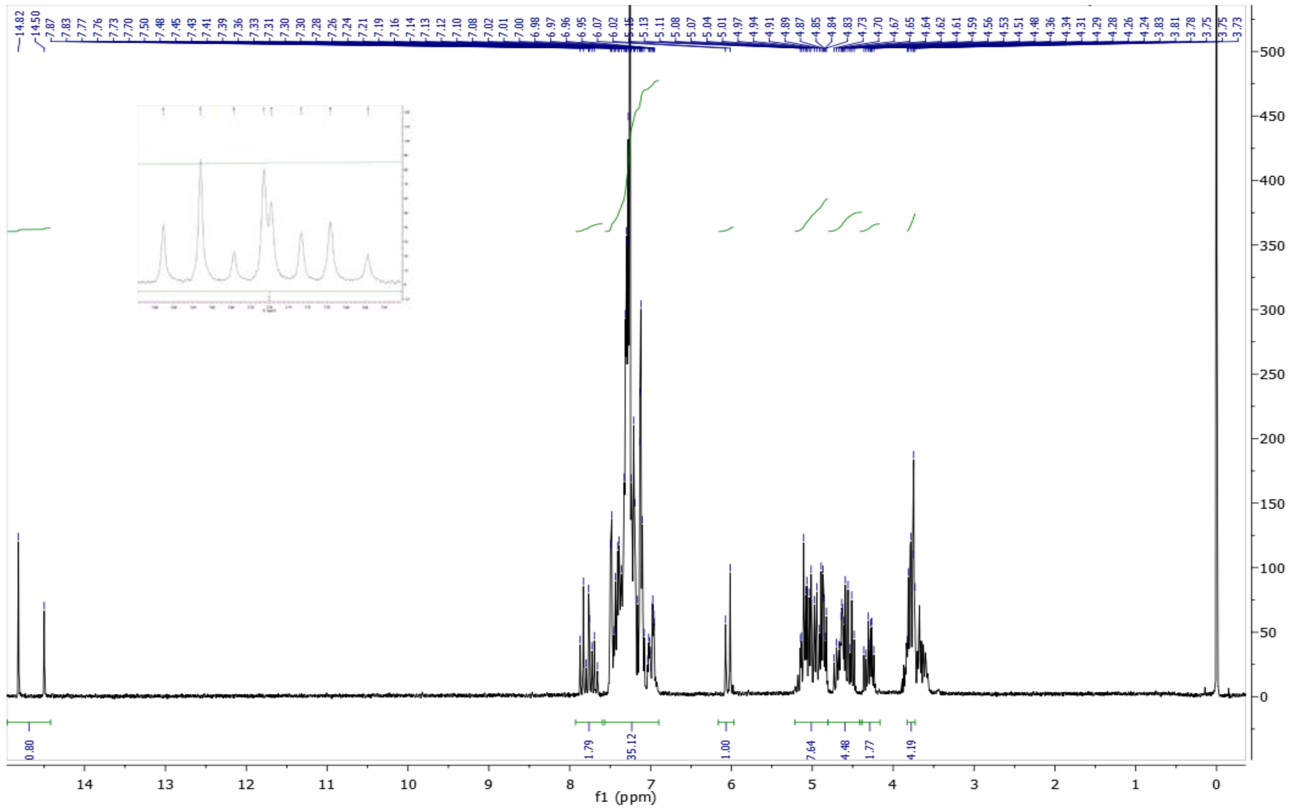

^{13}C NMR – Chloroform-*d*

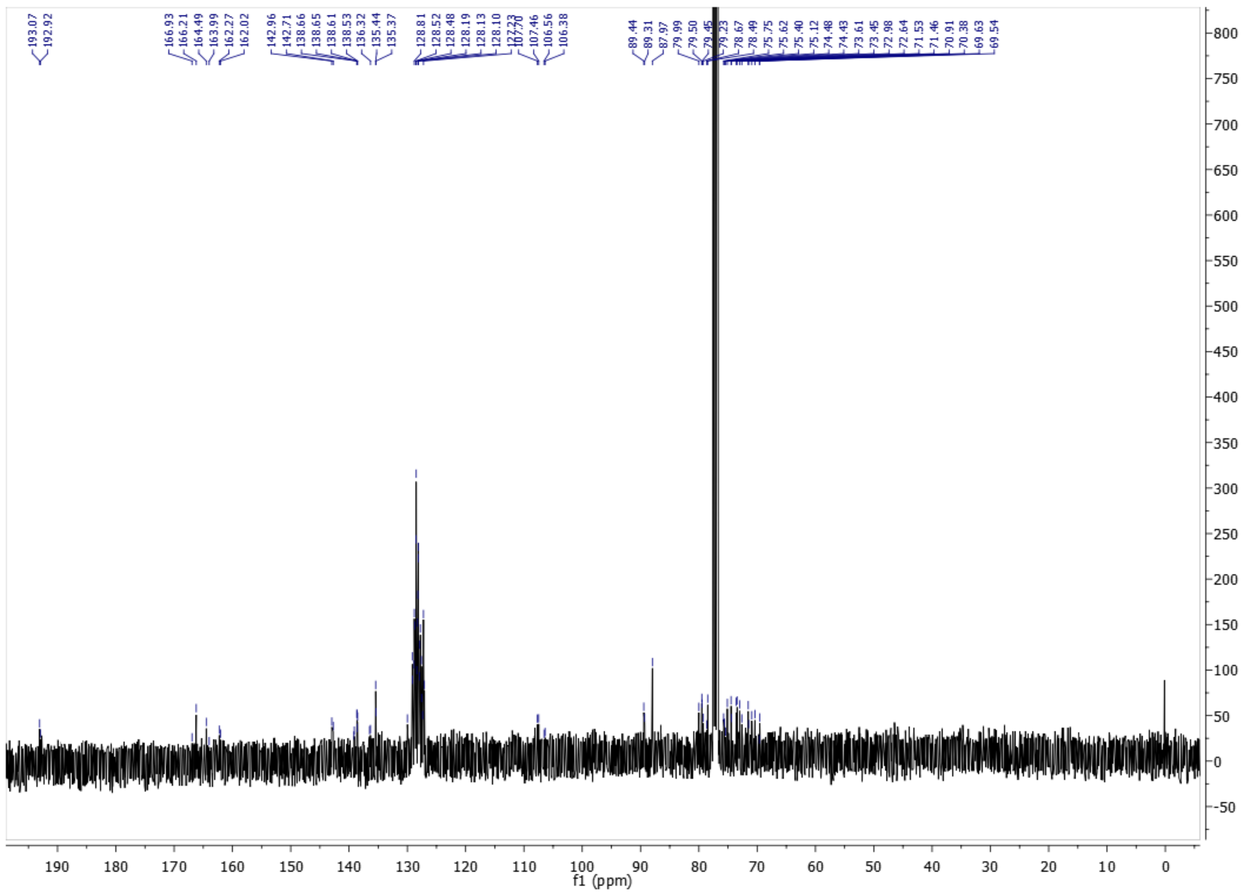

(2*E*)-1-[4,6-Dibenzyloxy-2-hydroxy-3-(2,3,4,6-tetra-*O*-benzyl-β-D-glucopyranosyl)phenyl]-3-(4-benzyloxyphenyl)prop-2-en-1-one (37). Purified by column chromatography (10:1 → 5:1 P.Ether-acetone). Isolated yield: 83%; R_f: 0.38 (3:1 Petroleum ether-acetone); %; LCMS: RT = 1.92 min (high pH method); yellow oil. ^{1}H NMR ($CDCl_3$) δ (ppm) 15.01, 14.72 (s, 1H, OH-2´)*, 7.79-7.71, 7.71-7.63 (olefinic AB-system, 2H, J_{AB} = 16.29 Hz, H-2 and H-3)*, 7.50-7.12 (m, 34H, benzyl aromatics), 7.04-6.96 (m, 3H, benzyl aromatics, H-4´, H-6´), 6.83-6.77 (m, 2H, H-3´, H-5´), 6.07, 6.01 (s, 1H, Ph-H5´)*, 5.17-4.48 (m, 14H, Ph-C*H*$_2$, H-1´´´), 4.36-4.24 (m, 2H, Ph-C*H*$_2$, H-2´´´), 3.85-3.58 (m, 5H, H-3´´´, H-4´´´, H-5´´´, H-6´´´a, H-6´´´b). ^{13}C NMR ($CDCl_3$) δ (ppm) 193.2, 192.9 (C-1)*, 167.0, 166.3 (C-2´)*, 164.3, 163.5 (C-4´)*, 162.2, 162.1 (C-6´)*, 160.4 (C-4´´), 143.0, 142.8 (C-3)*, 139.1 138.6, 138.5, 136.7, 136.5, 136.3, 135.4 (benzyl C_q-aromatics), 130.3-127.1 (benzyl CH-aromatics), 125.6, 125.5 (C-2)*, 115.1 (C-2´´, C-3´´, C-5´´, C-6´´), 107.6 (C-3´), 106.2 (C-1´), 89.3, 89.1 (C-5´)*, 88.0 (C-3´´´), 79.4, 79.2 (C-2´´´)*, 78.4, 78.3 (C-4´´´, C-5´´´), 77.4, 75.8, 75.6, 74.4, 73.6, (CH_2-Ph)*, 72.8, 72.6 (C-1´´´)*, 71.5, 70.9 (CH_2-Ph), 70.2 (C-6´´). *Two peaks were observed due to the presence of rotamers.

LCMS

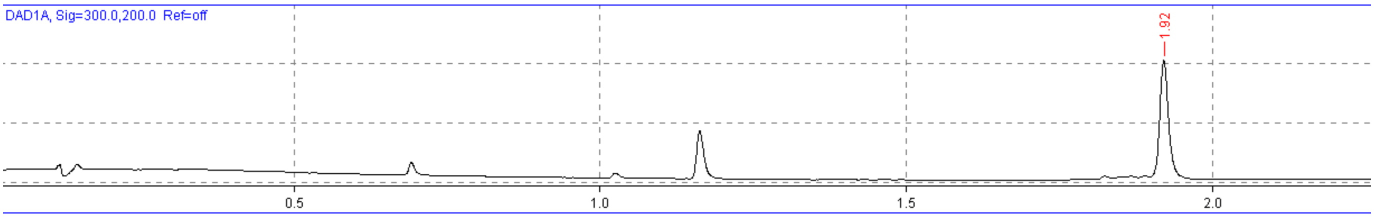

MS Detection range: 200-800 Da; MW of compound 28 = 1065.25 Da

^{1}H NMR – Chloroform-*d*

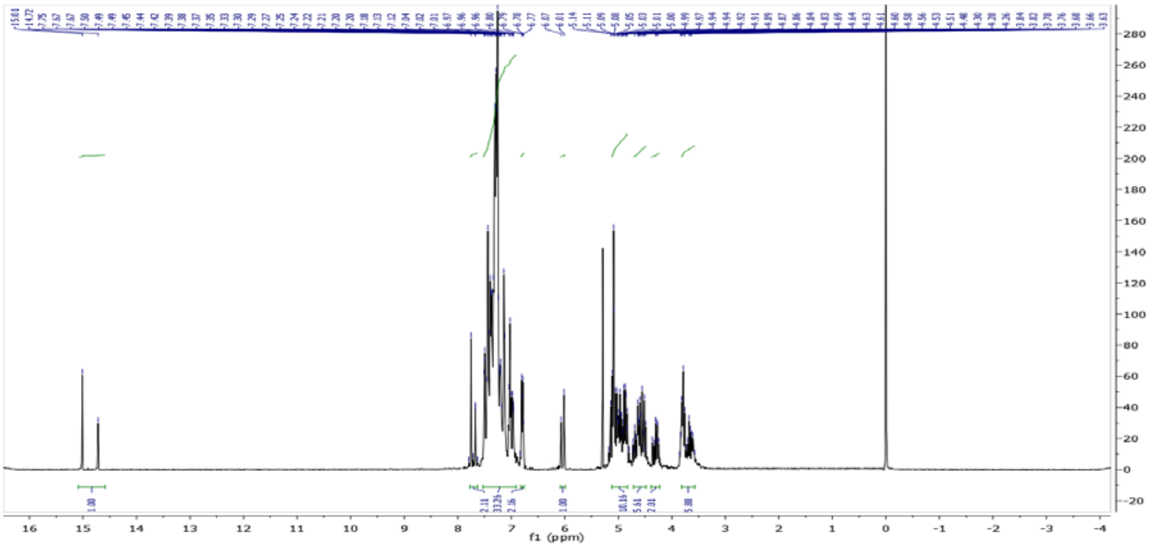

^{13}C NMR – Chloroform-*d*

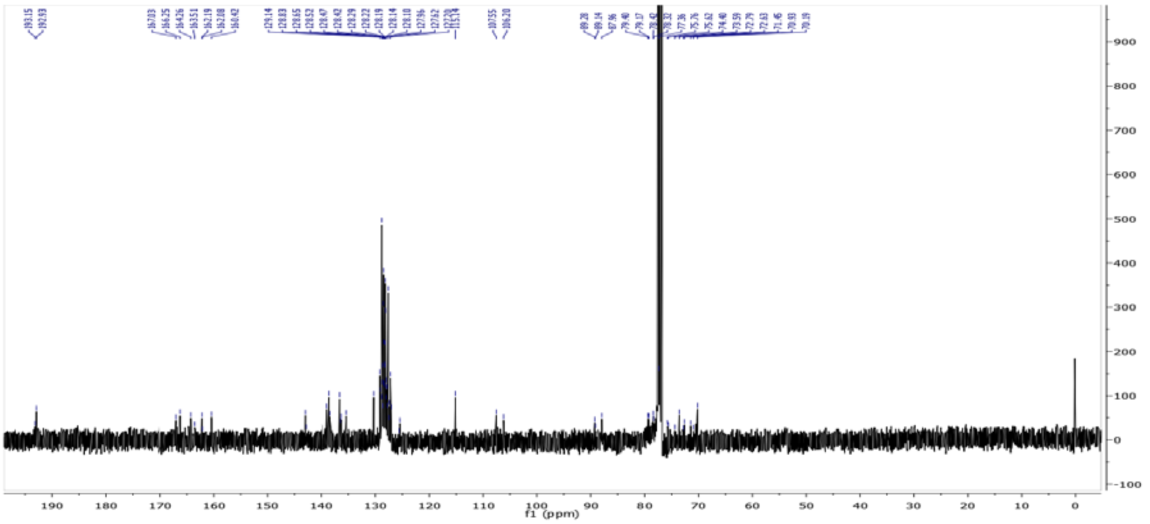

(2*E*)-1-[4,6-Dibenzyloxy-2-hydroxy-3-(2,3,4,6-tetra-*O*-benzyl-β-D-glucopyranosyl)phenyl]-3-(4-fluorophenyl)prop-2-en-1-one (38). Purified by column chromatography (*iso*-Hexane/THF 1:0 → 7:3). Isolated yield: 66%; LCMS: RT = 1.88 min (high pH method); yellow oil. ^{1}H NMR ($CDCl_3$) δ (ppm) 14.84, 14.60 (s, 1H, OH-2′)*, 7.71, 7.61 (olefinic AB system, 2H, J_{trans} = 15.5 Hz, H-2, H-3)*, 7.50-6.83 (m, 34H, benzyl aromatics, H-2′′, H-3′′, H-5′′, H-6′′), 6.07, 6.02 (s, 1H, H-5′)*, 5.16 – 4.83 (m, 8H, Ph-C*H*$_2$, H-1′′′), 4.71 – 4.46 (m, 5H, Ph-C*H*$_2$; part A of AB system, H-4′′′), 4.36 – 4.24 (m, 2H, Ph-C*H*$_2$; part B of AB system, H-2′′′), 3.84 – 3.57 (m, 4H, H-3′′′, H-5′′′, H-6′′′a, H-6′′′b). ^{13}C NMR ($CDCl_3$) δ (ppm) 193.0, 192.8 (C-1)*, 167.0, 166.2 (C-2′)*, 164.5, 163.8 (C-4′)*, 163.4, 163.3 (d, $J_{C\text{-}F}$ = 252.5 Hz, C-4′′)*, 162.3, 162.1 (C-6′)*, 141.5, 141.2 (C-3)*, 139.1(4), 139.0(7), 138.6(5), 138.6(0), 138.5(2), 138.4(7), 136.4, 136.3, 135.5, 135.3 (benzyl C_q-aromatics)*, 129.2 – 127.1 (benzyl *C*H-aromatics, C-1′′, C-2′′, C-6′′), 115.9, 115.8 (d, $J_{C\text{-}F}$ = 22.1 Hz, C-3′′, C-5′′)*, 107.7, 107.3 (C-3′)*, 106.7, 106.1 (C-1′)*, 89.3, 89.2 (C-5′)*, 87.9 (C-5′′′), 79.9, 79.5 (C-2′′′)*, 79.4, 79.2 (C-4′′′)*, 78.7, 78.5 (C-3′′′)*, 75.7, 75.6, 75.3, 75.1, 74.5, 74.4, 73.6, 73.4 (*C*H$_2$-Ph)*, 72.9, 72.6 (C-1′′′)*, 71.5, 71.4, 70.9, 70.4 (*C*H$_2$-Ph)*, 69.5(8), 69.5(5) (C-6′′′)*. *Two peaks were observed due to the presence of rotamers.

LCMS

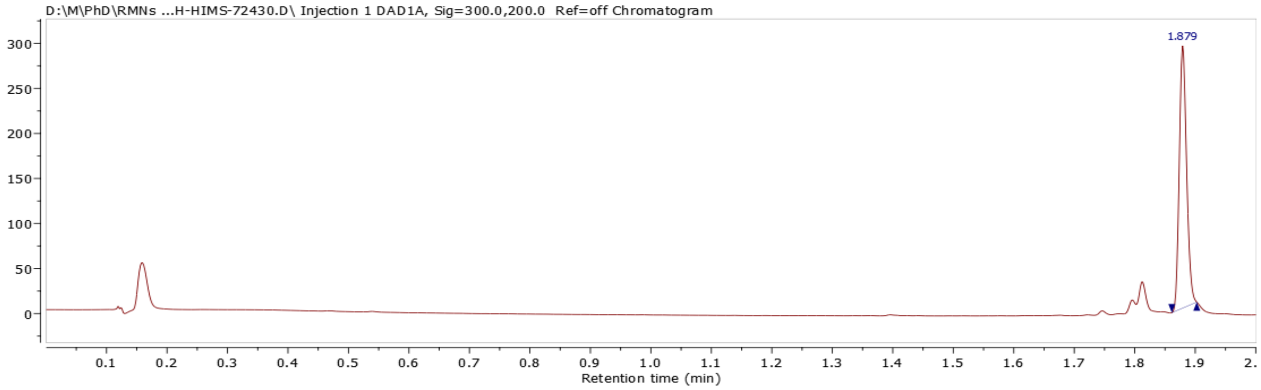

MS Detection range: 200-800 Da; MW of compound 29 = 977.12 Da

^{1}H NMR – Chloroform-*d*

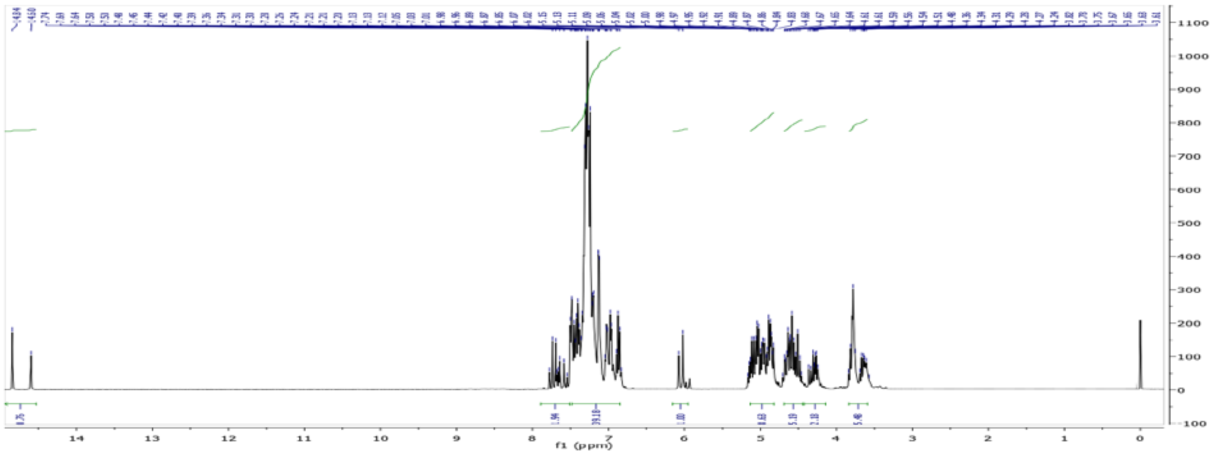

^{13}C NMR – Chloroform-*d*

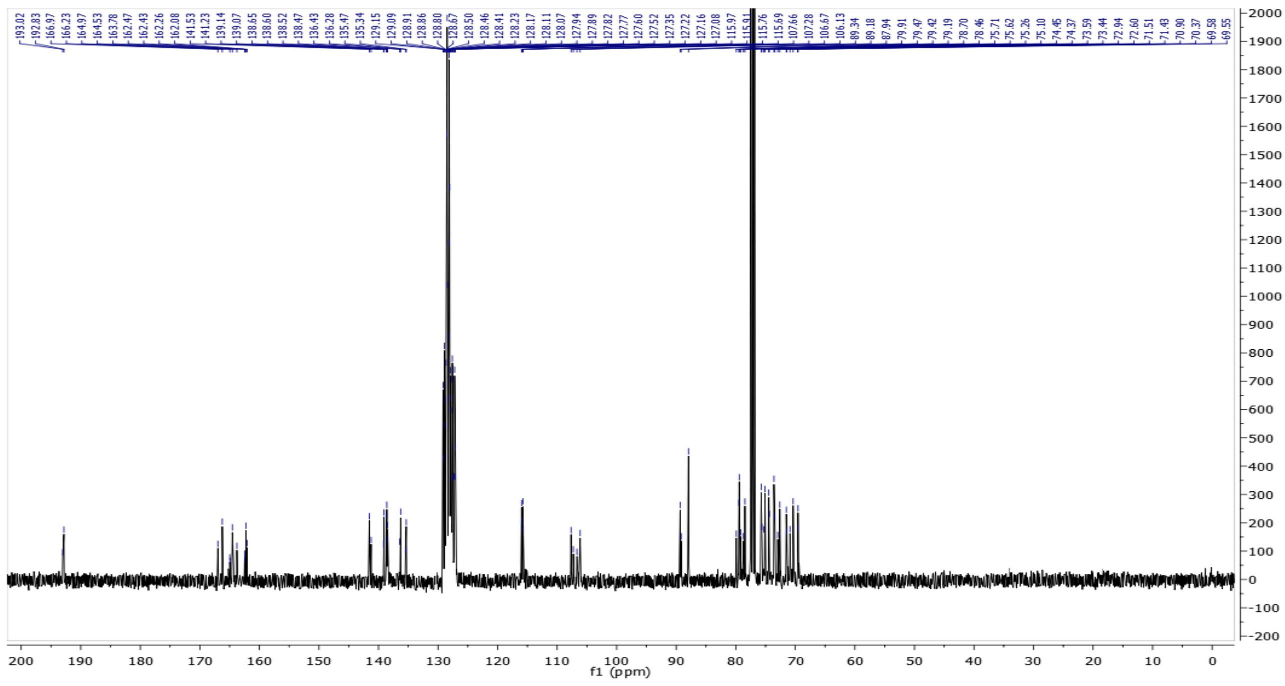

(2*E*)-1-[4,6-Dibenzyloxy-2-hydroxy-3-(2,3,4,6-tetra-*O*-benzyl-β-D-glucopyranosyl)phenyl]-3-[4-(morpholin-4-yl)phenyl]prop-2-en-1-one (39). Purified by column chromatography (cyclohexane/THF 1:0 → 3:1). Isolated yield: 67%; LCMS: RT = 1.83 min (high pH method); orange oil. ^{1}H NMR ($CDCl_3$) δ (ppm) 14.68, 14.67 (s, 1H, OH-2´)*, 7.76 – 7.69 (m, 2H, H-2 and H-3)*, 7.51 – 6.95 (m, 32H, benzyl aromatics, H-2´´ and H-6´´), 6.89 – 6.69 (m, 2H, H-3´´ and H-5´´)*, 6.06, 6.01 (s, 1H, H-5´)*, 5.16 – 4.83 (m, 8H, Ph-CH_2, H-1´´´), 4.73 – 4.45 (m, 5H, Ph-CH_2; part A of AB system, H-4´´´), 4.35 – 4.23 (m, 2H, Ph-CH_2; part B of AB system, H-2´´´), 3.87 – 3.60 (m, 8H, H-3´´´, H-5´´´, H-6´´´a, H-6´´´b, NCH_2CH_2O), 3.24 – 3.14 (m, 4H, NCH_2CH_2O)*. ^{13}C NMR ($CDCl_3$) δ (ppm) 192.9 (C-1), 166.2 (C-2´), 164.1 (C-4´), 162.1 (C-6´), 152.5, (C-4´´), 143.5 (C-3), 139.1, 138.7, 138.6, 136.4, 135.7 (benzyl C_q-aromatics)*, 130.3, 130.2 (C-1´´)*, 129.1, 128.8, 128.7, 128.6, 128.5, 128.4, 128.2, 128.1, 128.0, 127.9, 127.8, 127.6, 127..4, 127.2, 127.1, 126.6 (benzyl CH-aromatics, C-2´´ and C-6´´), 124.6, 124.3 (C-2)*, 114.7 (C-3´´ and C-5´´), 108.1, 107.7 (C-3´)*, 107.4, 107.1 (C-1´)*, 89.5 (C-5´), 88.0 (C-5´´´), 79.5 (C-2´´´), 79.5, 79.4 (C-4´´´)*, 78.5 (C-3´´´), 75.7, 75.2, 75.1, 74.5, 74.4, 73.6 (CH_2-Ph)*, 73.0, 72.7 (C-1´´´)*, 71.4(1), 71.3(6), 70.9, 70.3 (CH_2-Ph)*, 67.0, 66.8 (NCH_2CH_2O)*, 66.7 (C-6´´´), 49.5, 48.3 (NCH_2CH_2O).*Peaks due to the presence of rotamers were observed.

LCMS

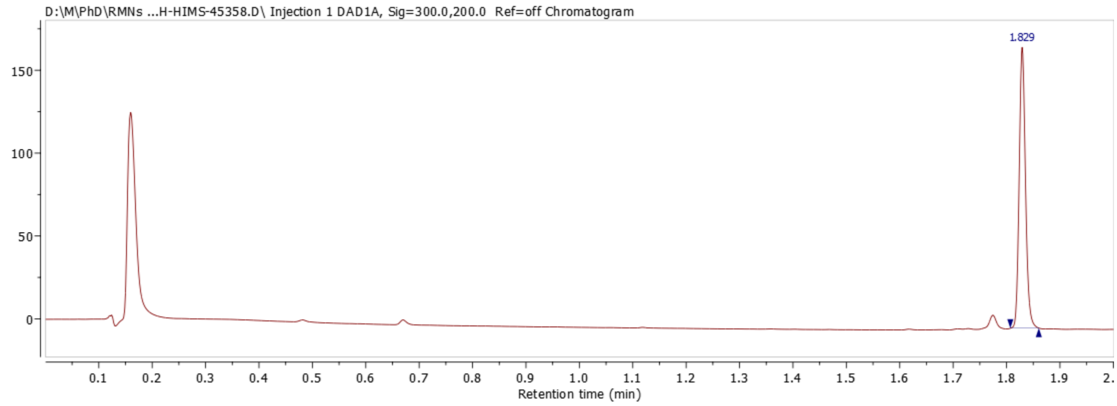

MS Detection range: 200-800 Da; MW of compound 30 = 1044.23 Da

^{1}H NMR – Chloroform-*d*

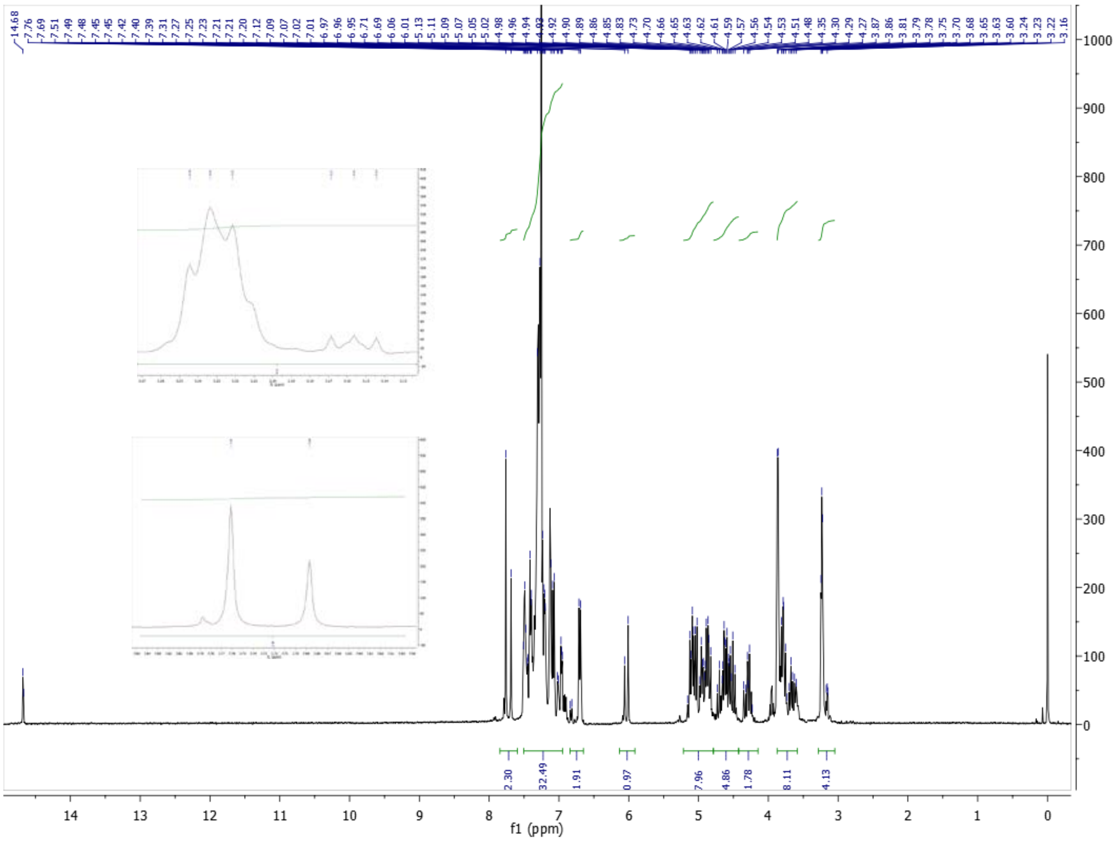

^{13}C NMR – Chloroform-*d*

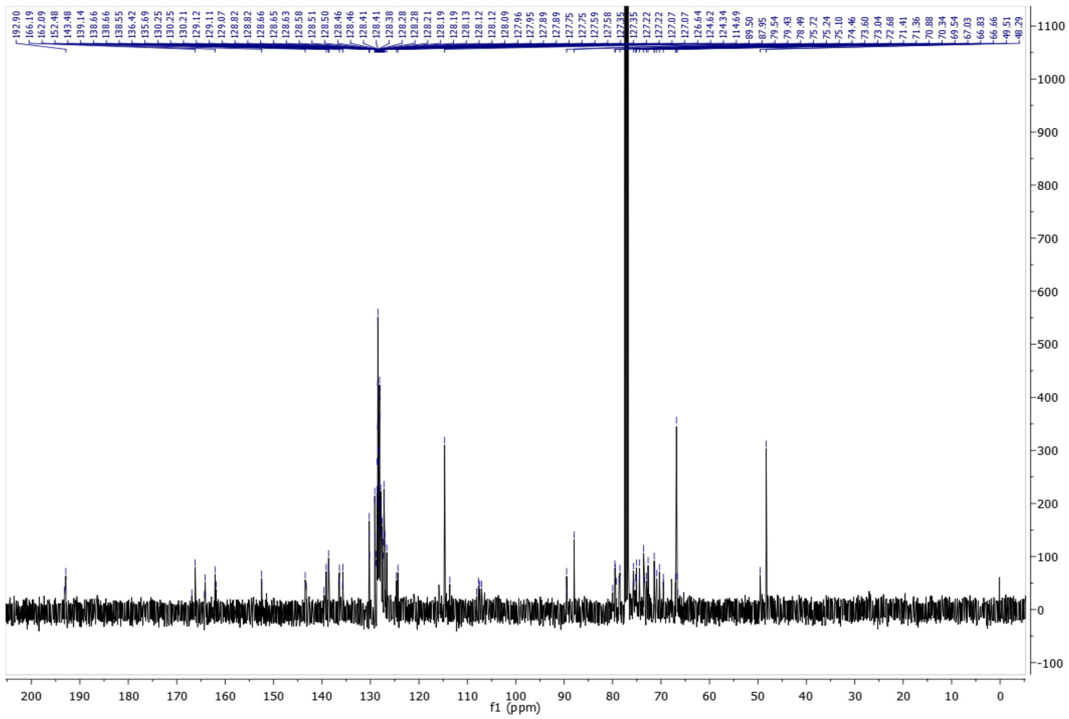

(2*E*)-1-[4,6-Dibenzyloxy-2-hydroxy-3-(2,3,4,6-tetra-*O*-benzyl-β-D-glucopyranosyl)phenyl]-3-[4-(pyrrolidin-1-yl)phenyl]prop-2-en-1-one (40). Purified by column chromatography (*iso*-Hexane/THF 1:0 → 3:2). Isolated yield: 75%; LCMS: RT = 1.93 min (high pH method); orange oil. ^{1}H NMR (CDCl$_3$) δ (ppm) 14.88 (s, 1H, OH-2′), 7.85-7.63 (m, olefinic AB system, 2H, J_{trans} = 15.5 Hz, H-2 and H-3)*, 7.51-6.95 (m, 32H, benzyl aromatics, C-2′′, C-6′′), 6.05, 6.01 (s, 1H, H-3′)*, 5.12 – 4.83 (m, 8H, Ph-C*H*$_2$, H-1′′′), 4.71 – 4.48 (m, 5H, Ph-C*H*$_2$; part A of AB system, H-4′′′), 4.35 – 4.25 (m, 2H, Ph-C*H*$_2$; part B of AB system, H-2′′′), 3.83 – 3.60 (m, 4H, H-3′′′, H-5′′′, H-6′′′a, H-6′′′b), 3.34-3.33 (m, 4H, NC*H*$_2$CH$_2$), 2.03-2.02 (m, 4H, NCH$_2$C*H*$_2$). ^{13}C NMR (CDCl$_3$) δ (ppm) 193.0, 192.7 (C-1)*, 166.9, 166.2 (C-2′)*, 163.8 (C-4′), 161.9(7) (C-6′), 149.4(4), 149.4(0) (C-4′′)*, 145.0, 144.8 (C-3)*, 139.2, 139.1, 138.7(1), 138.6(5), 136.5, 135.8(8), 135.8(3) (benzyl C$_q$-aromatics)*, 130.9, 130.8 (C-1′′)*, 129.1, 128.8, 128.6, 128.5, 128.4, 128.3, 128.2, 128.1, 128.0, 127.9, 127.8, 127.7, 127.6, 127.5, 127.4, 127.2, 127.1 (benzyl CH-aromatics, C-2′′ and C-6′′), 122.9, 122.8 (benzyl CH-aromatics)*, 121.9, 121.6 (C-2)*, 111.7 (C-3′′ and C-5′′), 108.1 (C-5′), 107.7 (C-1′), 89.5, 89.4 (C-3′)*, 87.9(5) (C-5′′′), 80.0 (C-2′′′), 79.4, 79.3 (C-4′′′)*, 78.5, 78.4 (C-3′′′)*, 75.7, 75.6, 75.2, 75.1, 74.5, 74.4, 73.6, 73.4 (CH$_2$-Ph)*, 73.1, 72.7 (C-1′′′)*, 71.4, 71.3, 70.3(0), 70.2(8) (CH$_2$-Ph)*, 69.5, 69.4 (C-6′′′)*, 48.1, 47.7 (NCH$_2$CH$_2$)*, 25.6 (NCH$_2$CH$_2$). *Peaks due to the presence of rotamers were observed.

LCMS

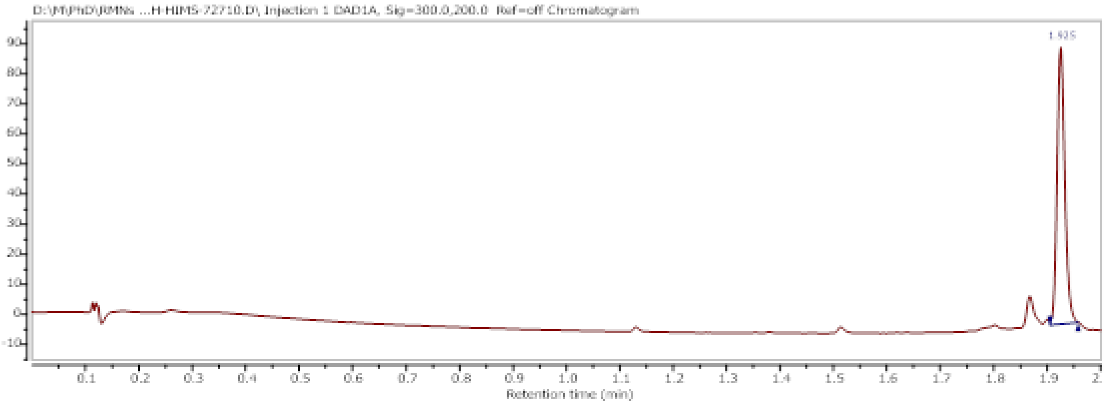

MS Detection range: 200-800 Da; MW of compound 31 = 1028.23 Da

^{1}H NMR – Chloroform-*d*

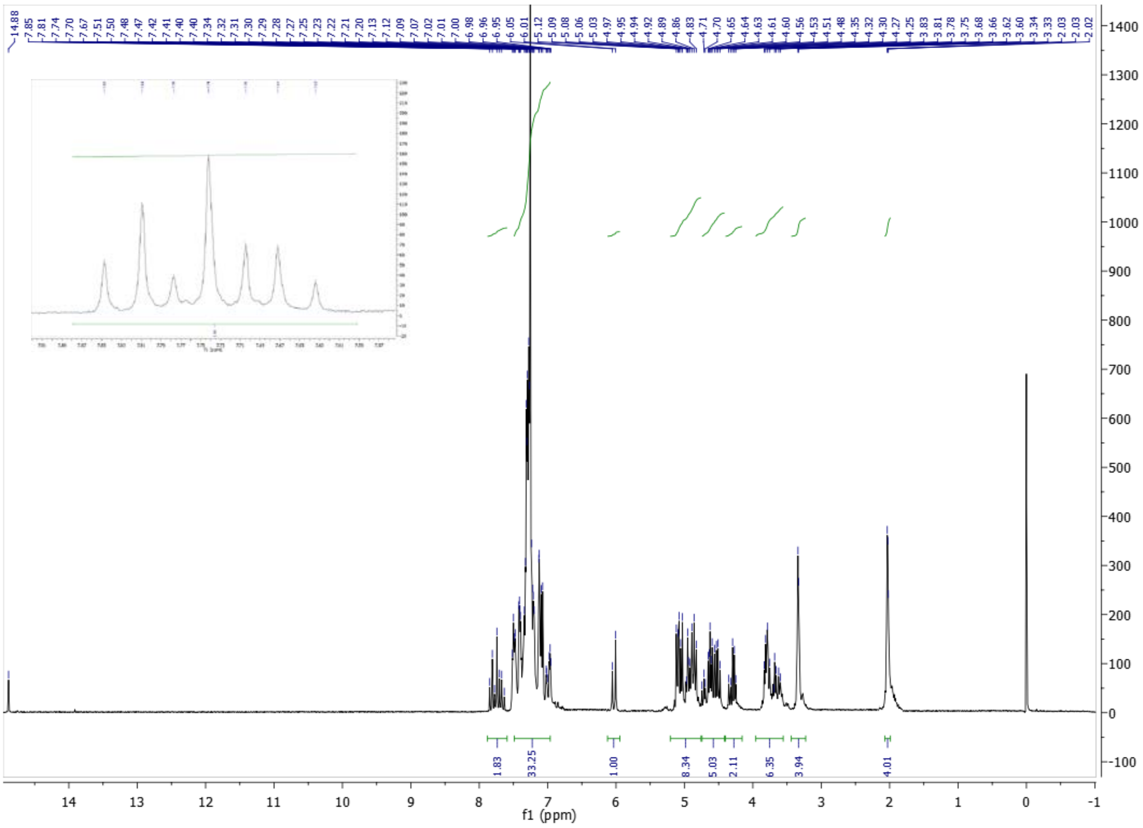

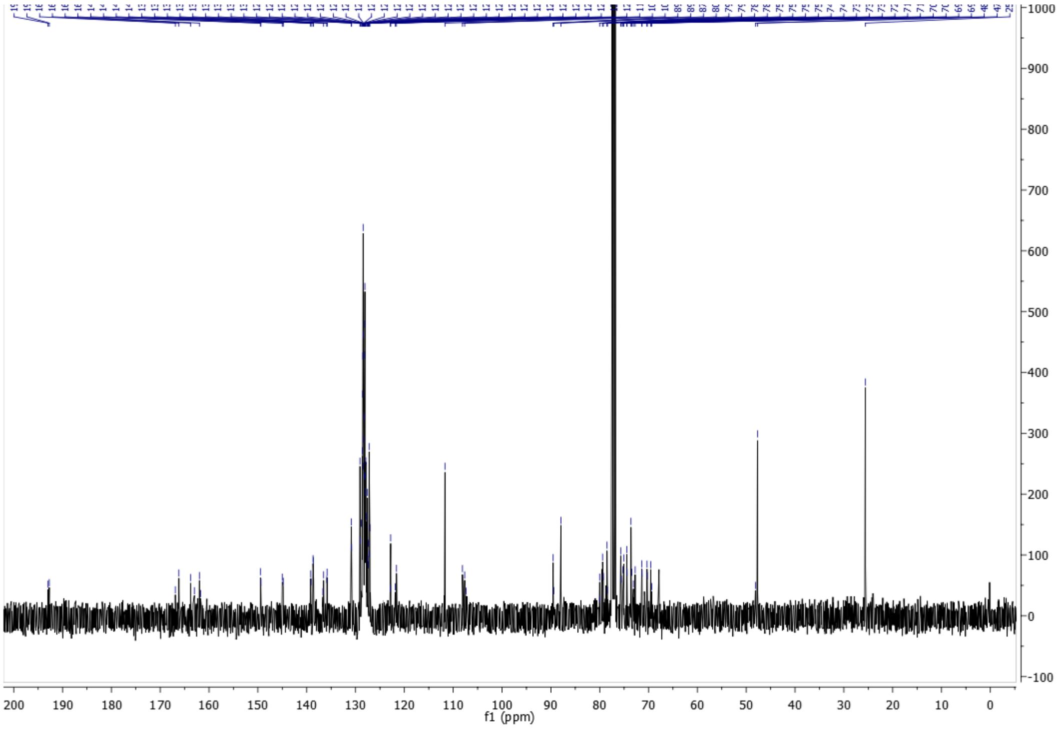

(2*E*)-1-[4,6-Dibenzyloxy-2-hydroxy-3-(2,3,4,6-tetra-*O*-benzyl-β-D-glucopyranosyl)phenyl]-3-(furan-2-yl)prop-2-en-1-one (41). Purified by column chromatography (*iso*-Hexane/EtOAc 1:0 → 7:3). Isolated yield: 82%; LCMS: RT = 1.88 min (high pH method); yellow oil. ^{1}H NMR ($CDCl_3$) δ (ppm) 14.59, 14.25 (s, 1H, OH-2´)*, 7.74, 7.67 (part A of olefinic AB system, 1H, J_{trans} = 15.3 Hz, H-3)*, 7.49, 7.43 (part B of olefinic AB system, 1H, J_{trans} = 15.4 Hz, H-2)*, 7.40-6.86 (m, 31H, benzyl aromatics, H-5´´), 6.45-6.43 (m, 1H, H-3´´)*, 6.36 (br s, 1H, H-4´´), 5.96, 5.91 (s, 1H, H-5´)*, 5.06 – 4.75 (m, 8H, Ph-C*H*$_2$, H-1´´´), 4.66 – 4.40 (m, 5H, Ph-C*H*$_2$; part A of AB system, H-4´´´), 4.27 – 4.15 (m, 2H, Ph-C*H*$_2$; part B of AB system, H-2´´´), 3.75 – 3.52 (m, 4H, H-3´´´, H-5´´´, H-6´´´a, H-6´´´b). ^{13}C NMR ($CDCl_3$) δ (ppm) 192.7, 192.5 (C-1)*, 166.5, 165.8 (C-2´)*, 164.3, 163.5 (C-4´)*, 162.1, 161.8 (C-6´)*, 152.3 (C-2´´), 144.7 (C-5´´), 139.1, 138.6, 136.3, 135.7 (benzyl C_q-aromatics), 128.5, 128.4, 128.1 (benzyl CH-aromatics, C-2), 125.9, 125.6 (C-3)*, 115.3, 115.2 (C-3´´)*, 112.5 (C-4´´), 107.6, 107.3 (C-3´)*, 106.8, 106.5 (C-1´)*, 89.8, 89.7 (C-5´)*, 87.9(9), 87.9(5) (C-5´´´)*, 80.0, 79.4 (C-2´´´)*, 79.2 (C-4´´´), 78.7, 78.5 (C-3´´´)*, 75.7, 75.6, 75.2, 75.1, 74.5, 73.6, 73.4 (CH_2-Ph)*, 73.0, 72.6 (C-1´´´)*, 71.4, 71.3, 70.9, 70.3 (CH_2-Ph)*, 67.2 (C-6´´´).
* Peaks were observed due to the presence of rotamers.

LCMS

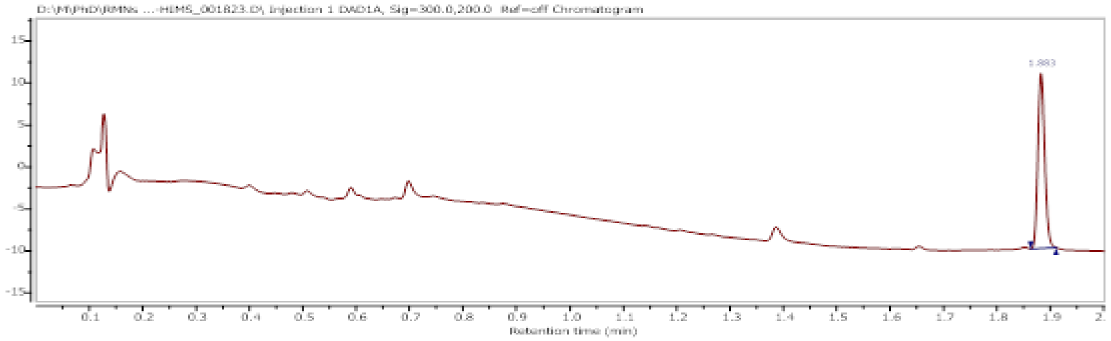

MS Detection range: 200-800 Da; MW of compound 32 = 949.09 Da

^{1}H NMR Chloroform-*d*

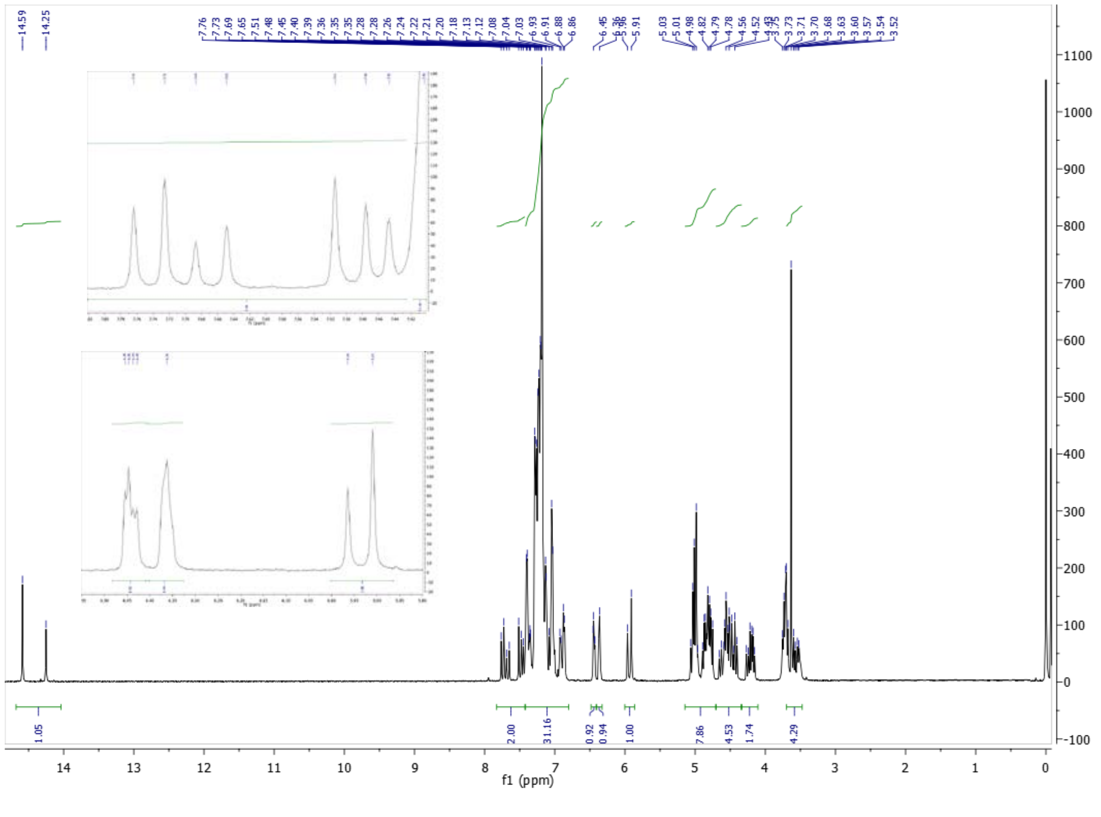

^{13}C NMR - Chloroform-*d*

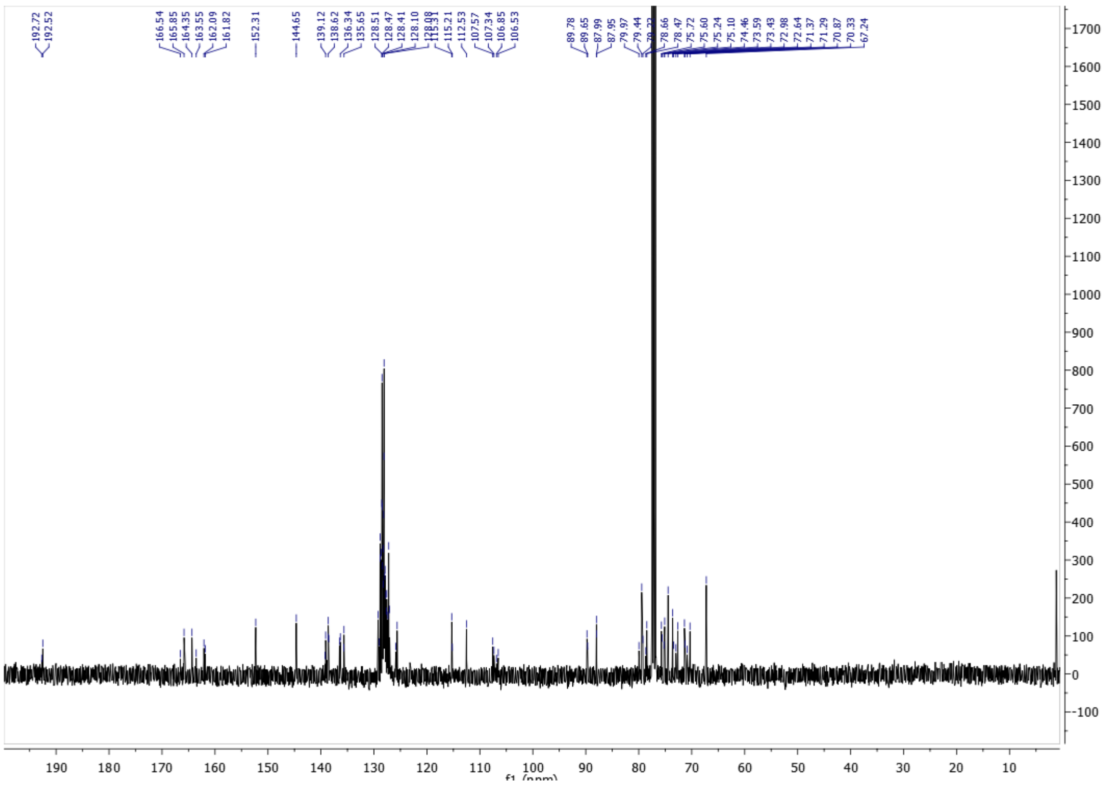

5,7-Dibenzyloxy-8-(2,3,4,6-tetra-*O*-benzyl-β-D-glucopyranosyl)-4*H*-chromen-4-one (42). Compound **34**[2] (0.230 g, 0.26 mmol, 1 eq.) was dissolved in ethyl formate (2.20 mL) at 0 °C under N_2 atmosphere. Then, NaH (60% dispersion in mineral oil, 0.063 g, 1.59 mmol, 6 eq.) was washed with cyclohexane three times and poured into the mixture. The reaction was left stirring vigorously at 0 °C for 15 minutes, after which the temperature was allowed to reach raised to room temperature. After 1 hour, the reaction was quenched with methanol (3 mLs) and, then, concentrated HCl (0.5 mL) was added to the mixture, which stirred under reflux for 18 hours until completion checked by LCMS. The reaction was quenched with a saturated solution of sodium hydrogenocarbonate (5 mL), washed with water (3 x 5 mL), extracted with DCM (3 x 10 mL), dried in a phase separator and concentrated under vacuum. The residue was purified by column chromatography (*iso*-hexane-diethyl ether 1:0 ® 1:4) and compound **40** was obtained as a colourless oil. Isolated yield: 84%; LCMS: RT = 1.75 min, *m*/*z* = 881.20 $[M + H]^+$ (high pH method). ^{1}H NMR ($CDCl_3$) δ (ppm) 7.66 (d, 0.5H, J_{cis} = 5.9 Hz, rotamer A, H-2)*, 7.58, 7.53 (d, 2H, J_{ortho} = 7.5 Hz, benzyl aromatics)*, 7.43-7.01 (m, 26.5 H, benzyl aromatics, rotamer B, H-2)*, 6.83-6.72 (d, 2H, J_{ortho} = 7.4 Hz, benzyl aromatics)*, 6.38 (s, 1H, H-6), 6.19, 6.09 (d, 1H, J_{cis} = 5.9 Hz, H-3)*, 5.25-4.80 (m, 8H, H-1′′′, Ph-C*H*2), 4.72-4.48 (m, 4H, Ph-C*H*2, part A_1 of A_1B_1 system), 4.29, 4.19 (t, 1H, $J_{2'''-1''',2'''-3'''}$ = 9.1 Hz, H-2′′′)*, 4.15, 4.08 (part B_1 of A_1B_1 system, 1H, J_{A-B} = 11.4 Hz, Ph-C*H*2)*, 3.90-3.53 (m, 5H, H-3′′′, H-4′′′, H-5′′′, H-6′′′a, H-6′′′b). ^{13}C NMR ($CDCl_3$) δ 177.0, 176.7 (C-4)*, 162.0, 161.1 (C-7)*, 160.1, 159.9 (C-5)*, 158.3, 157.6 (C-8a)*, 152.8, 152.5 (C-2)*, 138.9, 138.4, 137.9, 136.4, 136.0 (benzyl C_q-aromatics)*, 128.5, 128.1, 127.9, 127.4 (benzyl CH-aromatics), 114.3, 114.0 (C-3)*, 111.4, 110.5 (C-4a)*, 107.9, 107.7 (C-8)*, 96.2, 96.0 (C-6)*, 87.9, 87.8 (C-5′′′)*, 79.8, 79.5 (C-2′′′)*, 79.4, 79.2 (C-4′′′)*, 78.5, 78.4 (C-3′′′)*, 76.0, 75.7, 75.3, 75.2, 74.7, 74.4, 73.6, 73.3 (CH_2-Ph)*, 72.9, 72.6 (C-1′′′)*, 71.3, 71.1(3), 71.0(7), 70.9 (CH_2-Ph)*, 69.4, 69.1 (C-6′′′)*. *Peaks were observed due to the presence of rotamers.

LCMS

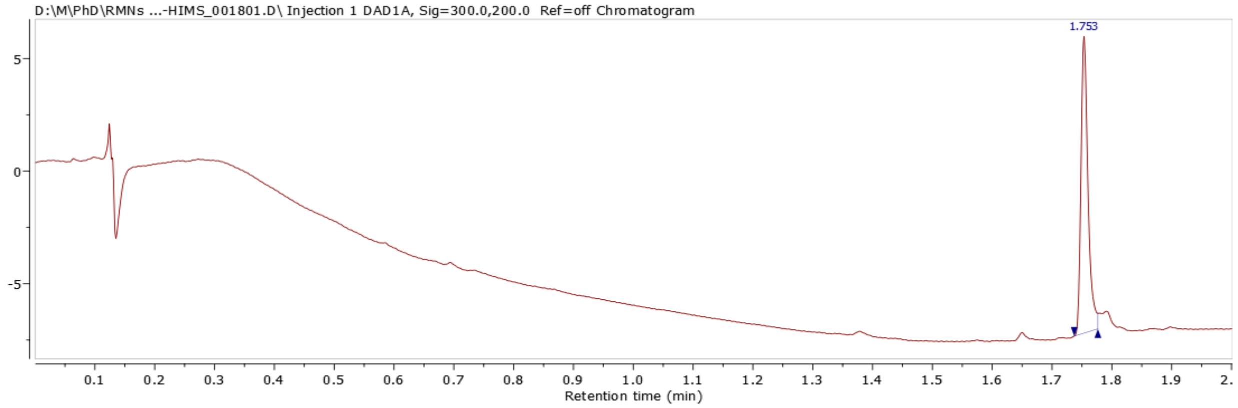

MS Detection range: 200-800 Da; MW of compound 40 = 881.02 Da

^{1}H NMR – Chloroform-*d*

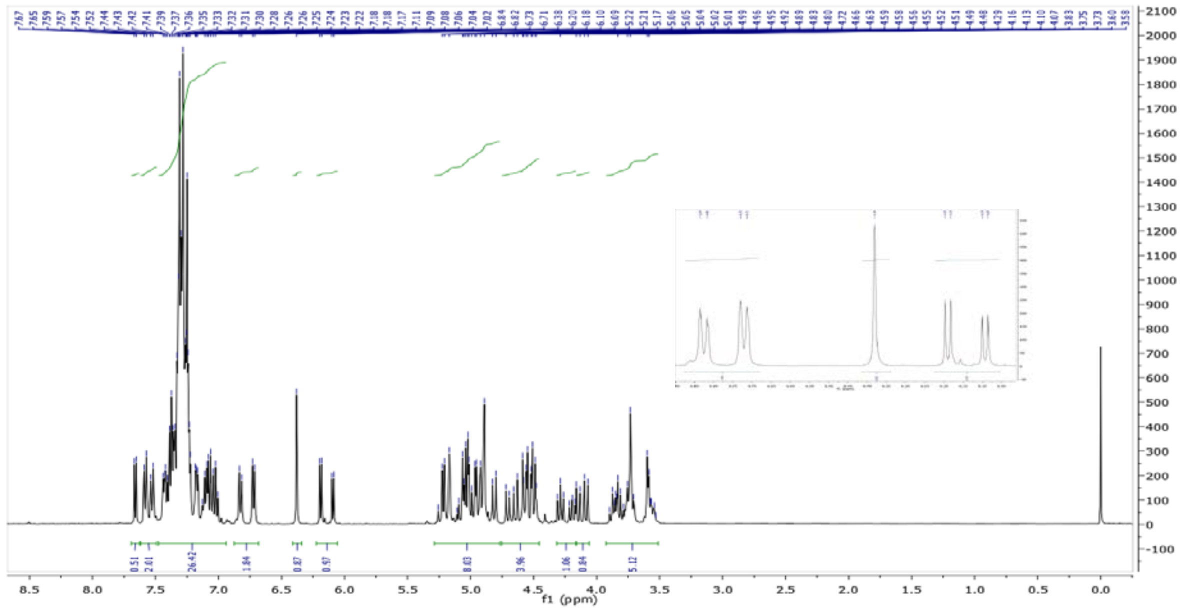

^{13}C NMR – Chloroform-*d*

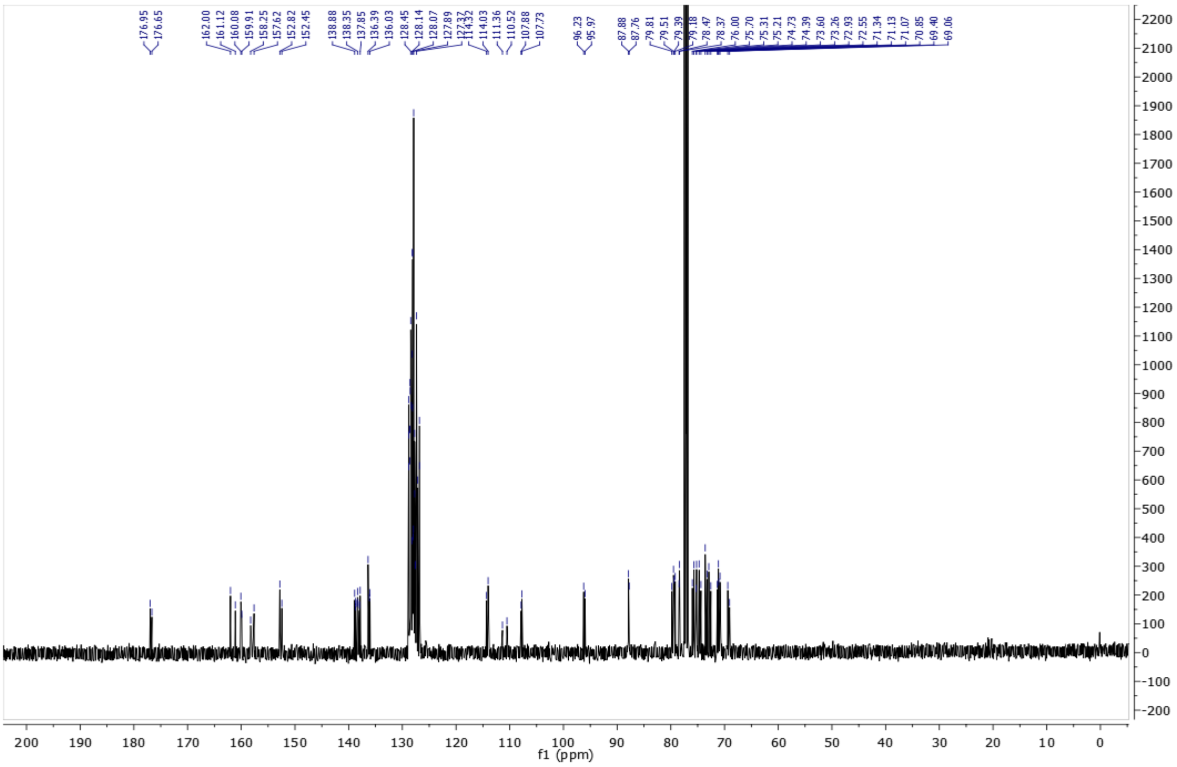

NMR spectra of final compounds ***4-12****, and* ***15-22*** *(solvent used to run spectra is indicated)*

2-Cyclopropyl-5,7-dihydroxy-4*H*-chromen-4-one (4)

^{1}H NMR – MeOD

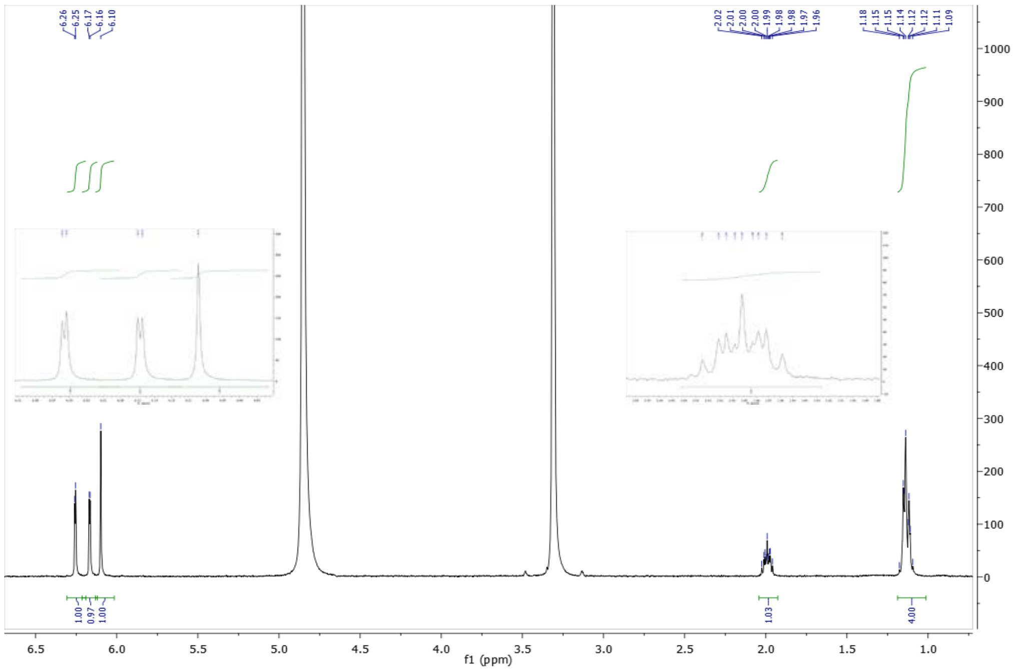

^{13}C NMR – MeOD

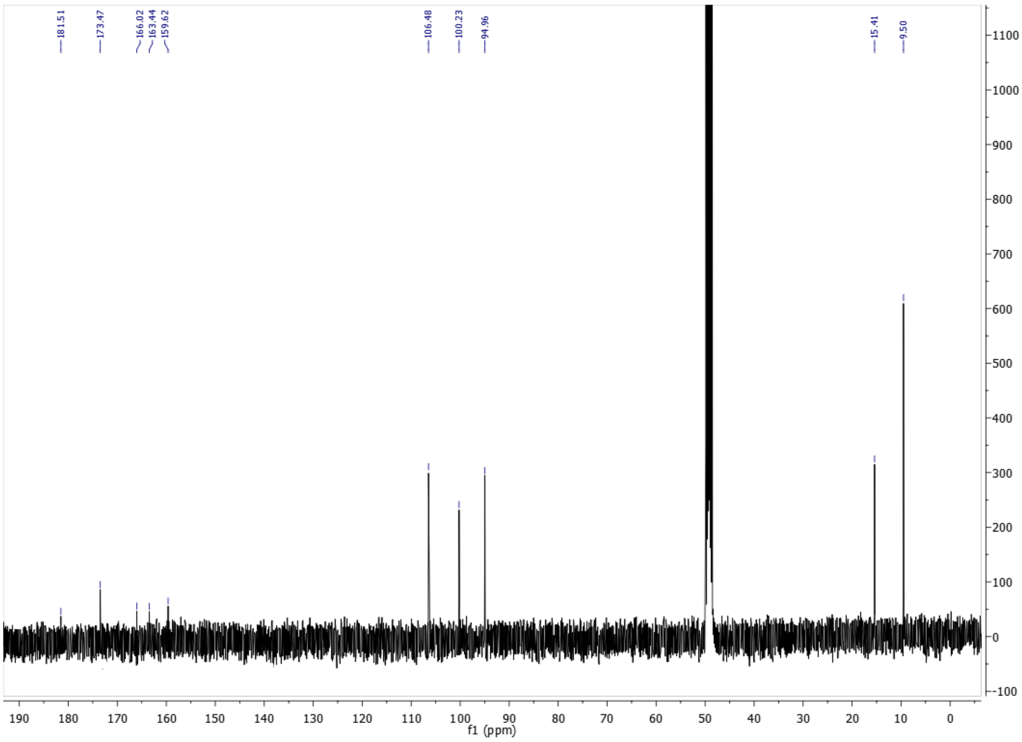

2-Cyclobutyl-5,7-dihydroxy-4*H*-chromen-4-one (5)

^{1}H NMR – MeOD

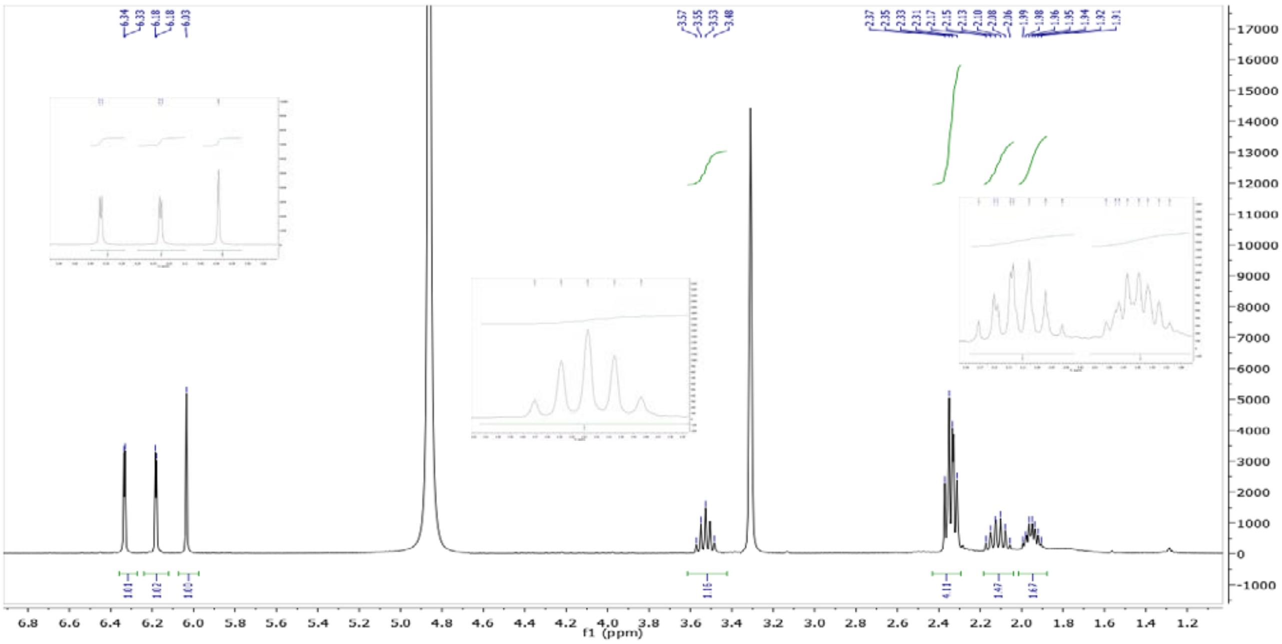

^{13}C NMR – MeOD

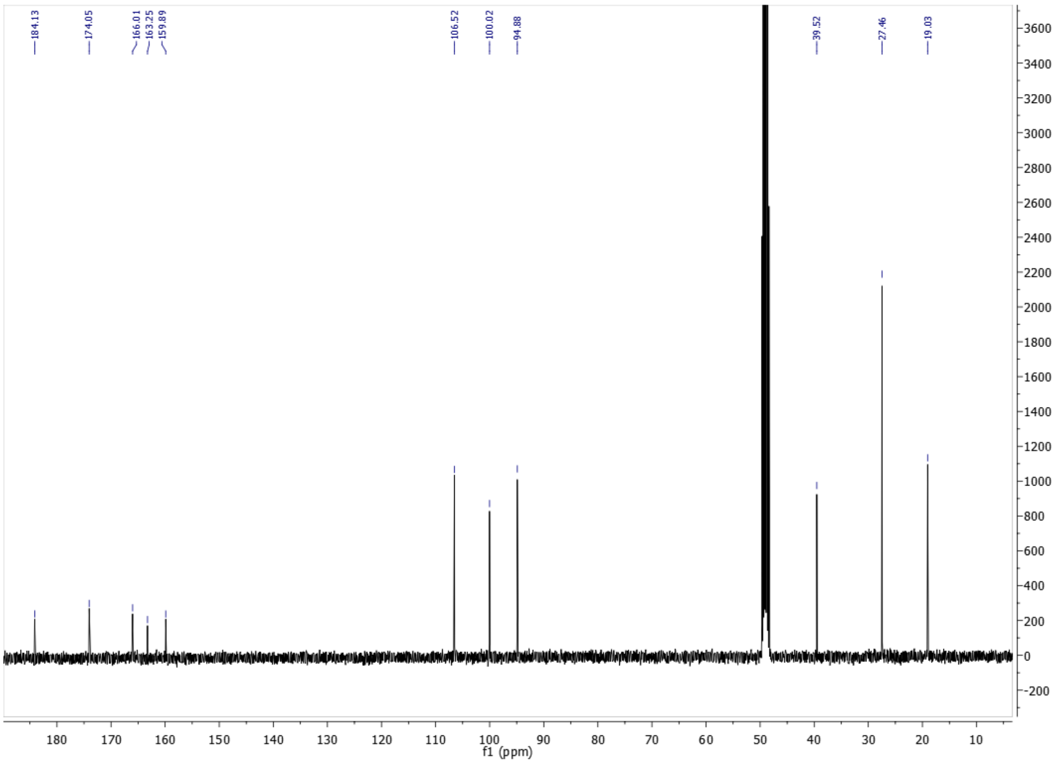

5,7-Dihydroxy-2-(1-methylpropyl)-4*H*-chromen-4-one (6)

^{1}H NMR – MeOD

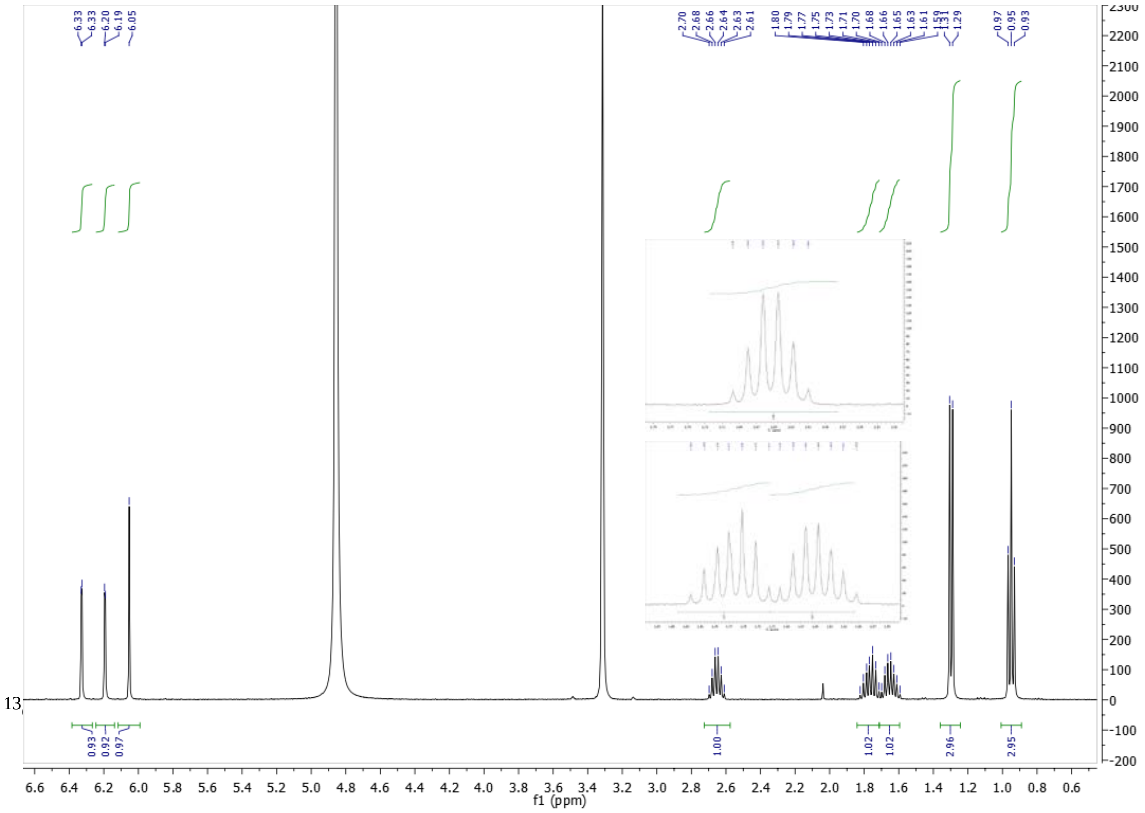

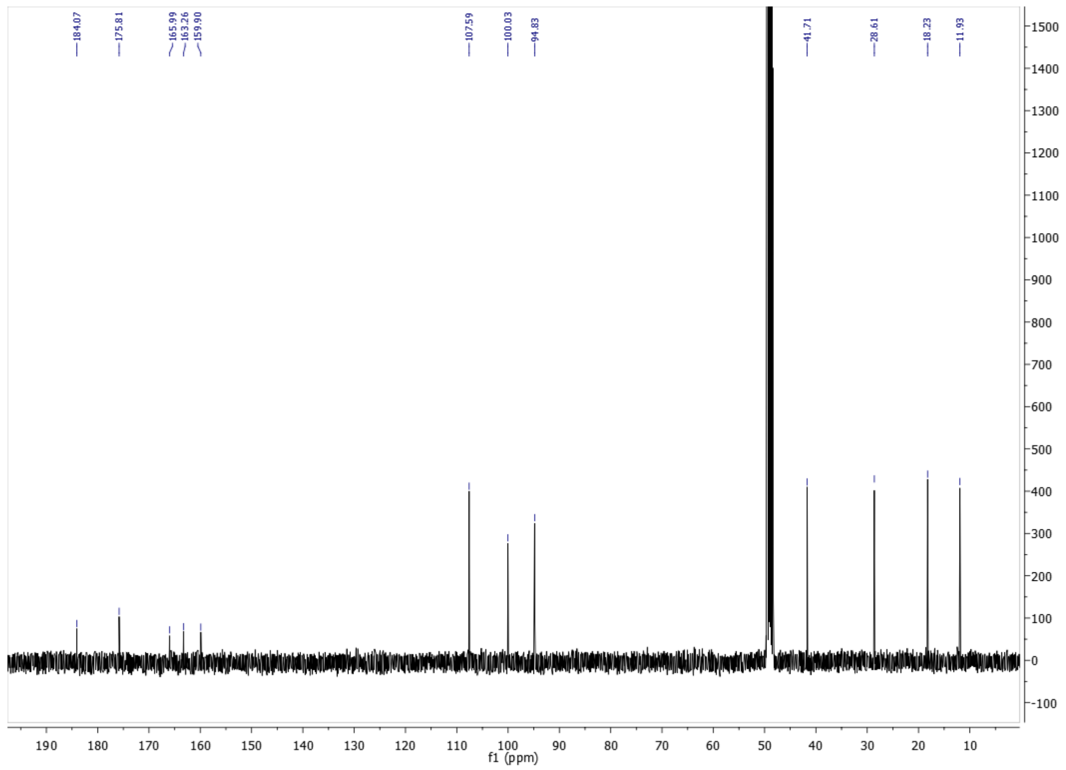
184.07
175.81
165.99
163.26
159.90
107.59
100.03
94.83
41.71
28.61
18.23
11.93
f1 (ppm)

HMBC

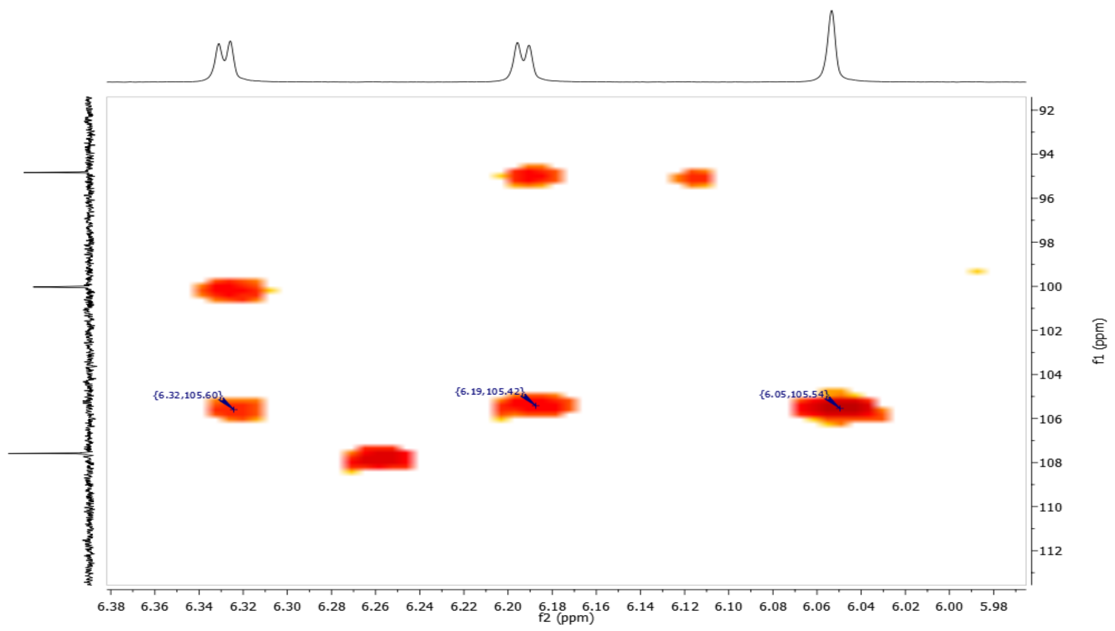
{6.32,105.60}
{6.19,105.42}
{6.05,105.54}
f2 (ppm)
f1 (ppm)

4´-Fluoro-5,7-dihydroxyflavone (7)

^{1}H NMR – MeOD

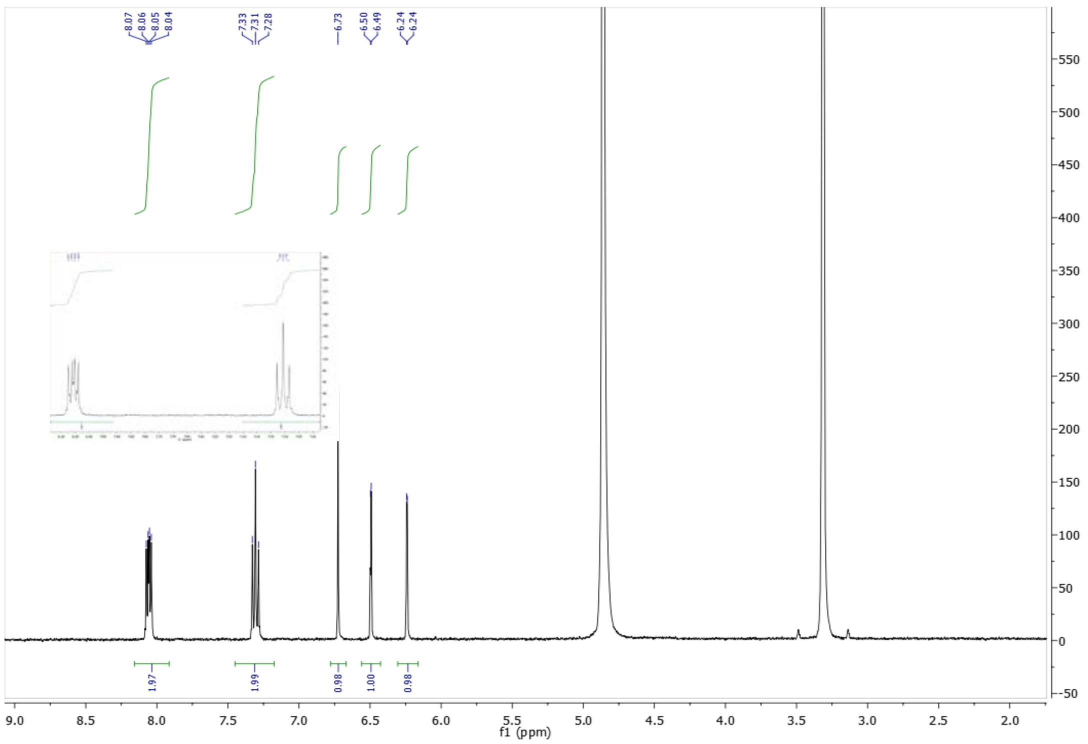

^{13}C NMR – MeOD

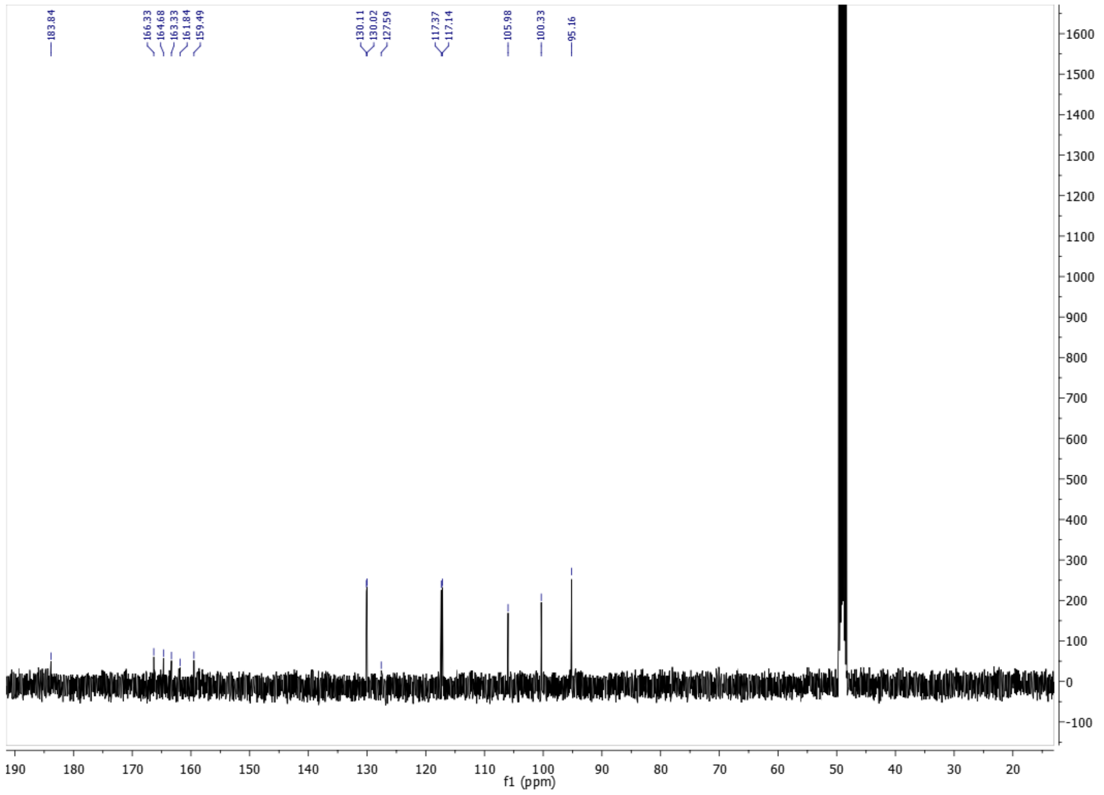

5,7-Dihydroxy-4´-(morpholin-4-yl)flavone (8)

^{1}H NMR – MeOD

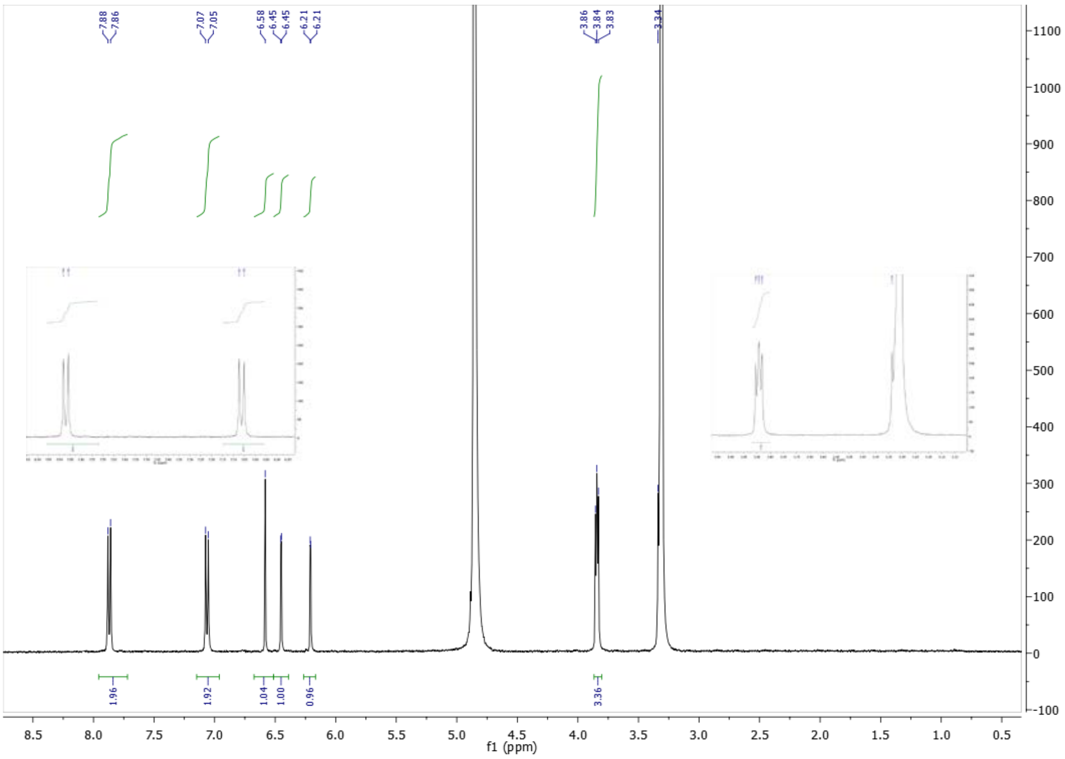

^{13}C NMR – MeOD

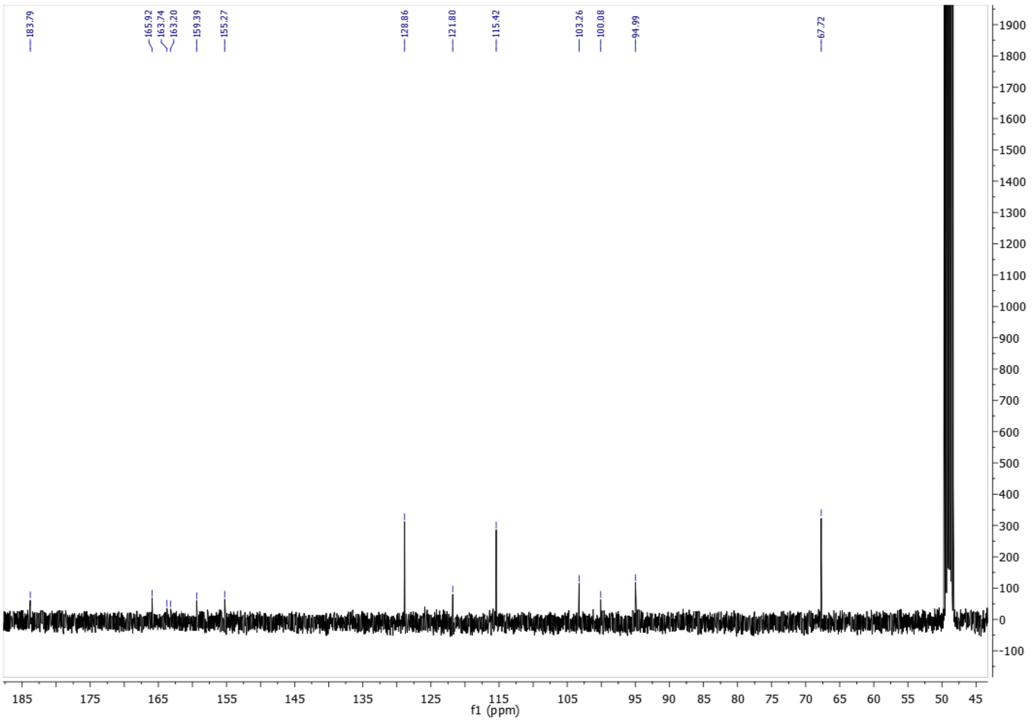

HMBC

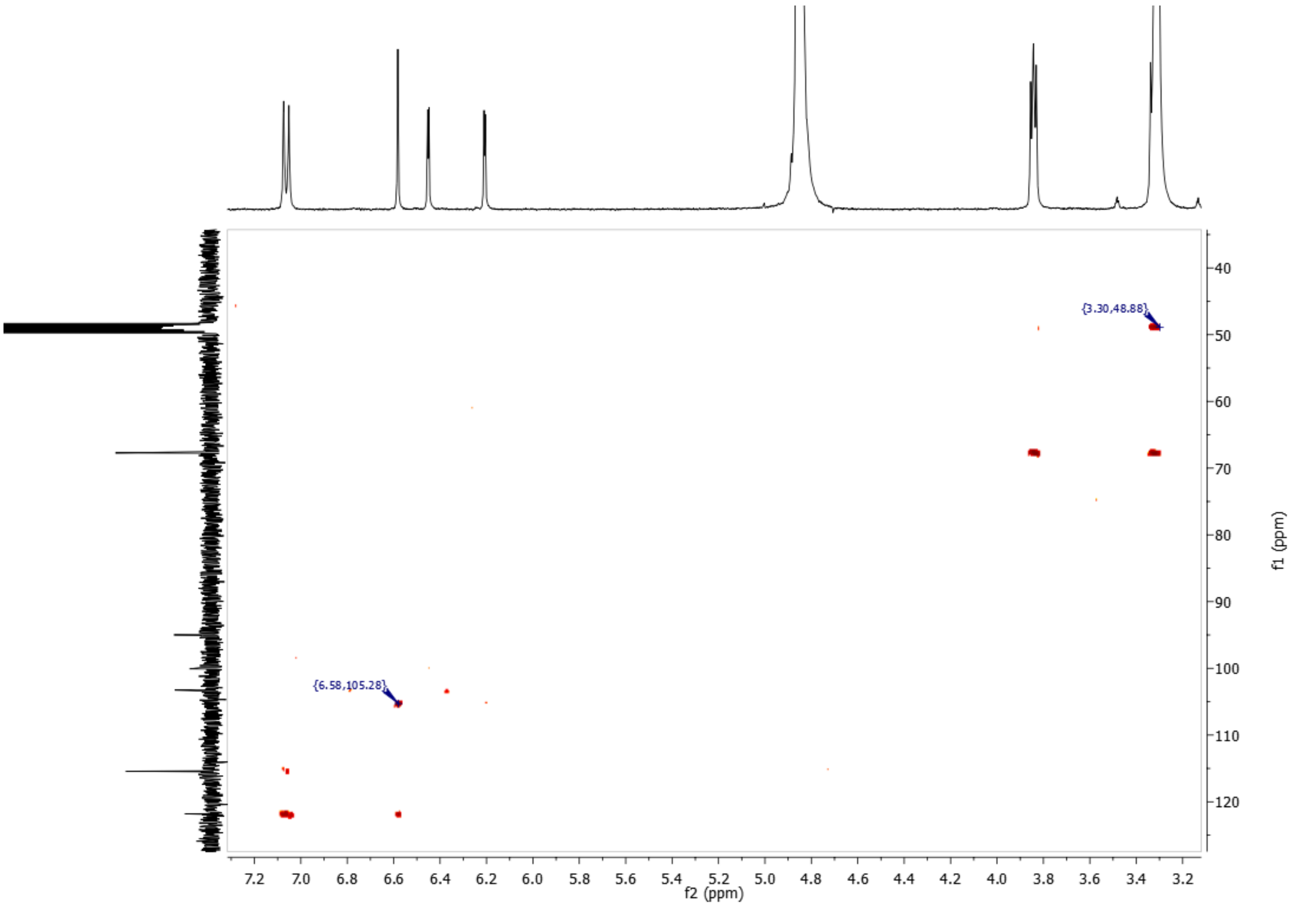

5,7-Dihydroxy-4′-(pyrrolidin-1-yl)flavone (9)

^{1}H NMR – DMSO-d_6

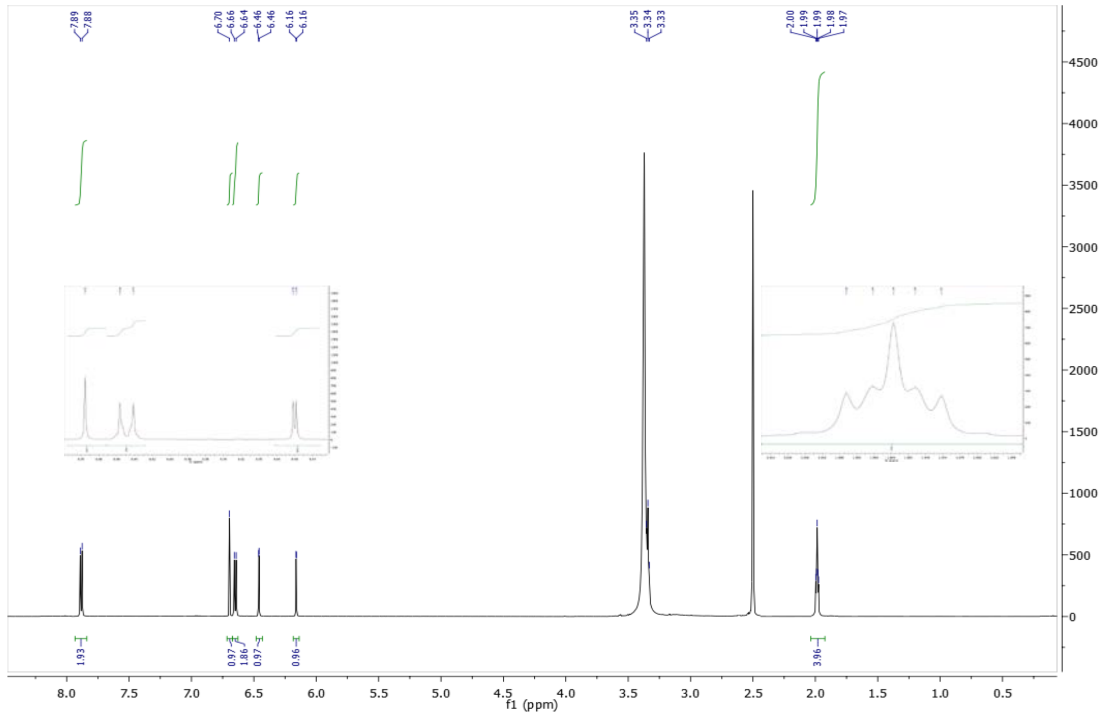

^{13}C NMR –DMSO-d_6

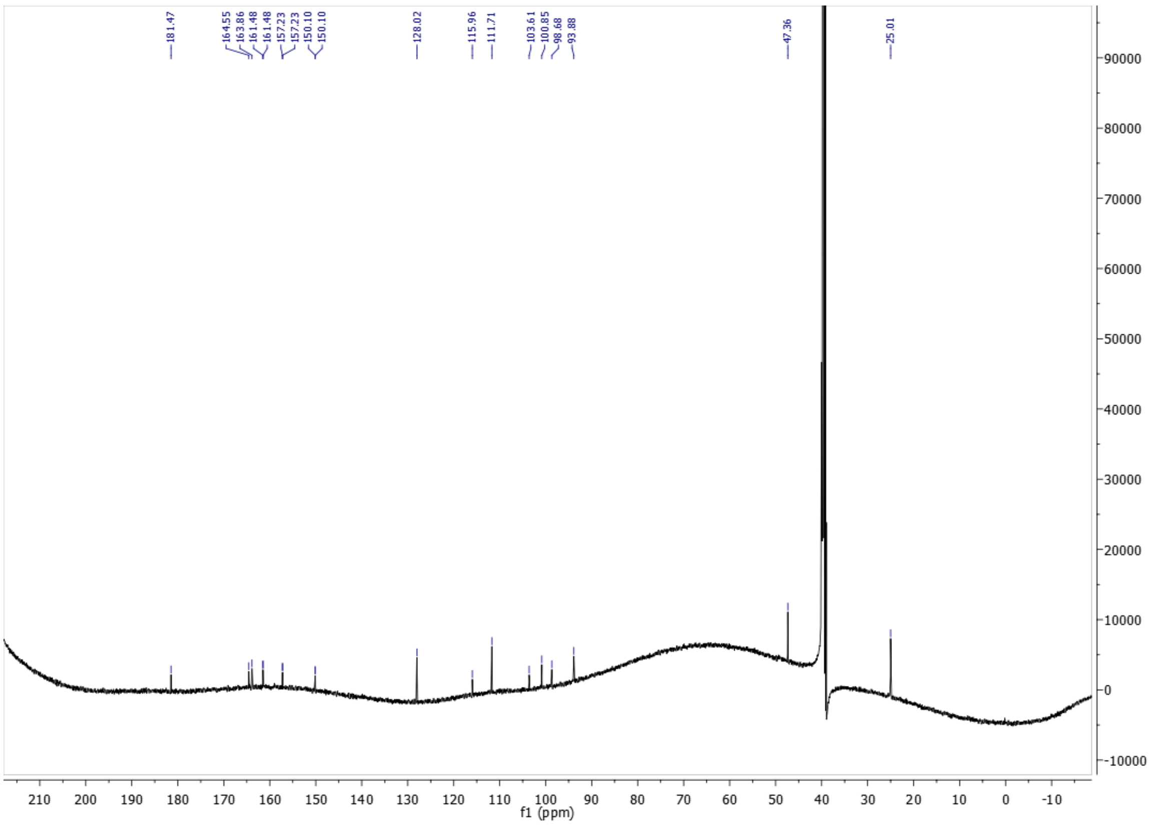

4′-Dimethylamino-5,7-dihydroxyflavone (10)

^{1}H NMR –DMSO-d_6

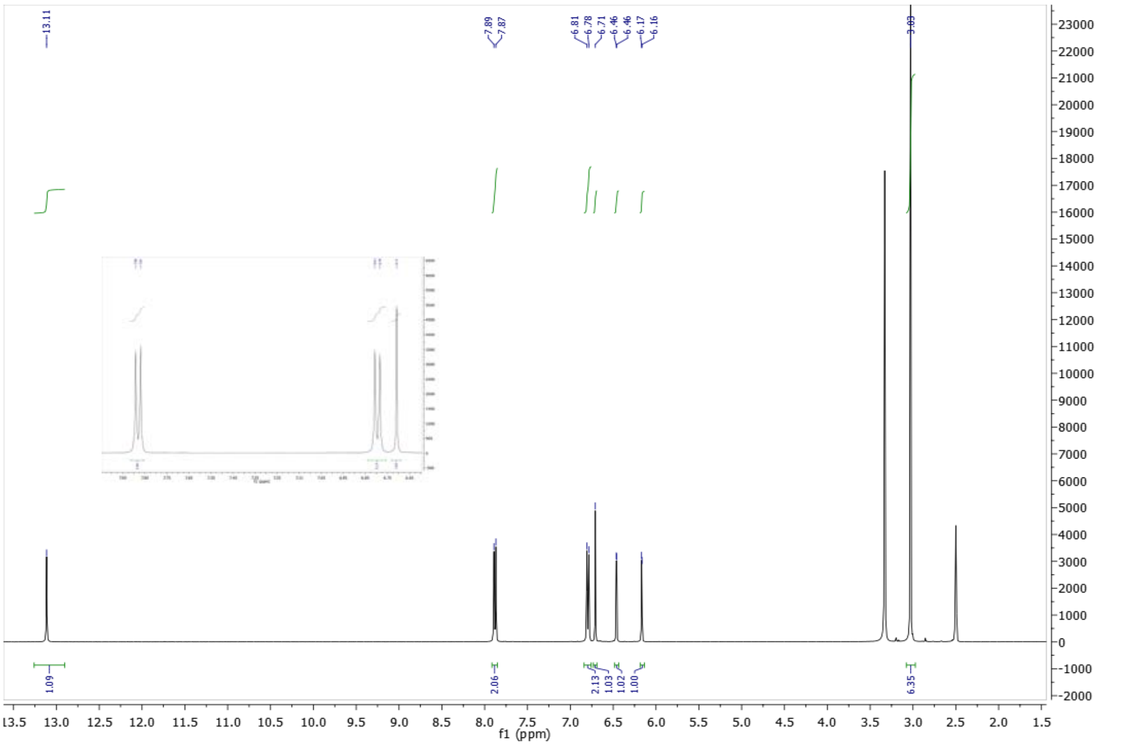

^{13}C NMR– DMSO-d_6

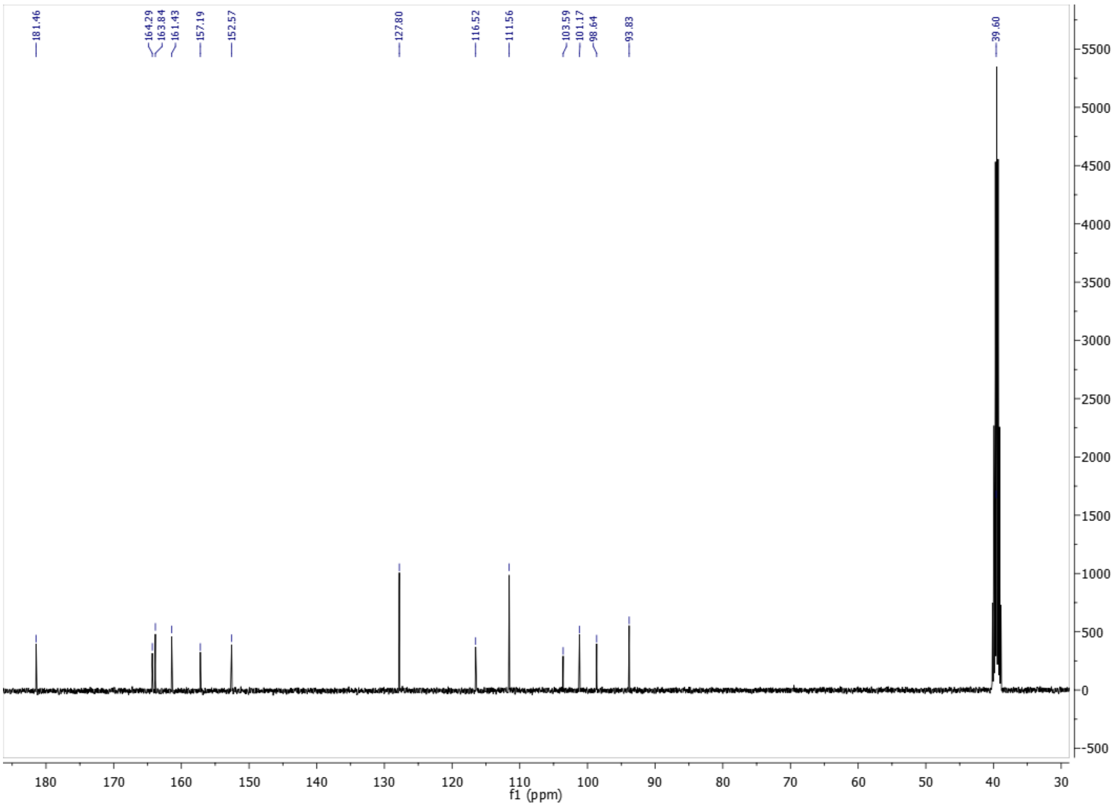

2-(Furan-2-yl)-5,7-dihydroxy-4*H*-chromen-4-one (11)

^{1}H NMR – Acetone-d_6

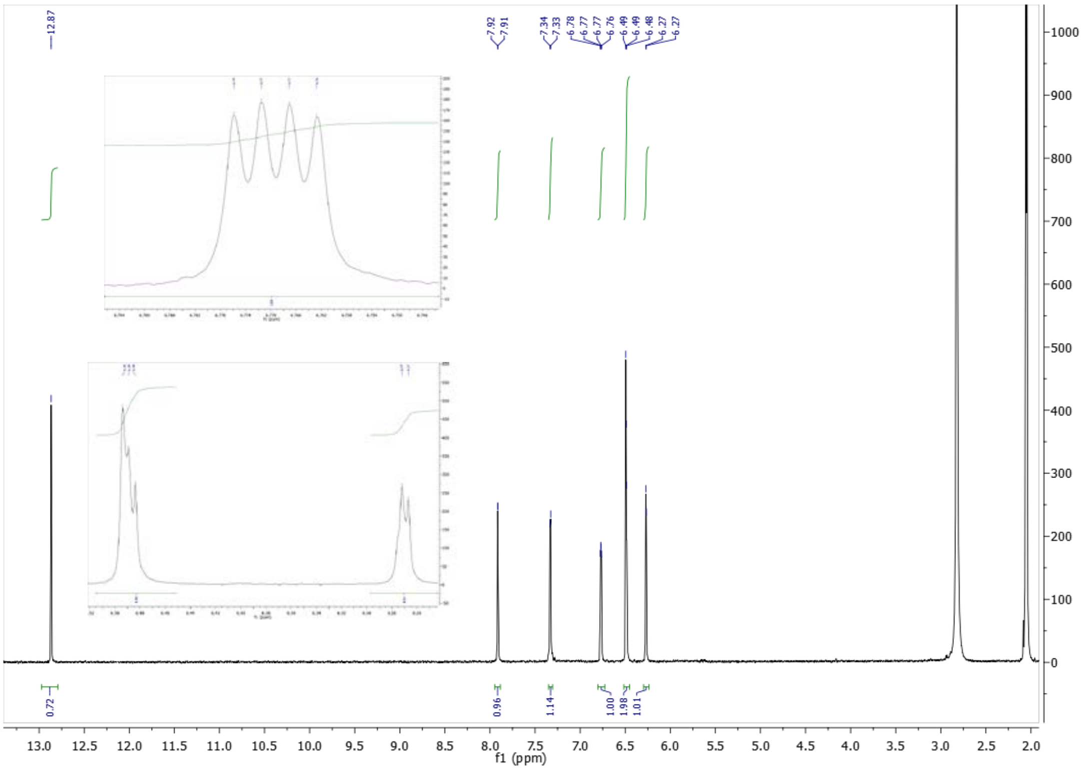

^{13}C NMR – Acetone-d_6

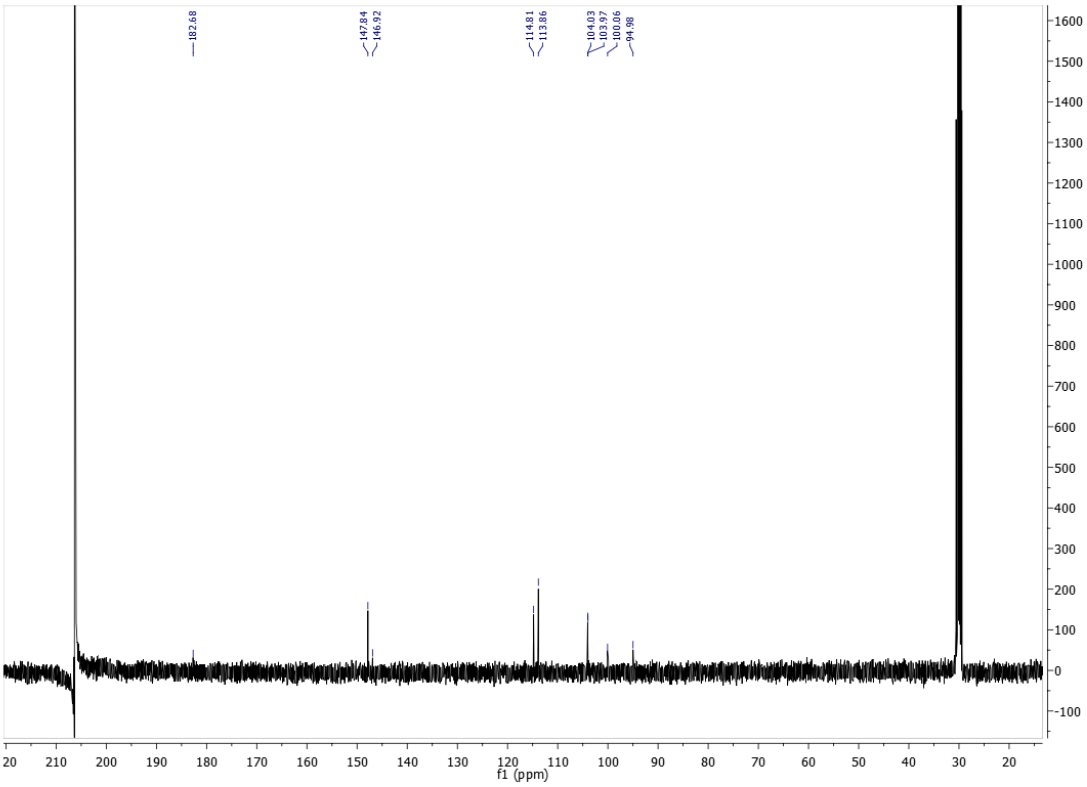

HMBC

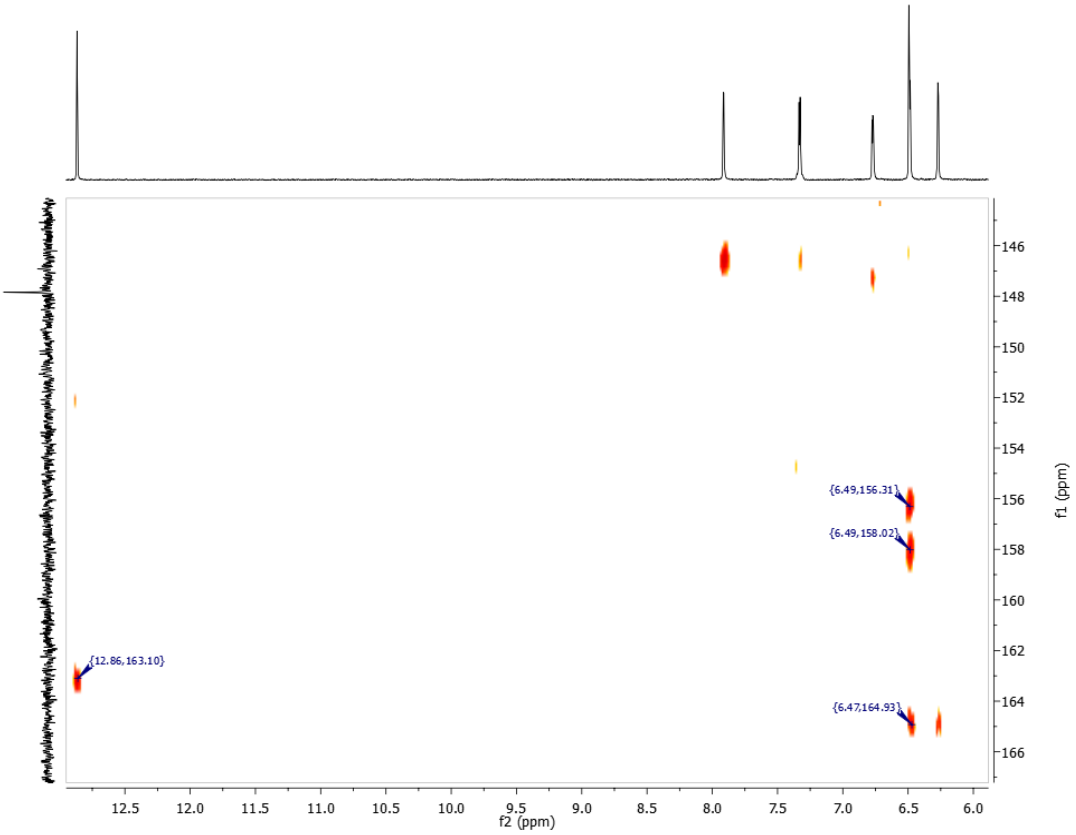

5,7-Dihydroxy-2-(pyridin-4-yl)-4*H*-chromen-4-one (12)

^{1}H NMR – MeOD

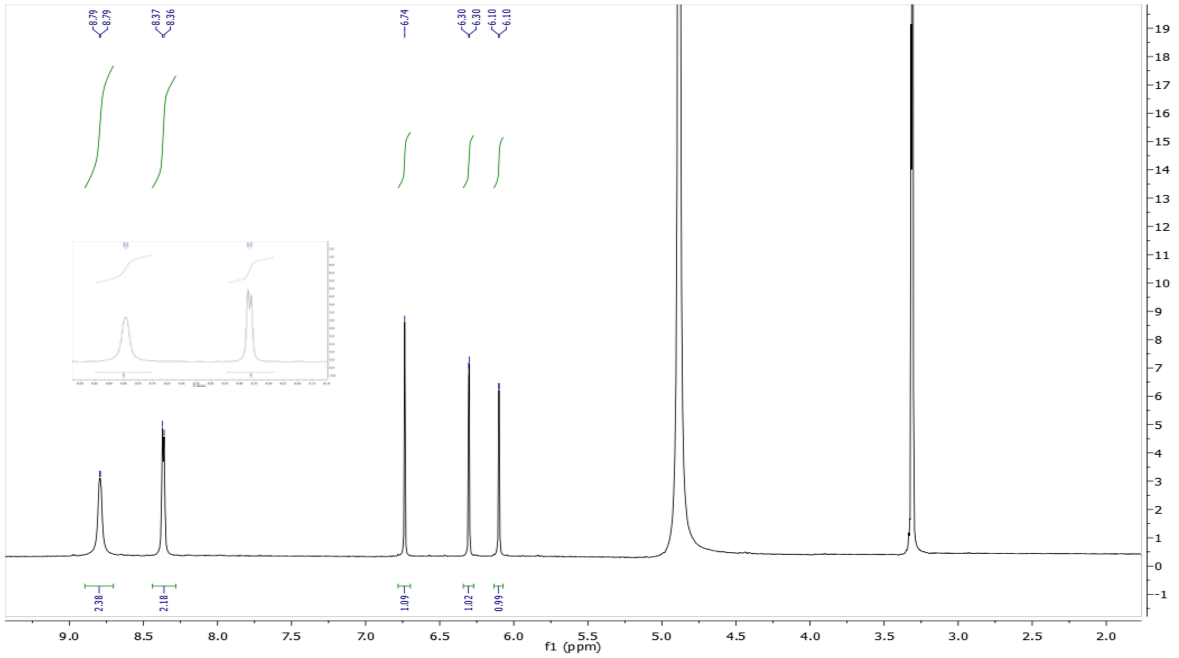

^{13}C NMR – MeOD

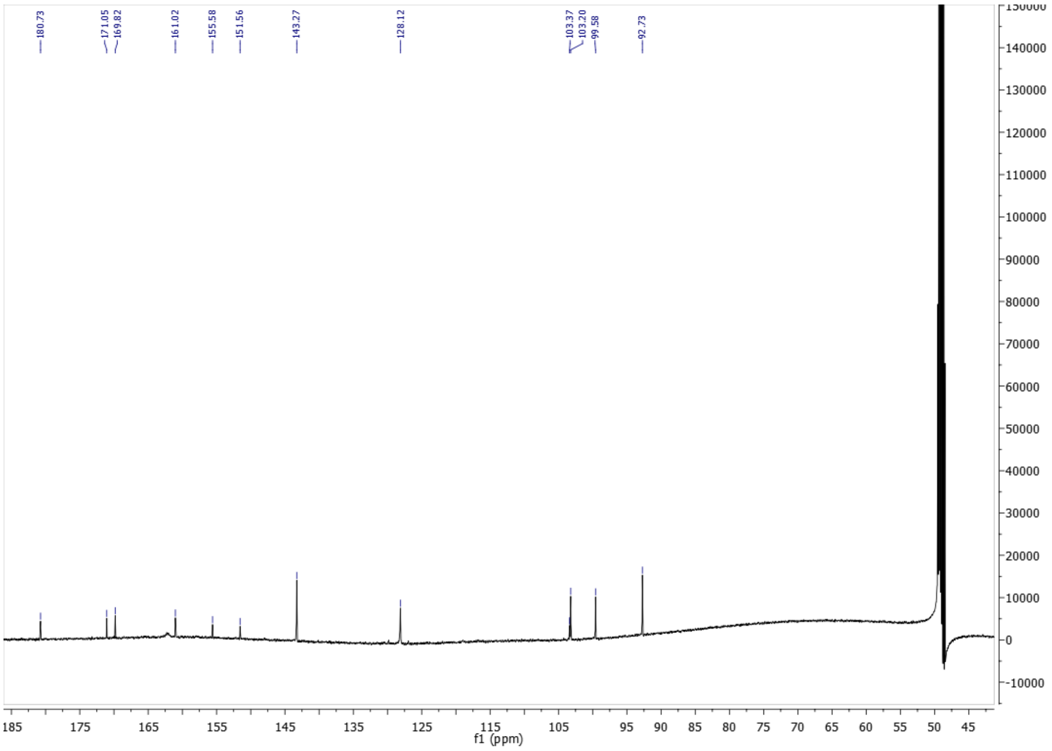

2-Cyclobutyl-5,7-dihydroxy-4*H*-chromen-4-one (5)

^{1}H NMR – MeOD

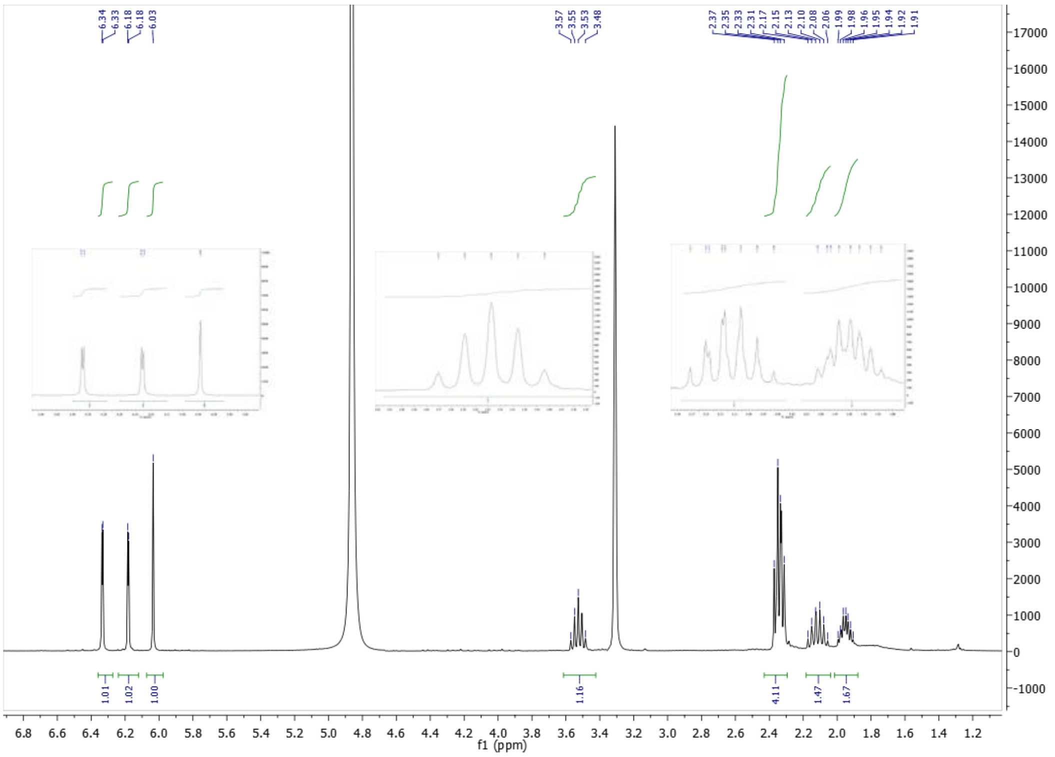

^{13}C NMR – MeOD

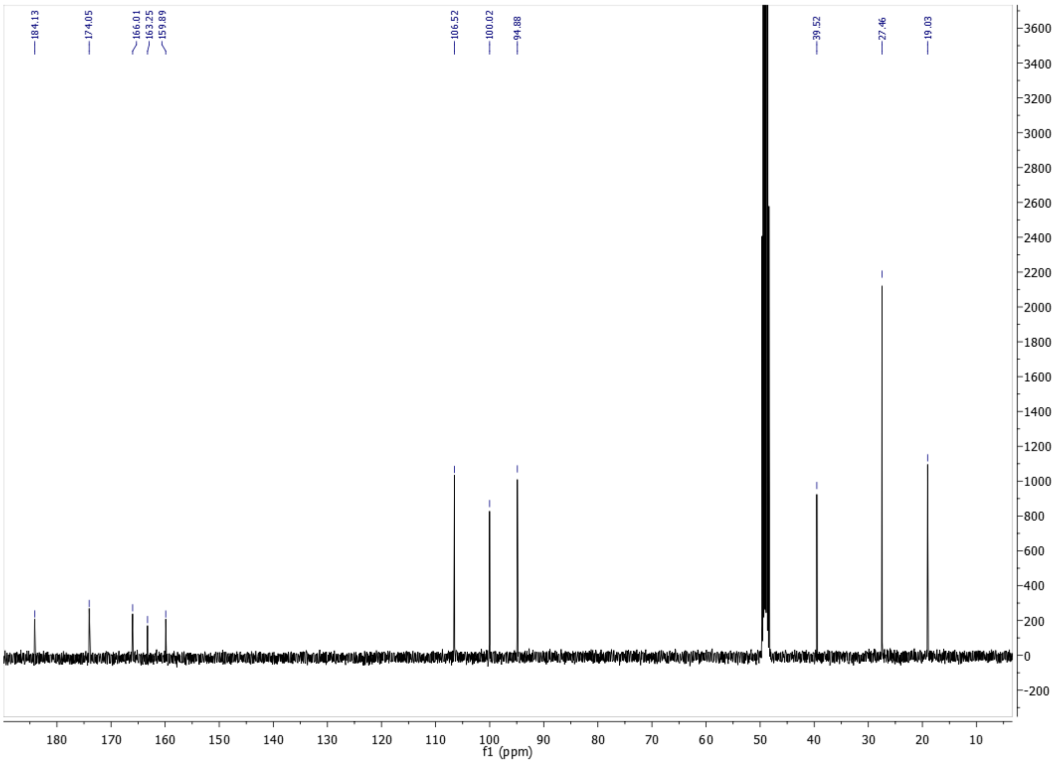

2-Cyclopropyl-8-(β-D-glucopyranosyl)-5,7-dihydroxy-4*H*-chromen-4-one (15)

^{1}H NMR - MeOD

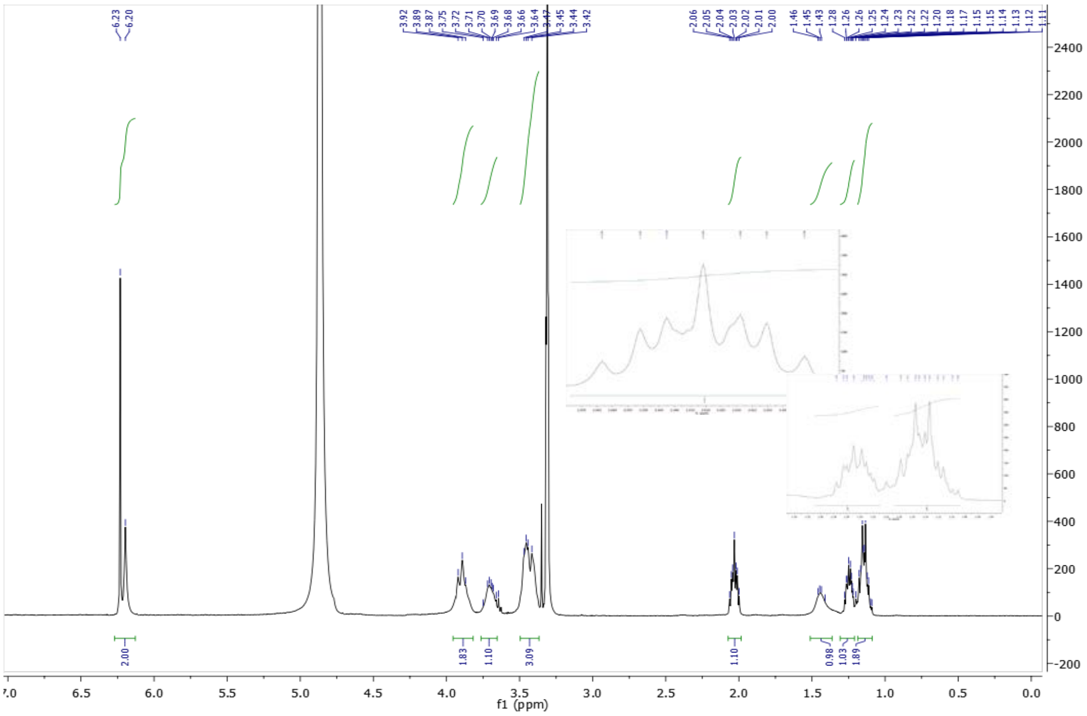

^{13}C NMR - MeOD

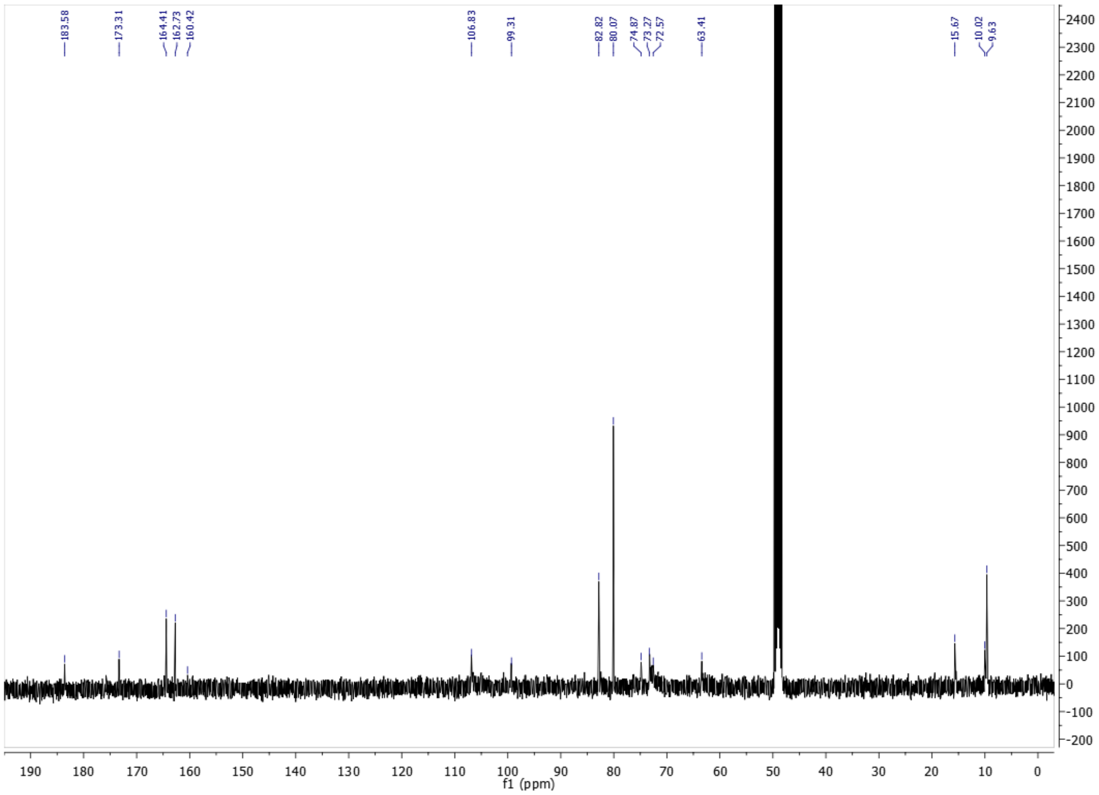

HMBC

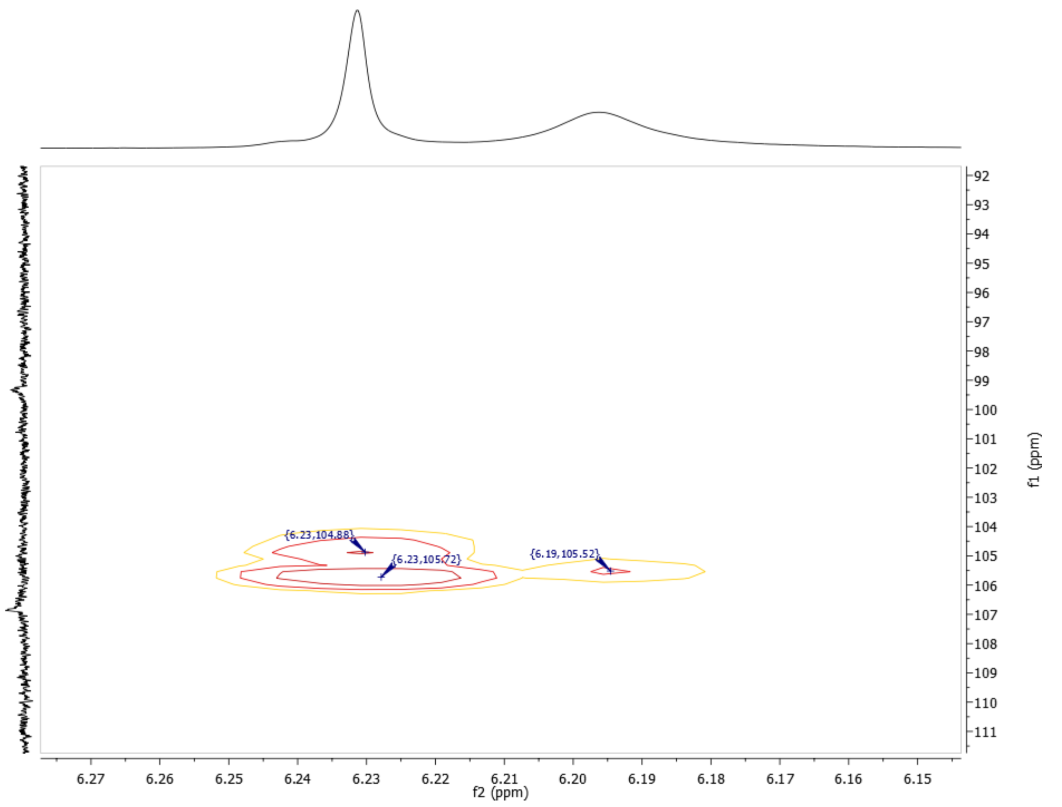

8-(β-D-Glucopyranosyl)-5,7-dihydroxyflavone (16)

^{1}H NMR - MeOD

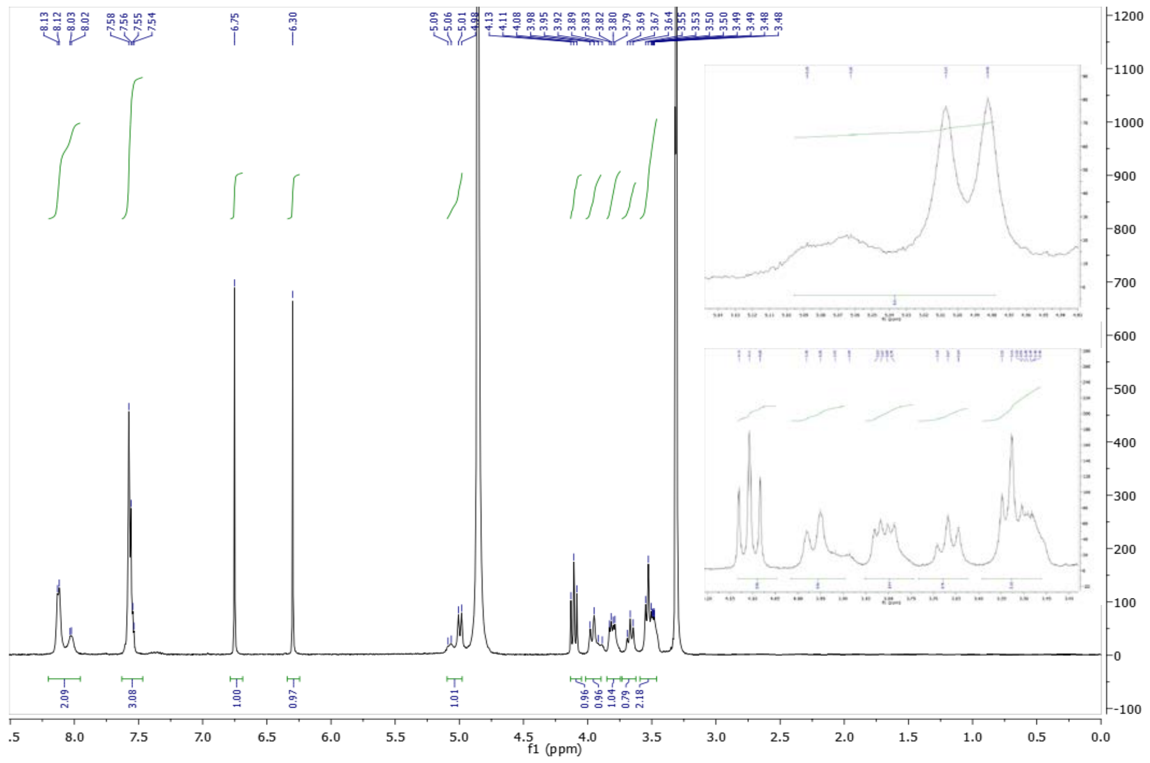

^{13}C NMR - MeOD

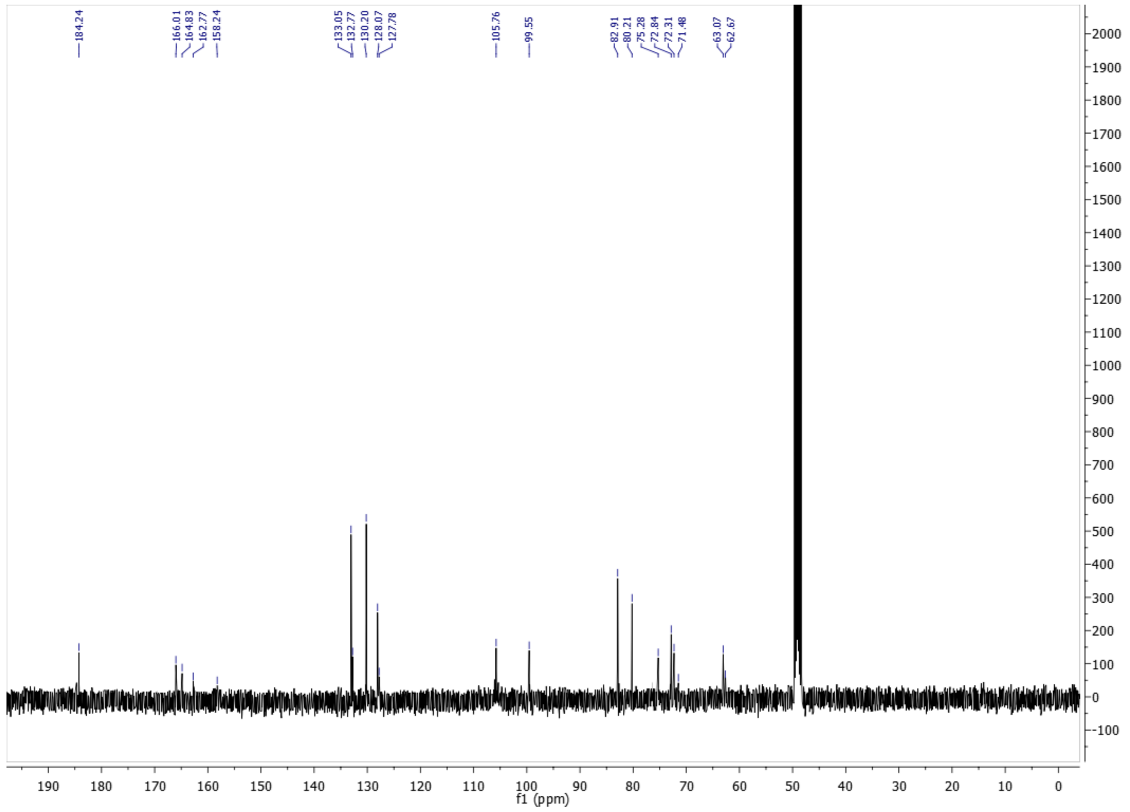

HMBC

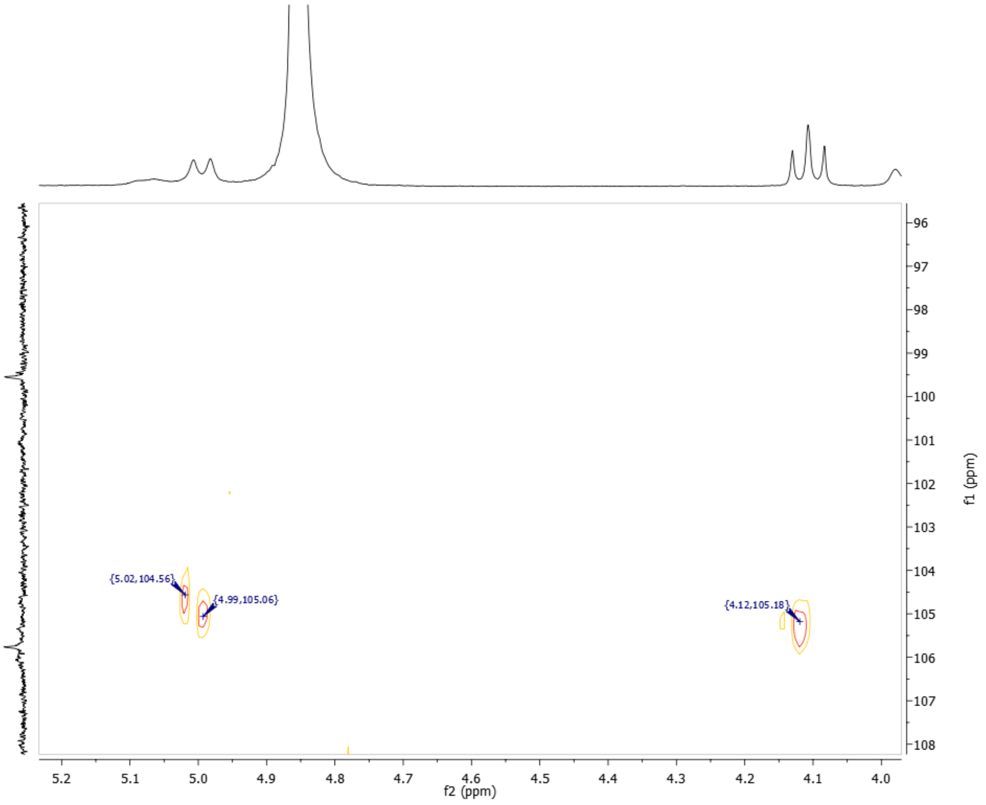

5,7-Dihydroxy-4´-(pyrrolidin-1-yl)flavone (17)

^{1}H NMR – DMSO-d_6

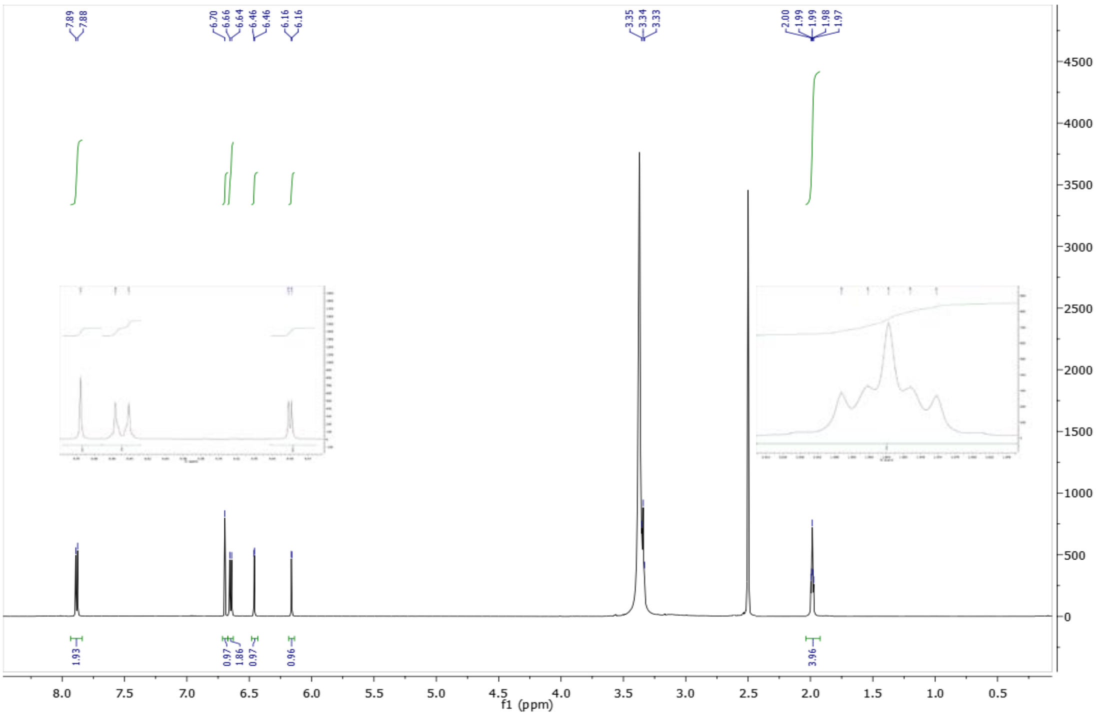

^{13}C NMR –DMSO-d_6

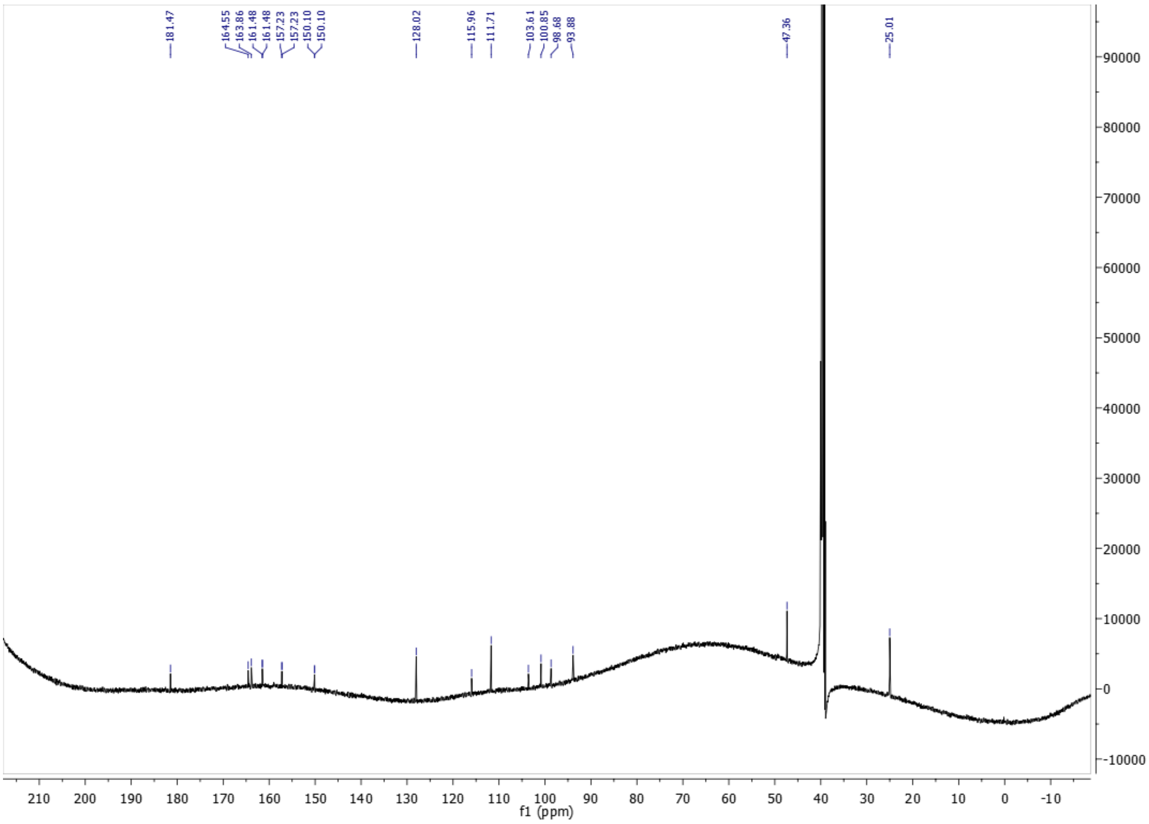

4´-Fluoro-8-(β-D-glucopyranosyl)-5,7-dihydroxyflavone (18)

^{1}H NMR - MeOD

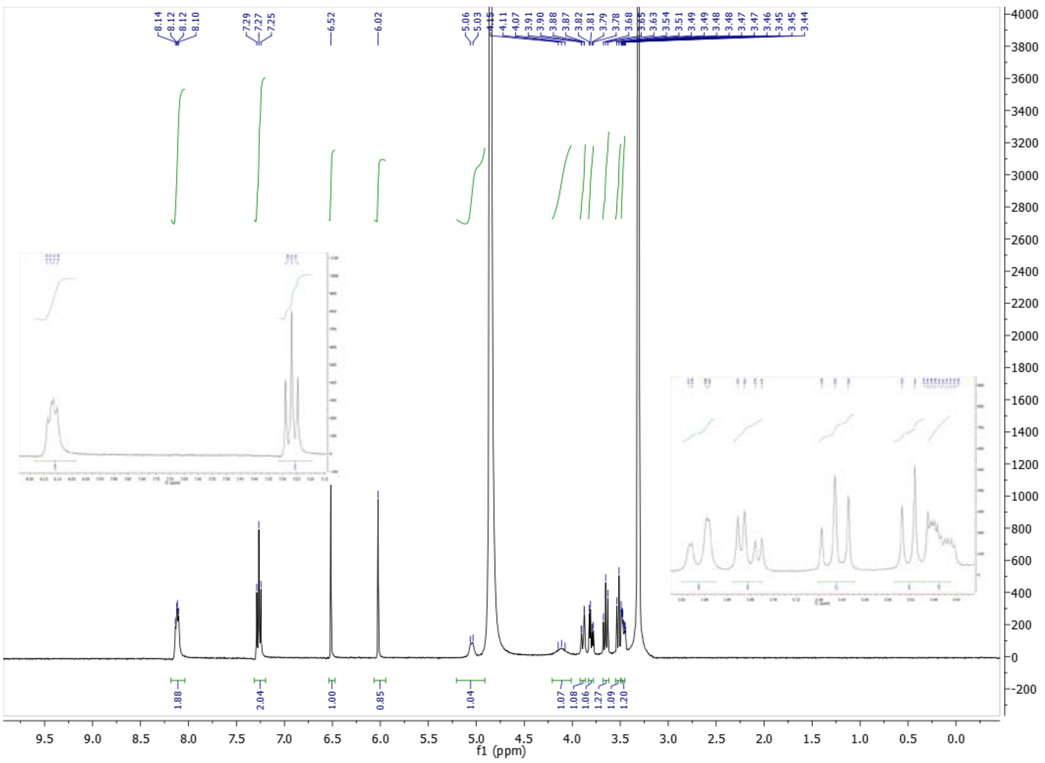

^{13}C NMR - MeOD

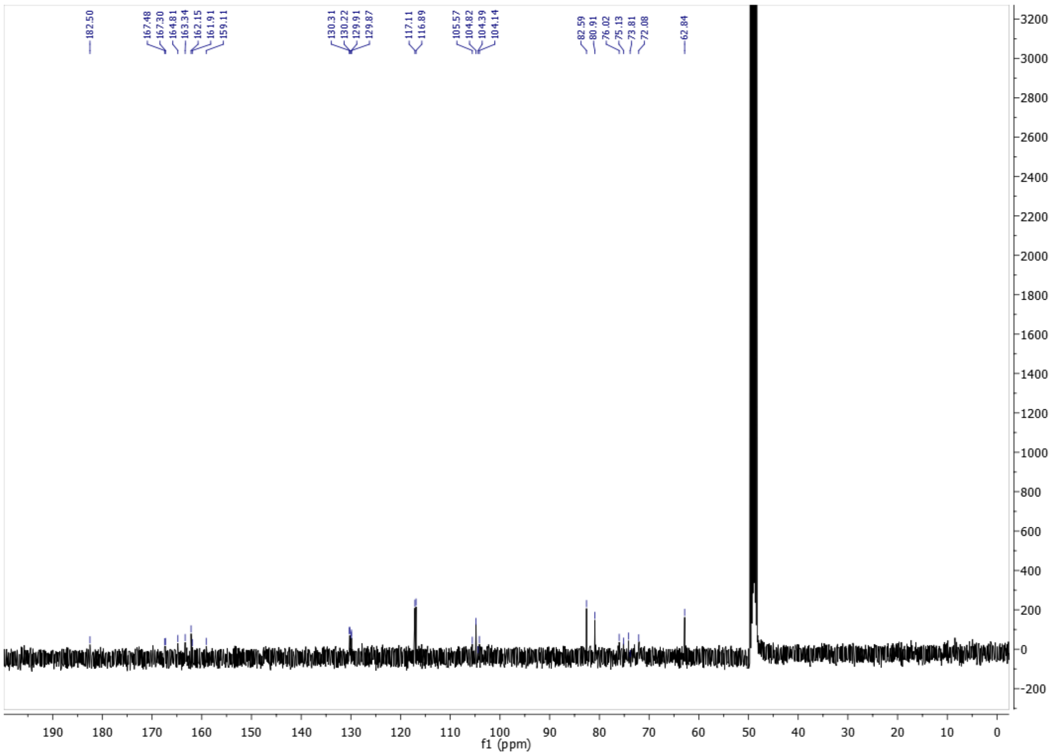

8-(β-D-Glucopyranosyl)-5,7-dihydroxy-4´-(morpholin-4-yl)flavone (19)

^{1}H NMR - MeOD

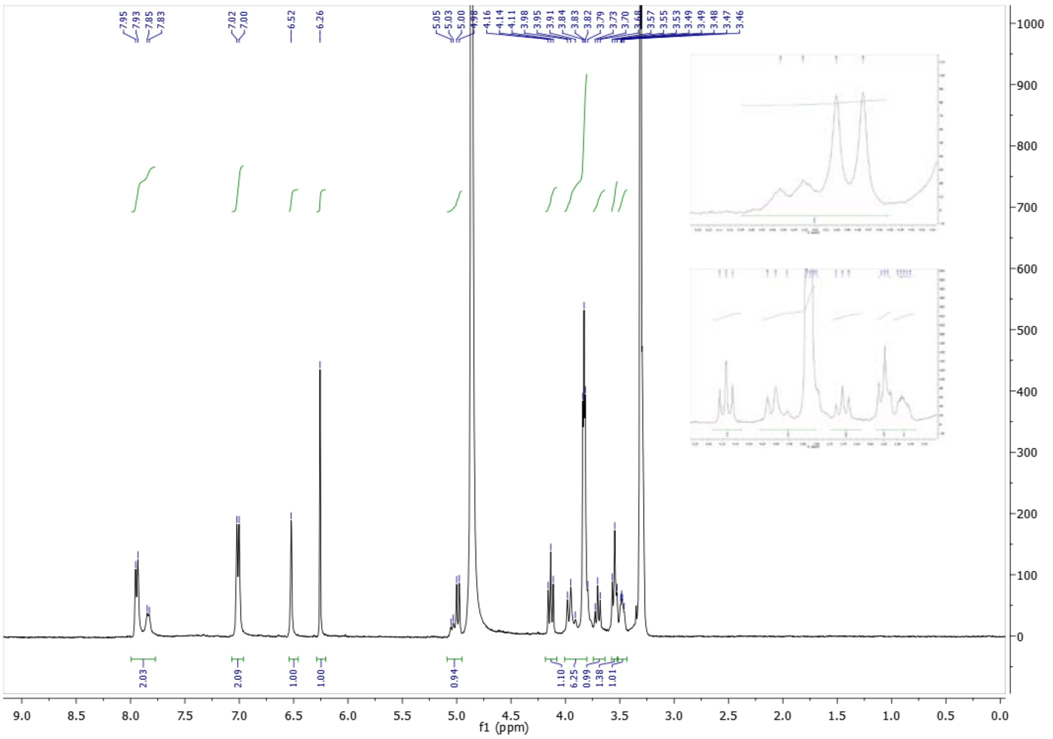

^{13}C NMR - MeOD

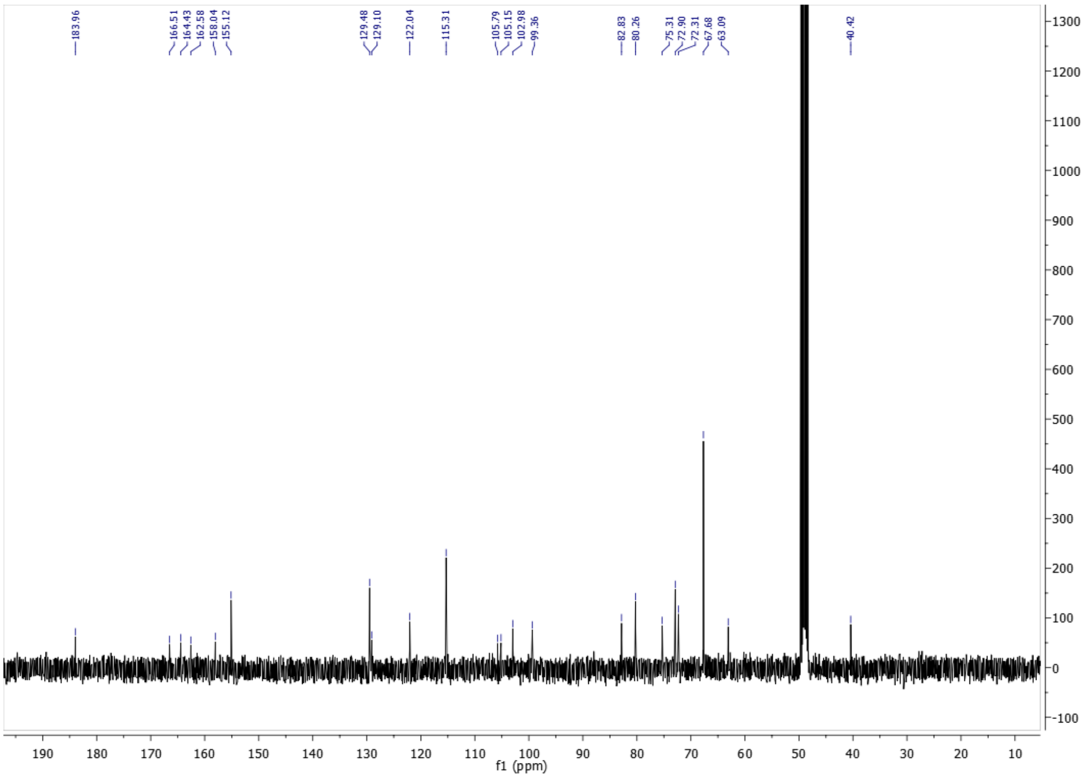

8-(β-D-Glucopyranosyl)-5,7-dihydroxy-4´-(pyrrolidin-1-yl)flavone (20)

^{1}H NMR - MeOD

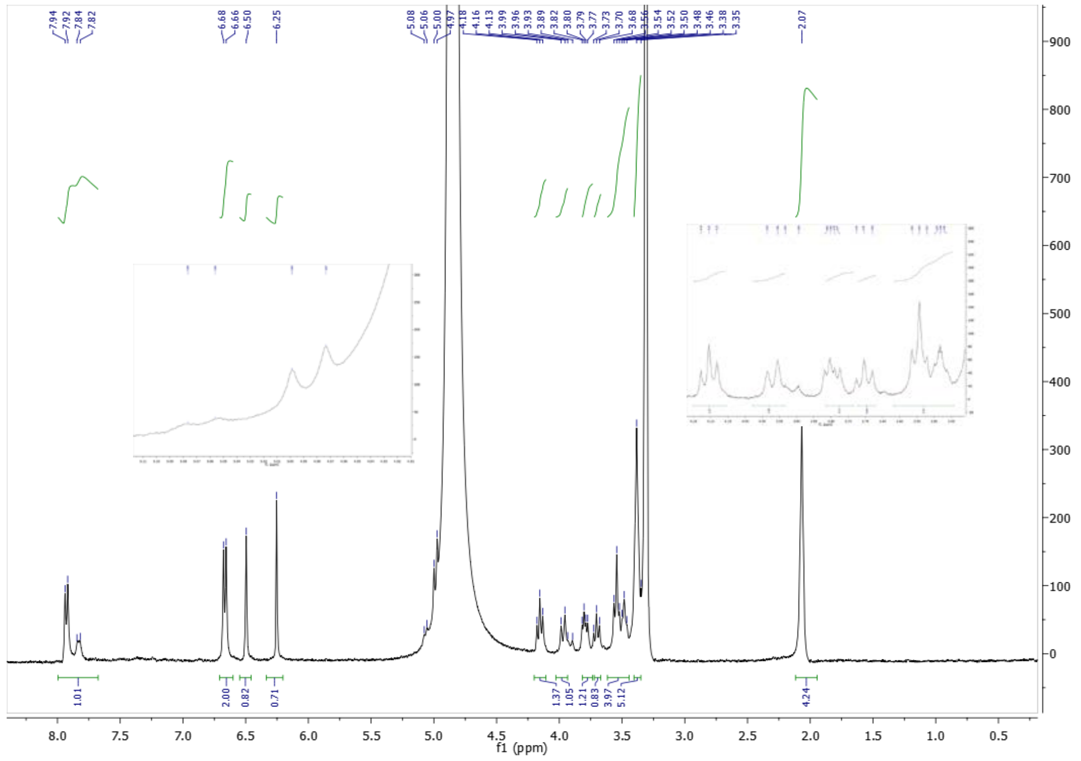

^{13}C NMR

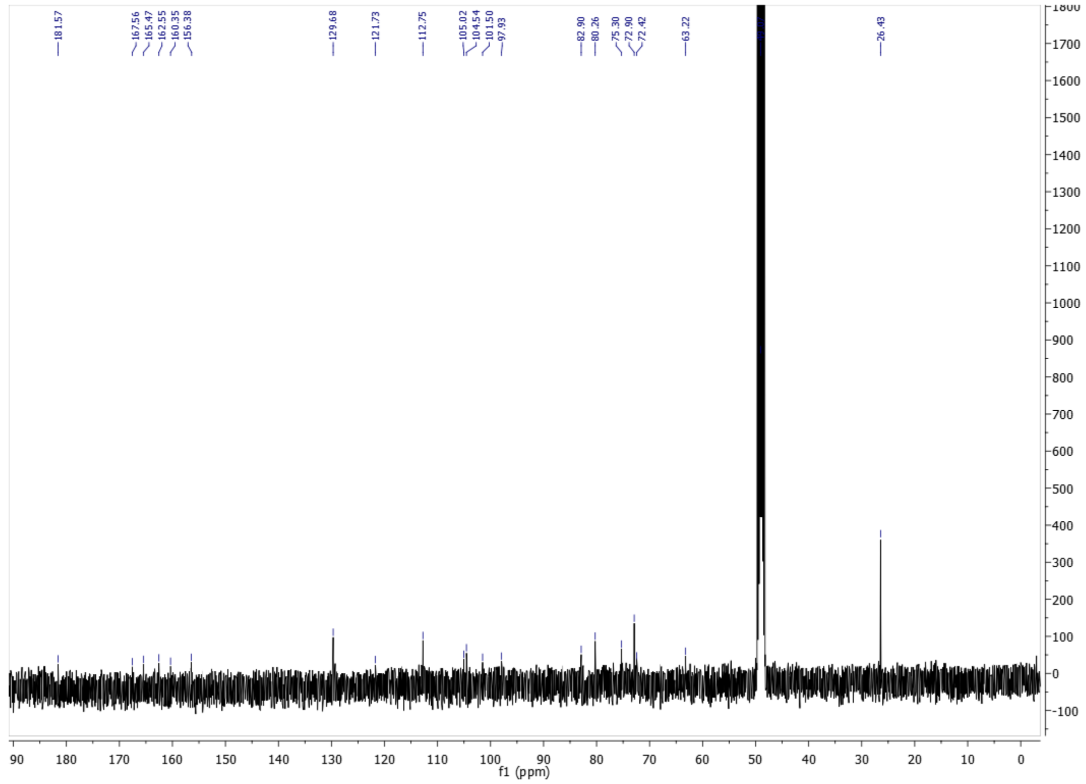

2-(Furan-2-yl)-8-(β-D-glucopyranosyl)-5,7-dihydroxy-4*H*-chromen-4-one (21)

^{1}H NMR - MeOD

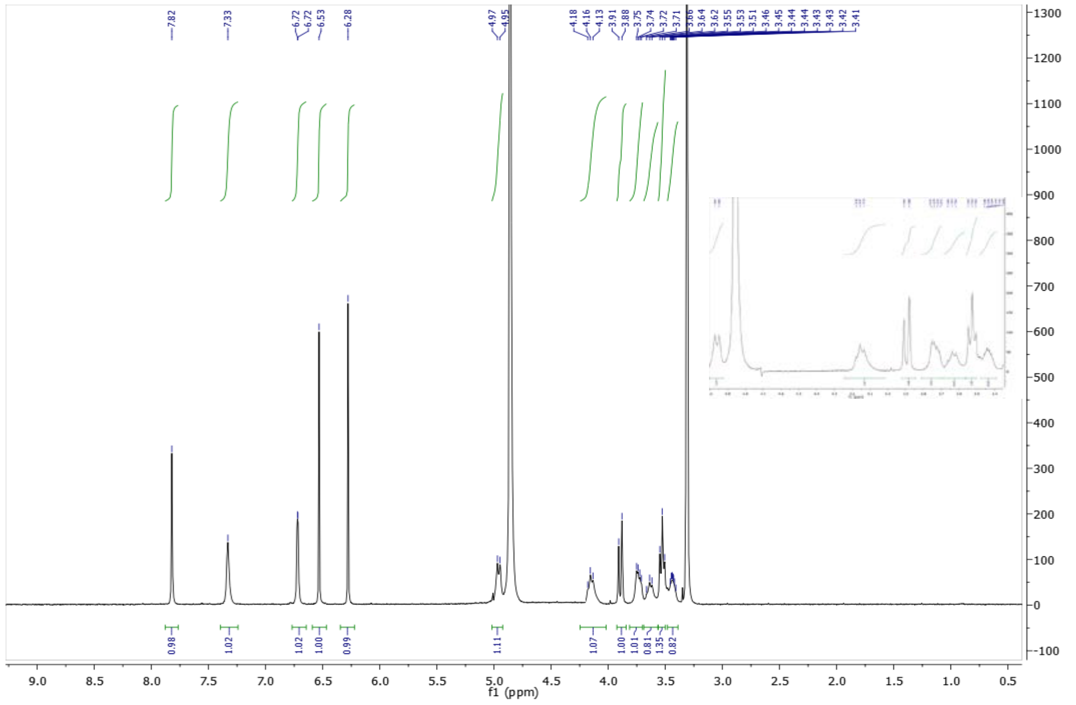

^{13}C NMR - MeOD

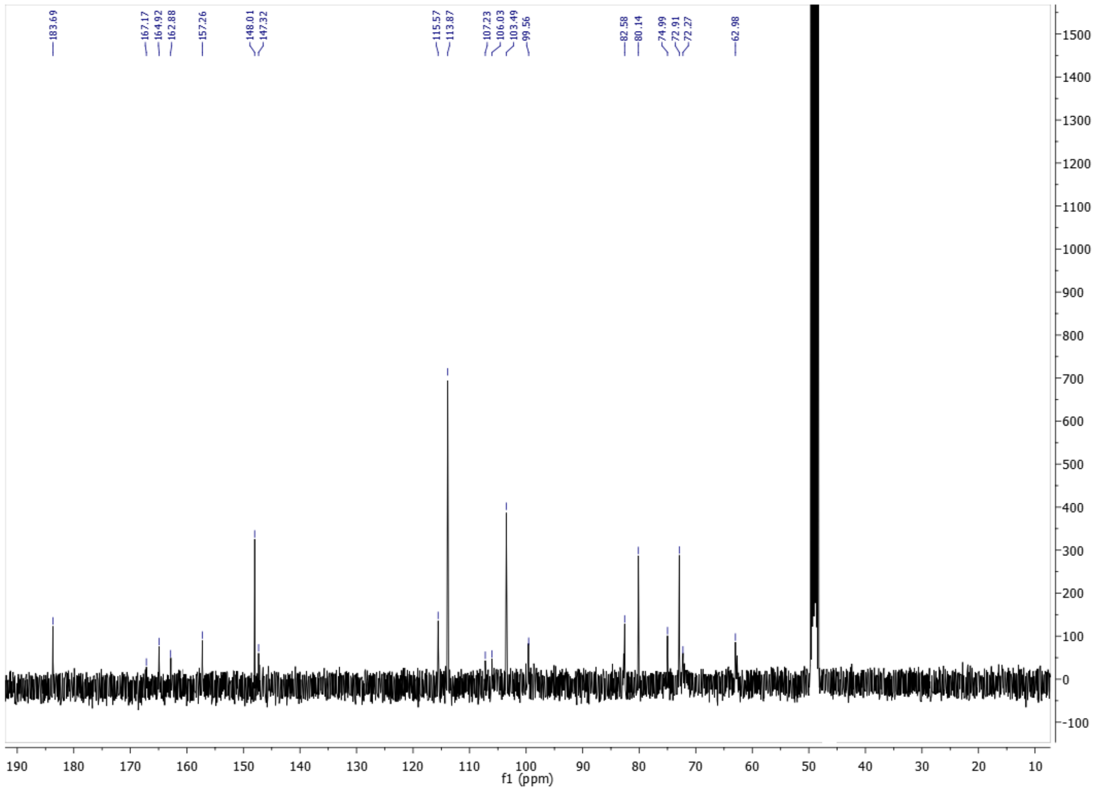

8-(β-D-Glucopyranosyl)-5,7-dihydroxy-4*H*-chromen-4-one (22)

^{1}H NMR - MeOD

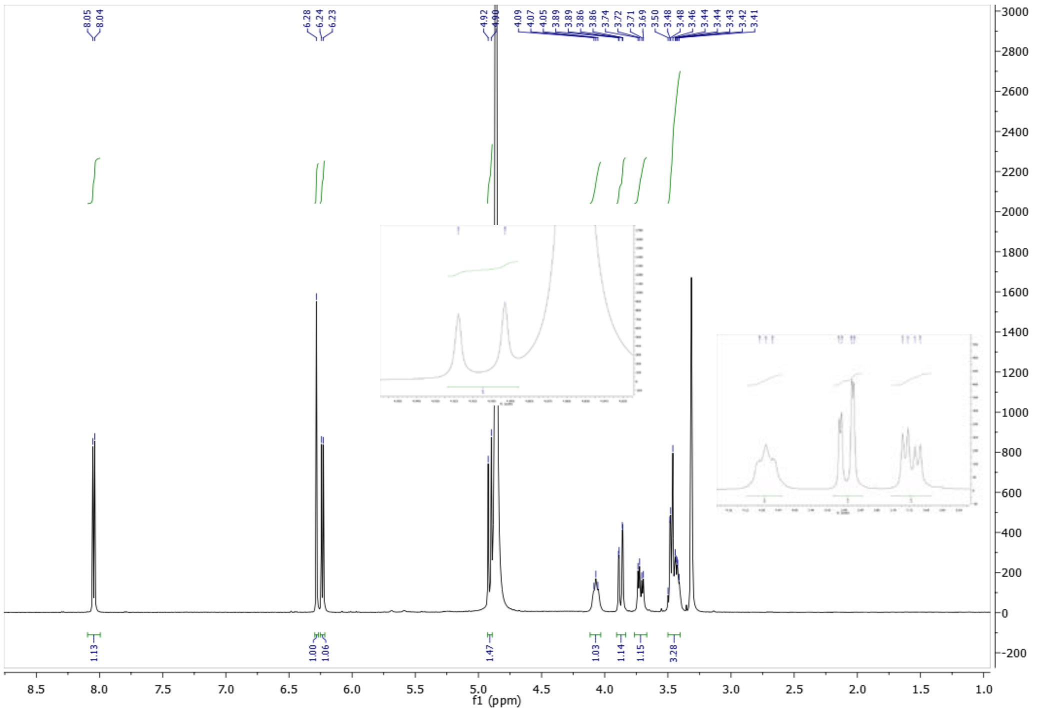

^{13}C NMR - MeOD

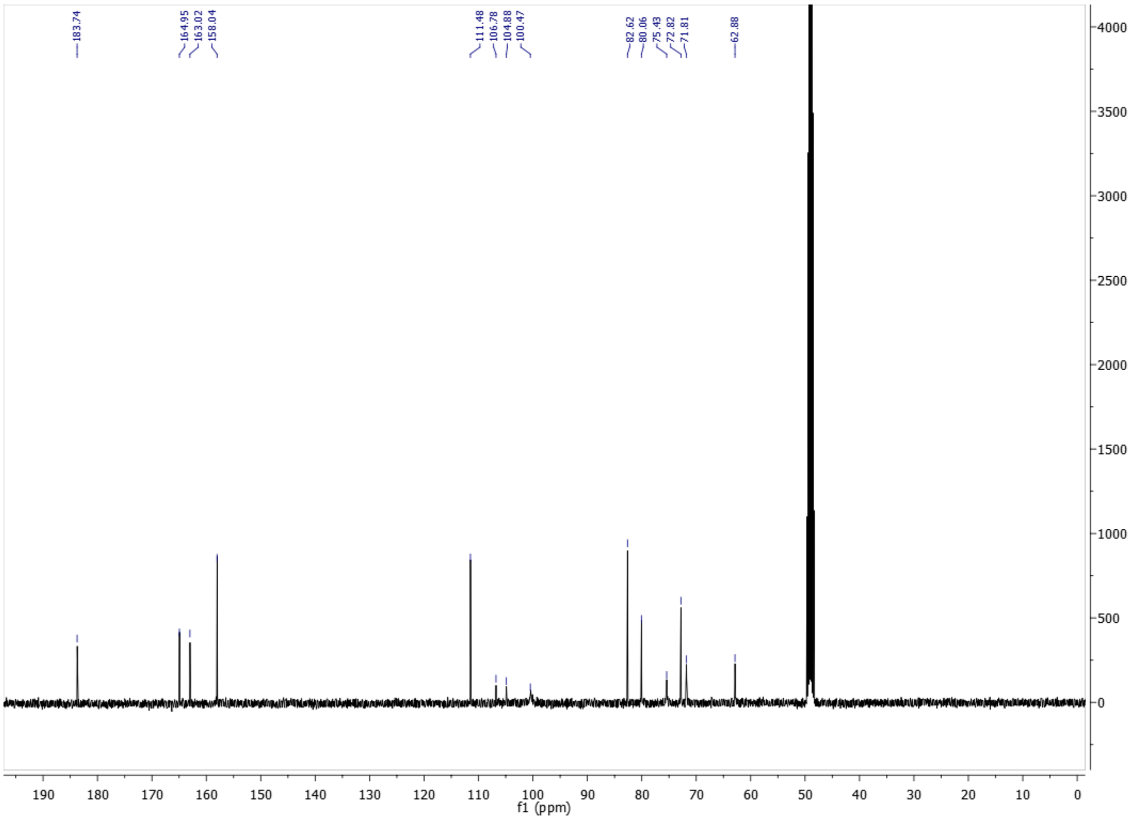

References

1. Ellman GL, Courtney KD, Andres Jr V, Feather-Stone RM. A new and rapid colorimetric determination of acetylcholinesterase activity. *Biochem. Pharmacol.* 1961;7:88-95.
2. Matos AM, Cristovao JS, Yashunsky DV, Nifantiev NE, Viana AS, Gomes CM, Rauter AP. Synthesis and effects of flavonoid structure variation on amyloid-beta aggregation. *Pure Appl Chem*. 2017;89(9):1305-1320.
3. Jesus AR, Dias C, Matos AM, de Almeida RF, Viana AS, Marcelo F, Ribeiro RT, Macedo MP, Airoldi C, Nicotra F, Martins A, Cabrita EJ, Jiménez-Barbero J, Rauter AP. Exploiting the therapeutic potential of 8-β-D-glucopyranosylgenistein: synthesis, antidiabetic activity, and molecular interaction with islet amyloid polypeptide and amyloid β-peptide (1-42). *J Med Chem.* 2014;57(22):9463-9472.

.
